# Time-Reduced N‑Methylation: Exploring a Faster Synthesis Technique

**DOI:** 10.1021/acs.joc.5c00083

**Published:** 2025-05-23

**Authors:** Aleksandra Helbik-Maciejewska, Agata Gitlin-Domagalska, Mladena Glavaš, Natalia Ptaszyńska, Dawid Dębowski, Anna Łęgowska, Krzysztof Rolka

**Affiliations:** † Department of Molecular Biochemistry, Faculty of Chemistry, 49646 University of Gdansk, Wita Stwosza 63, 80-308 Gdańsk, Poland; ‡ Department of Organic Chemistry and Biochemistry, Rud̵er Bošković Institute, Bijenička c. 54, 10 000 Zagreb, Croatia

## Abstract

Backbone N-methylation is a pivotal peptide modification that enhances lipophilicity, metabolic stability, and binding affinity or specificity, thereby improving bioactive peptides’ bioavailability. Substitution of a backbone amide hydrogen with a methyl group is a three-step procedure which is fully integrated with solid-phase peptide synthesis strategy and usually takes about 4 h. We have revolutionized this process by optimizing the method and slashing the total N-methylation procedure time from 4 h to just 40 min. Moreover, we demonstrate that N-methylation can be equally efficient regardless of the laboratory equipment used, such as a standard laboratory shaker, microwave synthesizer, or common ultrasonic bath. Our study not only results in acceleration of the N-methylation process during solid-phase peptide synthesis but also offers a flexible choice of laboratory equipment, making peptide modifications more efficient and achievable.

## Graphical abstract

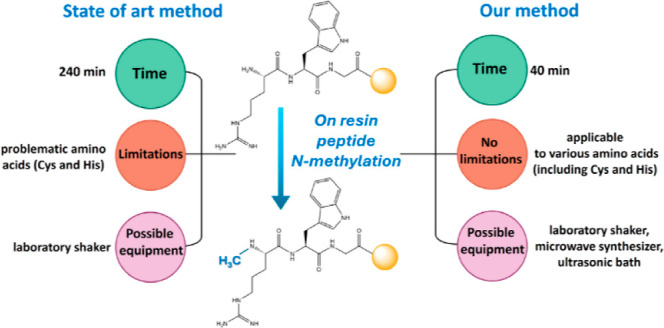

## Introduction

N-Methylation is a prevalent modification in peptides and proteins, occurring in both prokaryotes and eukaryotes. The post-translational N-methylation of proteins is crucial in many biological pathways, including the regulation of mitosis and DNA repair.^1^ In nature, N-methylation confers new physiochemical properties to peptides and proteins. There are many naturally derived, linear and cyclic, multiply N-methylated peptides, e.g., hemiasterlin, thiocoraline, or echinomycin.^2^ Therefore, it is not surprising that methylation is frequently employed as a chemical modification to mimic natural processes. Methylation of the nitrogen atom in the peptide backbone is the most popular type of alkylation due to its versatility and relatively straightforward implementation. The rationale for introducing this modification in biologically active peptides includes the increase of membrane permeability and metabolic stability, as well as modulation of binding affinity or specificity.^3,4^

Numerous examples demonstrate the positive effect of peptide N-methylation, including brain-penetrating neurotensin (NT) analogues with higher affinity to the NT receptor,^5,6^ modulation of urotensin II receptor ligand activity,^7^ enhancement of the proteolytic resistance of analogues of substance P,^8^ and improvement of the oral bioavailability of somatostatin analogues without changing their biological properties.^9^ Among the peptide drugs approved by the U.S. Food and Drug Administration (FDA), three are N-methylated peptides: cyclosporine A, voclosporin, and dactinomycin.^10^ Cyclosporine A is a well-known immunosuppressive drug that is applied after transplantation to prevent allograft rejection. It consists of 11 amino acid residues, and it possesses seven N-methylated amide nitrogen atoms. Despite its size, it exhibits significant oral bioavailability. For comparison, its analogue, cyclosporin E, which differs only by the lack of an *N*-methyl group at the Val11 residue, displays an order of magnitude lower permeability in the parallel artificial membrane permeability assay (PAMPA).^4,11,12^ Another derivative of cyclosporin is voclosporin, which contains an additional methyl group in the first amino acid side chain. It is widely used to treat immunologic disorders. Voclosporin also exhibits significant bioavailability, as confirmed by preclinical models and human studies.^4^ Dactinomycin, another multiply N-methylated peptide, is a DNA intercalator used to treat childhood-associated sarcomas, among other conditions.^13,14^ Abarelix, a drug withdrawn from the United States but approved in Germany and the Netherlands, is used in palliative treatment of advanced prostate cancer. N-Methylation, along with the introduction of nonproteinogenic amino acids, contributes to Abarelix’s higher affinity to the gonadotropin-releasing hormone (GnRH) receptors than GnRH. Due to this modification, it blocks secretion of the luteinizing hormone (LH) and follicle-stimulating hormone (FSH), consequently reducing testosterone levels, which inhibits the growth of prostate cancer cells.^4,15^

Numerous methods of N-methylation have been described to date, including N-methylation of amino acid derivatives in solution^16–19^ and subsequent coupling of them to the peptide or modifications carried out directly on a solid support during peptide chain elongation.^20–23^ In our group, we routinely and successfully utilize the method described by Naoum et al.^23^ (Scheme 1A). This three-step procedure consists of sulfonylation, methylation, and desulfonylation and is fully compatible with solid-phase peptide synthesis (SPPS). Naoum’s^23^ procedure derives from the method proposed earlier by Biron and co-workers^21^ (Scheme 1B), which, in turn, was based on the method described by Miller and Scanlan^20^ (Scheme 1C). Naoum et al.^23^ took a closer look at each step in the process and identified that N-methylation of an amine group adjacent to a hindered moiety is particularly challenging. They determined sulfonylation as the most vulnerable stage in the procedure. Consequently, after testing numerous conditions and reagents, they replaced 2,4,6-collidine (collidine), which was used in the previous procedures,^21,22^ with 4-dimethylaminopyridine (DMAP). The DMAP-mediated sulfonylation proved to be significantly more efficient for sterically hindered amines and resulted in a remarkably increased reaction yield. However, the authors focused mainly on the selection of amines and did not optimize the reaction time, instead applying the conditions from Miller and Scanlan’s work,^20^ including a 120 min duration for the sulfonylation step.^20^ The entire N-methylation procedure,^23^ excluding the time necessary for washing, is a time-consuming process which takes 4 h. Literature^21,22^ indicates that each step of the procedure might be completed in significantly less time. Therefore, our goal was to accelerate the entire N-methylation process while maintaining a high efficiency.

**Scheme 1:**
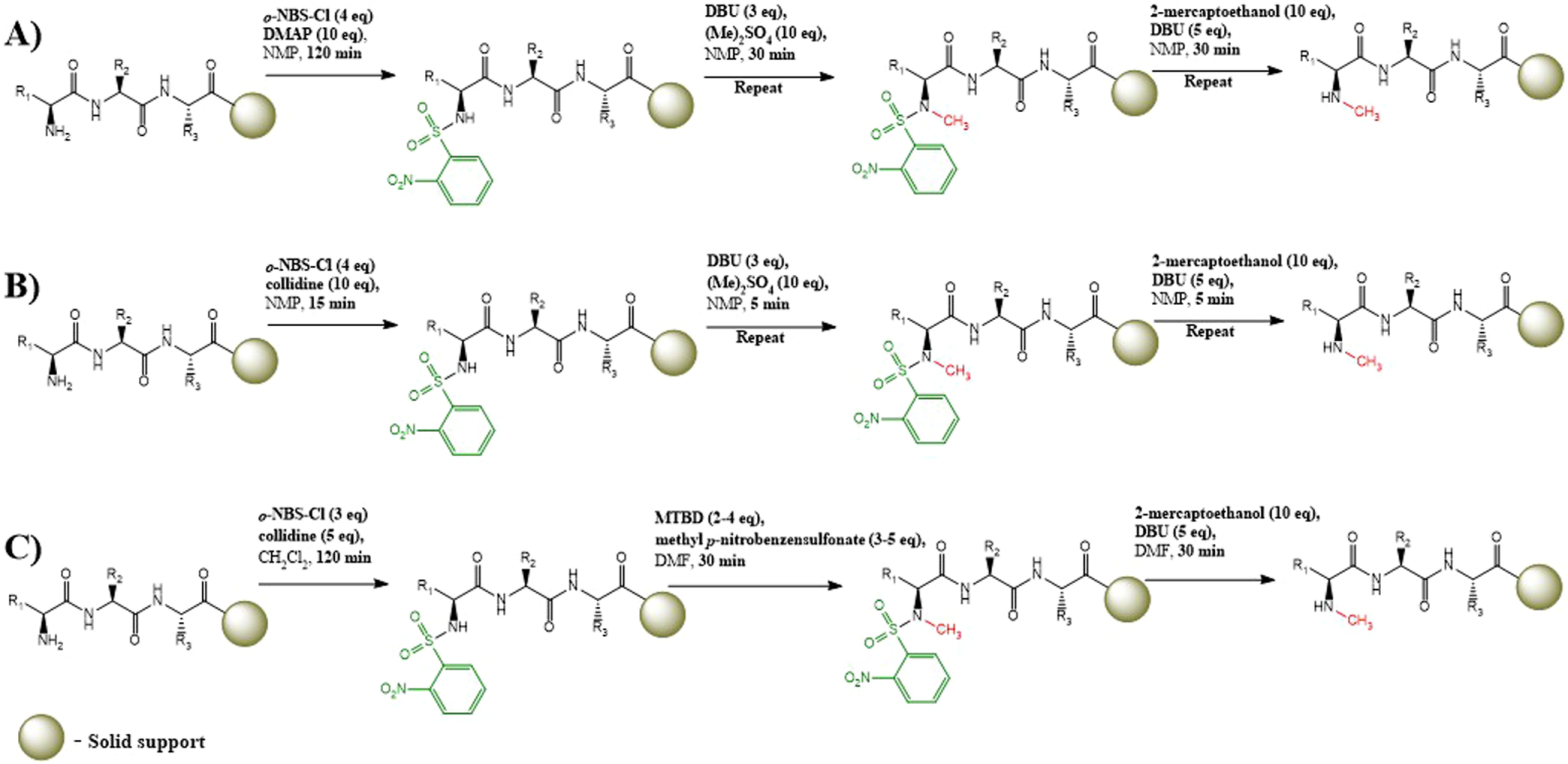
General Procedure N-Methylation Methods Described by (A) Naoum et al.,^23^ (B) Biron et al.,^21^ and (C) Miller and Scanlan^20^

Recently, Wołczański et al.,^24^ reported an accelerated Fmoc-amino acid coupling, completed within 5 min, using ultrasonic agitation (UA) instead of standard shaking (SS). Encouraged by these promising results, we decided to investigate whether UA may replace SS during N-methylation procedure steps described by Naoum et al.^23^ and thereby shorten the reaction time. By applying ultrasonic irradiation, we reduced the total N-methylation procedure time from 4 h to 40 min (Scheme 2). Moreover, we compared its effectiveness to procedures performed by shaking using a laboratory shaker at room temperature i.e. SS and using a microwave assisted peptide synthesizer (MW).

**Scheme 2:**
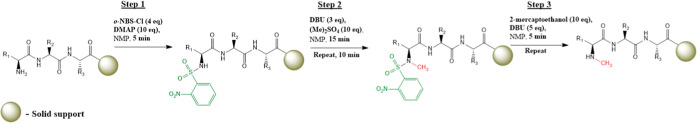
General Procedure of the Time Reduced on Resin Methylation of the peptide α-Amino Group Developed in This Paper

## Results and Discussion

Similarly to Naoum and co-workers,^23^ the tripeptide amide RWG-NH_2_ (peptide **1**) was selected as a model compound for N-methylation optimization. Multiple, successful SPPS peptide syntheses utilizing UA^24^ in our lab for standard procedures, such as coupling and deprotection, encouraged us to also perform on-resin N-methylation^23^ using ultrasonic mixing. Initially, we decreased reaction time to 60 min for the first step, 15 min for the second step (repeated twice), and 15 min for the third step (repeated twice). N-Methylation steps are described in detail in a further part of this paragraph. As a result, we obtained the N-methylated peptide, with a HPLC purity of the crude final product (85%) comparable to that obtained by Naoum et al.^23^ using a laboratory shaker. Encouraged by these results, we decided to further shorten the reaction time when using UA (Table S1), obtaining 5 min for the first step, 25 min for the second step, and 10 min for the third step. UA seems to be a valuable alternative to SS because of its accessibility (possibility to use common ultrasonic baths, which are standard equipment in laboratories) and its proven ability to accelerate peptide synthesis.^24^

After selecting the optimal reaction conditions (i.e., 5 min for the first step, 25 min for the second step, and 10 min for the third step), we replaced the N-terminal Arg with His, Asp, Glu, Ser, Tyr, Trp, Cys, Ala, and Phe (Scheme 3A) to evaluate the applicability and efficiency of our optimized method to various amino acids. Finally, similarly to Naoum et al.^23^ (Scheme 3B), we applied our procedure to obtain an analogue of somatostatin 1SW-1, which contains three methylation sites in its sequence (Scheme 3C).

**Scheme 3:**
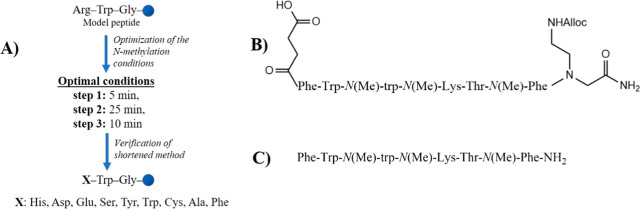
(A) Overview of the Optimization Process; (B) Peptide 1SW-1 Used by Naoum et al.^23^ as a Model to Confirm the Efficiency of the Method; (C) Analogue of Peptide 1SW-1 Synthesized by Us to Confirm the Efficiency of Our Time-Reduced Method

The first step in N-methylation is sulfonylation, which involves protection of the N-terminal α-amine group with the *o*-nitrobenzenesulfonyl (*o*-NBS) group using *o*-nitrobenzenesulfonyl chloride (*o*-NBS-Cl). In the procedure described by Naoum et al.,^23^ this process took 2 h. At the beginning, 10 equiv of DMAP and 4 equiv of *o*-NBS-Cl are dissolved in *N*-methylpyrrolidine (NMP) and mixed well for preactivation. The mixture is then added to the peptide resin. Using the same equivalents of the above-mentioned reagents combined with UA, we gradually reduced the time required for completion of this step (Table S1). Finally, the complete conversion was achieved in only 5 min. Lack of non-sulfonylated RWG-NH_2_ peptide was confirmed by HPLC and MS analyses after cleavage of a small amount of resin-bound peptide. Analytical RP-HPLC profiles of peptide RWG-NH_2_ (Figure S1) and sulfonylated *o*-NBS-RWG-NH_2_ (Figure S2) are shown in the Supporting Information. Further reduction of the sulfonylation time to 1 min (Table S1, entry 22) resulted in a decrease in reaction efficiency, which is confirmed by the HPLC purity of the crude N-methylated final product amounting 56% (total time for the second step was 25 min). It is worth noting that previously, it was reported that sulfonylation is completed within 15 min^21^ or after two treatments for 15 and 10 min^22^ when treating the peptide resin with a mixture of collidine and *o*-NBS-Cl.

In the second step, after introduction of the *o*-NBS group, alkylation with dimethylsulfate is performed. Initially, the peptide resin is treated with 3 equiv of the base 1,8-diazabicyclo­[5,4,0]­undec-7-ene (DBU) in NMP to deprotonate the amine group. Biron et al.^21^ reported that 3 min of reaction with DBU is sufficient to complete this reaction, which is indicated by a change in the resin color to yellow. Subsequently, 10 equiv of dimethylsulfate in NMP is added to introduce a methyl group onto the *o*-NBS-protected α-amino group. Biron et al.^21^ emphasized the importance of performing the reaction first with DBU and then with dimethylsulfate to avoid the side reaction between DBU and dimethylsulfate. According to this report, a 2 min reaction with dimethylsulfate is enough to achieve full conversion.^21^ Naoum et al.^23^ found that the time indicated by Biron and co-workers^21^ is too short. They proposed 3 min of preactivation with DBU, followed by a 30 min reaction with dimethylsulfate, and repetition of the procedure.^23^ According to our study applying UA, this step should be repeated twice: first, 3 min deprotonation with DBU, followed by a 15 min (10 min for the repetition) reaction with dimethylsulfate. Such conditions are optimal for various amino acid residues, resulting in HPLC purity of crude product ranges of 74–99% (Table 1, peptides **1**–**11**, Figure 1). In our study utilizing UA, the shorter, 2 min, N-methylation time proposed by Biron et al.^21^ turned out to be insufficient for the arginine residue, and HPLC purity of the final crude N-methylated peptide was only 34% (Table S1, entry 14). The use of MW-assisted synthesis at 40 °C (2 min) caused increased HPLC purity of the crude product (75%; Table S1, entry 20), although methylation was still more effective when a longer (25 min) reaction time was applied. Methylation for 2 min at 70 °C using MW resulted in even more contaminated product (41% HPLC purity of the crude product, Table S1, entry 21). Also, N-methylation of peptide synthesized on 2-chlorotrityl chloride resin, as reported by Biron (Table 1, peptide **2**) in such a short 2 min time using UA, led to low efficiency (59% HPLC purity of the crude product; Table S2, entry 1). Nevertheless, amino acids without sterically hindered side chains (e.g., glycine or phenylalanine) were successfully methylated after two 2 min long reactions using both UA and SS (Table 1, peptides **10** and **11**, Figure 1J,K) with efficiencies in the range of 98–100%. Moreover, it is worth noting that Biron et al.^21^ and Chatterjee et al.^22^ indicated that their procedures are not applicable for cysteine and histidine due to observed methylation of their side chains; thus, they suggest the Mitsunobu reaction for N-methylation in the case of these two residues. Analytical RP-HPLC profiles of N-methylated peptides **1–11** are shown in Figure 1. In our study, the reported side reactions were not observed for peptides bearing N-terminal Cys or His amino acids (Table 1, peptides **3** and **9**, Figure 1C,I). All applied methylation procedures (using UA, SS, and MW) generated products with the methyl group attached exclusively to the terminal α-amino group, which was confirmed by MS and HPLC analyses. The HPLC purity of the crude product for peptide **3** was 92–96%, and that for peptide **9** was 74–80%. NMR analyses of the purified products confirmed the presence of single homogeneous N-methylated peptides only (Figures S70–S72). Our result indicates that there is no need to use the highly exothermic Mitsunobu reaction^21,22^ for N-methylation of Cys and His residues, as was reported previously.^21,22^

**Table 1:** Results of Reactions Carried Out through (a) UA, (b) SS in Room Temperature, and (c) MW, According to the Optimized Procedure

peptide	sequence	HPLC purity of the crude product [%][Table-fn t1fn1]	isolated yield [%][Table-fn t1fn2]
**1**	(*N*-Me)RWG-NH_2_	(a) 83	(a) 60
		(b) 82	(b) 62
		(c) 80	(c) 55
**2**	(*N*-Me)RWG-OH	(a) 91	(a) 54
**3**	(*N*-Me)HWG-NH_2_	(a) 96	(a) 32
		(b) 93	(b) 53
		(c) 92	(c) 72
**4**	(*N*-Me)SWG-NH_2_	(a) 77	(a) 39
		(b) 88	(b) 37
		(c) 90	(c) 51
**5**	(*N*-Me)WWG-NH_2_	(a) 74	(a) 35
		(b) 86	(b) 56
**6**	(*N*-Me)YWG-NH_2_	(a) 87	(a) 45
		(b) 88	(b) 63
**7**	(*N*-Me)DWG-NH_2_	(a) 54	(a) 16
		(b) 65	(b) 16
		(c) 73	(c) 29
**8**	(*N*-Me)EWG-NH_2_	(a) 84	(a) 71
		(b) 93	(b) 42
**9**	(*N*-Me)CWG-NH_2_	(a) 78	(a) 37
		(b) 74	(b) 44
		(c) 80	(c) 49
**10**	(*N*-Me)FWG-NH_2_	(a) 98	(a) 66
		(b) 98	(b) 73
**11**	(*N*-Me)AWG-NH_2_	(a) 99	(a) 31
		(b) 100	(b) 42

x HPLC purity of the crude product determined as the percentage of the area under the peak using RP-HPLC at 214 nm (linear gradient 10–90% B for 20 min, where phase B is 80% CH_3_CN in H_2_O containing 0.1% trifluoroacetic acid).

y Isolated yield—the final yield of obtained peptides after purification.

**Figure 1:**
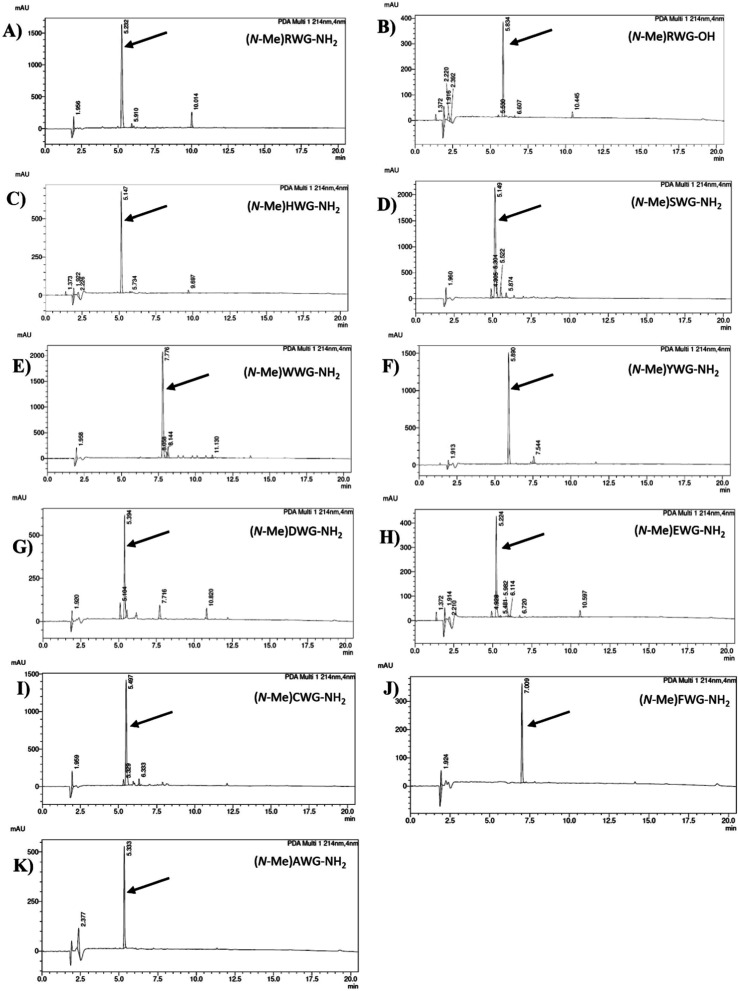
HPLC chromatograms of crude peptides **1–11**; eluent A: 0.1% TFA in H_2_O, eluent B: 0.1% TFA in 80% ACN, gradient 10–90% B in 20 min, flow rate = 1.0 mL/min, *T* = 30 °C, λ = 214 nm; the arrow indicates the final, N-methylated peptide.

The only problematic residue was aspartic acid (Table 1, peptide **7**), which was not the subject of N-methylation in previously mentioned papers.^20–23^ Remarkably, after a second DBU treatment, the peptidyl-resin resin changed color from light yellow to brown, which was not observed during N-methylation of other amino acid residues. HPLC analysis (Figure 2) revealed a high amount of impurities, and the HPLC purity of the final N-methylated crude product amounted to only 26% (Table S7, entry 1). Interestingly, we did not observe such complications during the N-methylation of glutamic acid, and its HPLC purity of the crude product was 84–93% (Table 1, peptide **8**). The probable reason was too long exposure of peptide **7** to DBU, causing side reactions such as the formation of aspartimide.^25^ An attempt to methylate peptide **7** with N-terminal Asp at a higher temperature (40 °C) resulted in even worse efficiency (11% HPLC purity of the crude product; Table S7, entry 2). The solution to this problem was abandonment of the second treatment with DBU and dimethylsulfate, which resulted in a higher 54% HPLC purity of crude N-methylated peptide **7** (synthesis with UA, Figure 1G). Better efficiency of the reaction was observed using MW for synthesis (73% HPLC purity of the crude product). All analytical RP-HPLC profiles of N-methylated peptide (*N*-Me)­DWG-NH_2_ are shown in the SI (Figures S31–S38).

**Figure 2:**
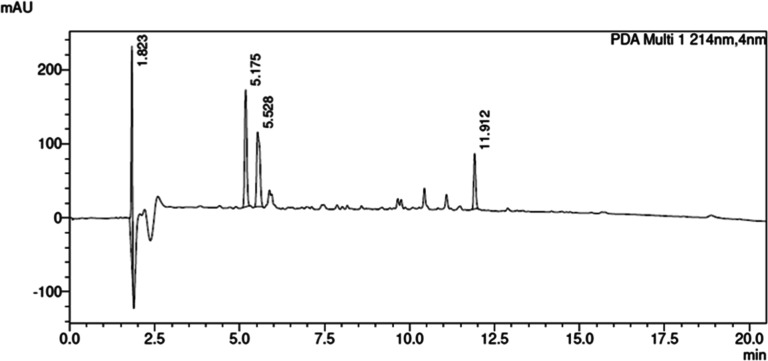
HPLC chromatogram of crude peptide (*N*-Me)­DWG-NH_2_ obtained according to Table S7, entry 1, *t*_R_ = 5.175 min (major), *t*_R_ = 5.528 min ((*N*-Me)­DWG-NH_2_), *t*_R_ = 11.912 min (minor); eluent A: 0.1% TFA in H_2_O, eluent B: 0.1% TFA in 80% ACN, gradient 10–90% B in 20 min, flow rate = 1.0 mL/min, *T* = 30 °C, λ = 214 nm.

The purpose of the final step is removal of the *o*-NBS protecting group using 10 equiv of 2-mercaptoethanol and 5 equiv of DBU in NMP. This step is usually easy to monitor due to the appearance of yellow-green color after addition of the reaction mixture to the peptide-resin, which results from the formation of the side product, 2-(2-nitrophenylthio)­ethanol.^20^ According to previously described procedures,^20^ either a single or repeated-twice^23^ 30 min long reaction is required to complete *o*-NBS removal. However, according to our research, a 5 min reaction repeated twice using UA is enough to remove the *o*-NBS group from the peptide, thereby confirming the reports of Biron’s group.^21^ It is worth noting that the mixture of 2-mercaptoethanol and DBU removes the *o*-NBS group exclusively from tertiary amines, which, here, means the N-methylated N-terminal amino group.^26^ In the case of uncompleted N-methylation, i.e., the presence of secondary amines, the *o*-NBS group would not be removed using the aforementioned mixture. Our results confirm this. In most cases, the crude, final products obtained by optimized procedures showed a small peak on HPLC chromatograms corresponding to the peptide with the *o*-NBS group (confirmed by MS), indicating incomplete N-methylation. Nevertheless, we did not observe double methylated peptide (which would indicate ineffective sulfonylation with *o*-NBS) or peptides with both methyl and *o*-NBS groups (indicating ineffective desulfonylation). This confirms the proposed 5 min time is sufficient for the complete removal of the *o*-NBS group (desulfonylation) from the N-methylated peptide.

Compared to Naoum’s procedure,^23^ we shortened the first step to 5 min, the second step to 25 min, and the third step to 10 min (Table S1). This means we reduced the total N-methylation procedure time from 4 h to 40 min. Initially, we attributed this undoubtful success to UA, whose advantages were described previously for coupling and deprotection during SPPS.^24^

We performed analogous syntheses, applying the reduced, optimized reaction times, but using a laboratory shaker (SS) (Table S1, entry 18) and microwave irradiation in a peptide synthesizer (MW) (Table S1, entry 19) instead of UA. We achieved nearly identical results for reactions conducted at room temperature with SS (Table 1, peptides **1–11**), UA (peptides **1–11**), and MW (Table 1, peptides **1**, **3**, **4**, **7**, and **9**). These findings suggest that in the case of N-methylation, UA does not accelerate the process (as it was reported for SPPS^24^) but can serve as an alternative method for conducting the reaction. However, regardless of the method used (UA, SS, and MW), it is essential to thoroughly shake the resin with the reaction mixture at each step of the procedure. Additionally, the resin must be washed carefully between consecutive steps of the procedure, i.e., five times with NMP. This thorough washing is crucial to avoid potential side reactions and to ensure the production of a highly pure product. HPLC chromatograms of the N-methylated peptide (*N*-Me)­RWG-NH_2_ obtained using UA, SS, and MW are shown in Figure 3.

**Figure 3:**
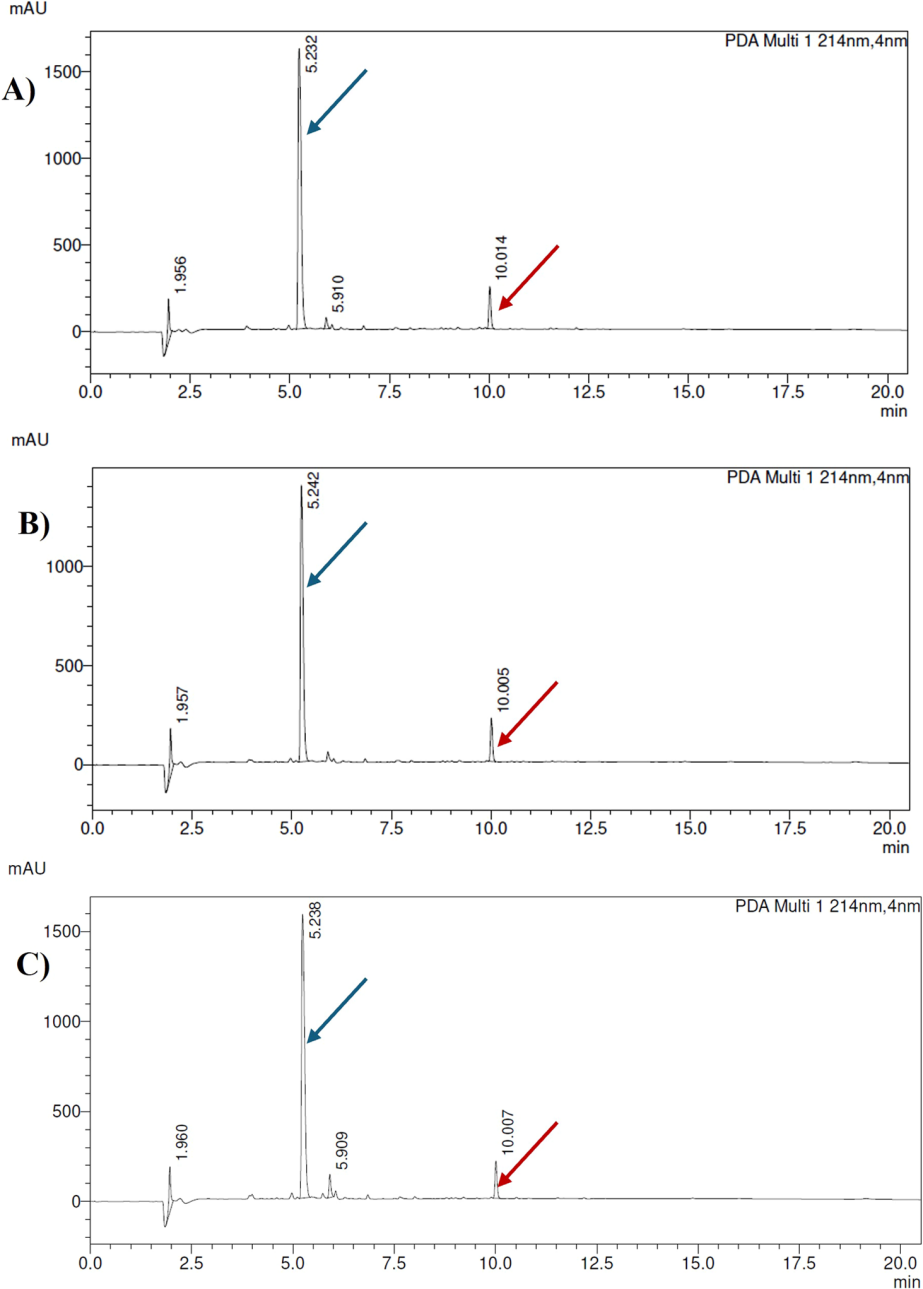
HPLC chromatograms of crude peptide (*N*-Me)­RWG-NH_2_ obtained using (A) UA, (B) SS, and (C) MW; the blue arrow indicates peptide (*N*-Me)­RWG-NH_2_, and the red arrow indicates *o*-NBS-RWG-NH_2_; eluent A: 0.1% TFA in H_2_O, eluent B: 0.1% TFA in 80% ACN, gradient 10–90% B in 20 min, flow rate = 1.0 mL/min, *T* = 30 °C, λ = 214 nm.

To validate the applicability of our time-reduced N-methylation procedure, we synthesized the analogue of the 1SW-1 peptide (Scheme 3C) using UA (Table 2, entry 1) and MW at 40 °C (Table 2, entry 2). We are aware that coupling to the N-methylated residues might be more challenging than standard coupling to the free –NH_2_ group.^27^ However, Naoum et al.^23^ did not report any difficulties. In the procedure described by Biron et al.,^21^ coupling to the N-methylated residue was completed in most cases after 3 h of SS.^21^ Chatterjee et al.^22^ mentioned difficult coupling of Fmoc-Thr­(*t*Bu)-OH to the N-methylated Phe, requiring a 3 h reaction, repeated 3 times to achieve satisfactory efficiency.^22^ We also observed that coupling of Thr­(*t*Bu) to *N*(Me)­Phe in the analogue of the 1SW-1 peptide was more challenging compared to other couplings. We applied UA or MW and repeated the coupling reaction 4 times for 15 min each to complete it (monitored by chloranil and Kaiser tests). Indeed, in the final crude product, we observed a minor impurity corresponding to a peptide deprived of Thr (Figure 4). Coupling of other amino acid derivatives to the N-methylated amino acids was not so demanding, and it took 15 min and was repeated twice. We also synthesized an analogue of the 1SW-1 peptide, performing N-methylation applying conditions as reported by Biron et al.^21^ (Table 2, entry 3). In all cases, we obtained analogues of 1-SW1 with HPLC profiles of crude products (Figure 4) comparable with those shown by Naoum et al.^23^

**Table 2:** Syntheses of an Analogue of Peptide 1SW-1. Consecutive couplings were performed using UA (entries 1 and 3) or MW (entry 2)

entry	time of sulfonylation [min]	time of methylation [min]	time of desulfonylation [min]	N-methylation method	HPLC purity of crude product [%]
**1**	5	15 + 10	5 + 5	UA	57
**2**	5	15 + 10	5 + 5	MW	57
**3**	15 (reaction with 2,4,6-collidine)	2 + 2	5 + 5	SS	65

**Figure 4:**
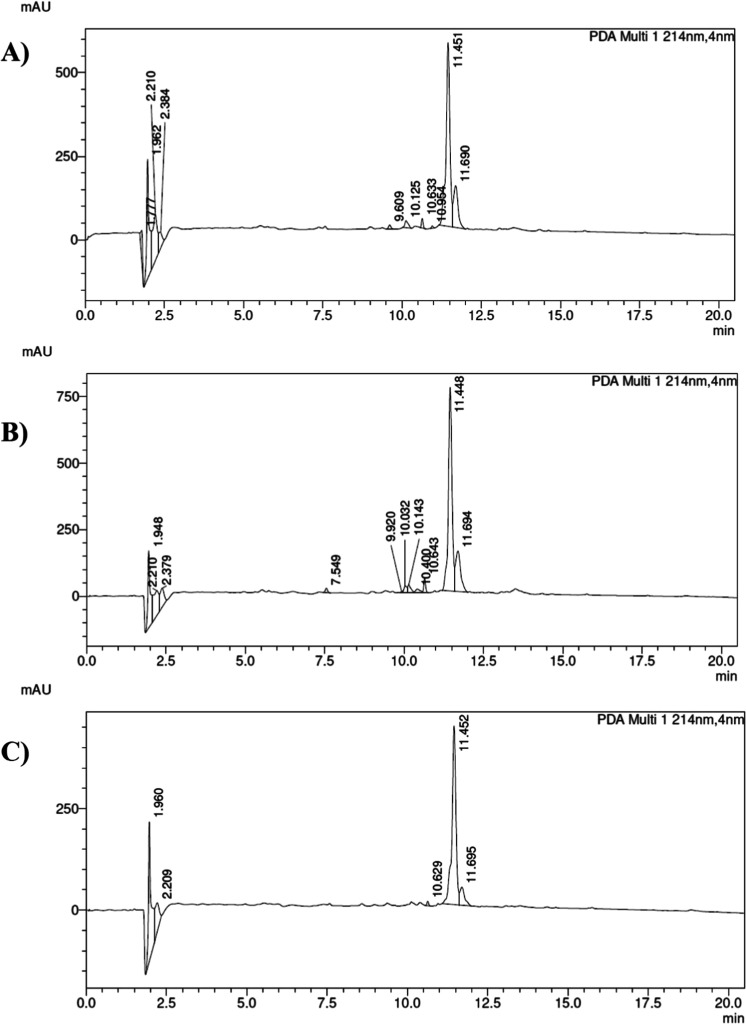
HPLC chromatograms of the crude analogue of peptide 1-SW1 obtained using (A) UA, *t*_R_ = 11.451 min (major, 1-SW1), 11.690 min (minor, 1-SW1 without Thr); (B) MW, *t*_R_ = 11.448 min (major, 1-SW1), 11.694 min (minor, 1-SW1 without Thr); and (C) method described by Biron et al.,^21^ *t*_R_ = 11.452 min (major, 1-SW1), 11.695 min (minor, 1-SW1 without Thr); eluent A: 0.1% TFA in H_2_O, eluent B: 0.1% TFA in 80% ACN, gradient 10–90% B in 20 min, flow rate = 1.0 mL/min, *T* = 30 °C, λ = 214 nm.

## Conclusion

We demonstrated that the N-methylation procedure^23^ can be significantly shortened from 4 h to 40 min while maintaining very good to excellent yields of the desired products. We compared on-resin N-methylation of diverse amino acid derivatives applying various laboratory equipment, including a laboratory shaker, peptide synthesizer with microwave irradiation, and ultrasonic bath. Our findings revealed that UA is not responsible for accelerating the N-methylation reaction; however, it significantly reduces the time required for difficult coupling to N-methylated (secondary amine) groups.

N-Methylation of diverse amino acid derivatives resulted in similar effectiveness and purity, with the exception of Asp. This residue required special treatment, although the reaction time was still reduced as compared to the procedure applied by Naoum’s group.^23^ Additionally, we did not observe N-methylation of Cys or His side chains reported by Biron et al.^21^ and Chatterjee et al.,^22^ which was confirmed by NMR analyses.

Furthermore, we successfully obtained multiply N-methylated peptide (three N-methylated positions) applying our procedure, achieving HPLC purity of the crude product of 57% for peptides methylated using either UA or MW. Remarkably, our shortened method of N-methylation does not require advanced devices and can be applied using various laboratory equipment, including a common laboratory shaker, ultrasonic bath, or peptide synthesizer with microwave irradiation. This simple and time-saving method of peptide methylation on solid support offers a viable alternative to purchasing expensive methylated amino acid derivatives. It is important to underline that the reduction of time of the N-methylation means not only a quicker method but also shorter exposure to harmful chemicals.

## Supplementary Material



## Data Availability

The data underlying this study are available in the published article and its Supporting Information.

## Notes

Additional experimental details, materials, and methods, including 1H and 13C spectra, RP-HPLC chromatograms, and MS analyses (PDF)
