# Supplementary material for: Time-Reduced N‑Methylation: Exploring a Faster Synthesis Technique
Source: J Org Chem. 2025 May 23;90(22):7182–90. doi: 10.1021/acs.joc.5c00083 (PMC12150311; doi:10.1021/acs.joc.5c00083)
Supplement: Supplementary file 1 [file jo5c00083_si_001.pdf]

Supporting information

Time-Reduced N-Methylation: Exploring Faster Synthesis Technique

Aleksandra Helbik-Maciejewska[1], Agata Gitlin-Domagalska[1]*, Mladena Glavaš[2], Natalia Ptaszyńska[1], Dawid Dębowski[1], Anna Łęgowska[1], Krzysztof Rolka[1]

[1]Department of Molecular Biochemistry, Faculty of Chemistry, University of Gdansk, Wita Stwosza 63 80-308 Gdańsk, Poland

[2] Department of Organic Chemistry and Biochemistry, Ruđer Bošković Institute, Bijenička c. 54, 10 000 Zagreb, Croatia

*Correspondence: agata.domagalska@ug.edu.pl

Table of Contents

Experimental section S1

Chemical synthesis of peptides S1

Three steps optimized *N*-methylation procedure S2

***o*-NBS protection (optimized procedure)** S2

***N*-methylation (optimized procedure)** S2

***o*-NBS deprotection (optimized procedure)** S2

Chemical synthesis of peptide 1-SW1 .... S2

Purification of peptides S3

NMR characterization of peptides S4

Optimization of peptides *N*-methylation S7

HPLC chromatograms of synthesized peptides S10

MS analyses of synthesized peptides S38

NMR spectra of selected peptides S45

Experimental section

Chemical synthesis of peptides

All peptides were synthesized manually on solid support using the standard 9-fluorenylmethoxycarbonyl (Fmoc) chemistry and applying ultrasonic agitation (UA) for mixing. Compounds were synthesized on rink amide methylbenzhydrylamine (Fmoc-MBHA, GL Biochem Shanghai) resin (loading 0.646 mmol/g), except peptide **2** which was synthesized on 2-chlorotrityl chloride resin (GL Biochem Shanghai). All syntheses were performed in the ultrasonic bath (Polsonic Sonic 2 with ultrasonic power (peak/period) of 2×100W and ultrasonic frequency of 40 kHz). All syntheses, including methylation steps were carried out in a syringe equipped with filter. Fmoc group was removed with 20% piperidine solution in N,N-dimethyloformamide (DMF). Peptide chain elongation was performed with N,N,N′,N′-tetramethyl-O-(1H-benzotriazol-1-yl)uronium hexafluorophosphate (HBTU)/ 1-hydroxybenzotriazole (HOBt)/ N,N-diisopropylethylamine (DIPEA) or 1 O-(7-azabenzotriazol-1-yl)-N,N,N',N'-tetramethyluronium hexafluorophosphate (HATU)/ 1-hydroxy-7-azabenzotriazole (HOAt)/ N,N-diisopropylethylamine (DIPEA). Threefold equivalent of appropriate amino acid derivative was used. The coupling reaction was carried out for 15 minutes and the progress of these steps was monitored by the Kaiser and chloranil tests. The resin was washed with DMF (3 × 1 min), IsOH (3 × 1 min) and DCM (3 × 1 min). The procedure of coupling was repeated twice. The protected Fmoc-amino acid derivates were purchased. The *C*-terminal Gly (2 mmol) in peptide **2** was attached to the 2-chlorotrityl chloride resin with presence of 1 eq of DIPEA in anhydrous dichloromethane (DCM). Peptides were cleaved from the resin with simultaneous removal of protecting groups using a mixture of TFA/phenol/triisopropylosilane/H_2O (88:5:2:5, v/v/v/v).

The progress of reaction and purity of compounds were checked by RP-HPLC using Shimadzu Prominence-I LC-2050C 3D equipped with a Kinetex XB-C18 column (150 × 4.6 mm, 5 µm) and a UV-Vis detector (flow rate: 1ml/min, linear gradient 10-90% B for 20 min and detection at 214 nm). Spectroscopy mass analyses were performed using Autoflex maX MALDI-TOF spectrometer, Bruker Daltonics, Germany (applied matrix: 2,5-dihydroxybenzoic acid).

The NMR spectra were recorded on a Bruker Avance III 500 MHz under the frequencies of 500 MHz (^1H) and 126 MHz (^{13}C) in CD_3OD at room temperature. The spectra were processed in the programs MestReNova version 0.2-5475, Mestrelab Research S,L; 2009. Chemical shifts (δ) are expressed according to deuterium solvent in ppm values, and coupling constants (J) are expressed in hertz (Hz). The ^1H NMR spectra are shown as δ chemical shift/ppm (assignation, multiplicity, coupling constant, proton number). ^{13}C NMR spectra are shown as δ chemical shift/ppm. The peaks are marked as s (singlet), d (doublet), t (triplet) or m (multiplet). The NMR spectra were analyzed according to one-dimensional ^1H and ^{13}C spectra.

Three-step optimized *N*-methylation procedure

In below description term „mixing" will refer to different methods of mixing/ stirring reaction mixture with resin. *N*-Methylation procedure using ultrasonic agitation (UA) was carried with the aid of ultrasonic bath (Polsonic Sonic 2 with ultrasonic power (peak/period) of 2 x 100W and ultrasonic frequency of 40 kHz). In the case of standard shaking (SS) reaction was performed using laboratory shaker (Elpin+ type 358A). Selected peptides were also methylated using microwaves (MW) synthesizer (Biotage Initiator+SP Wave synthesizer, Biotage, Sweden) in dedicated polypropylene, open reactor vessel, with polytetrafluoroethylene (PTFE) frit (Biotage, Sweden) at 40°C and with rotational frequency 900 RPM. The reaction mixture temperature was monitored using a process graph with real time measurements of temperature and applied power.

o-NBS protection (optimized procedure)

Caution! Hazardous chemicals are used in this procedure. O-nitrobenzenesulfonyl chloride (o-NBS-Cl) is irritating for skin and eyes, dimethyl sulfate (Me_2SO_4) CAS 77-78-1, may cause cancer and is irritating for skin. 1,8-diazabicyclo[5.4.0]undec-7-ene (DBU) and 2-mercaptoethanol may cause eye damage and allergic reactions on skin.

A solution of 10 eq. *o*-nitrobenzenesulfonyl chloride (*o*-NBS-Cl; 0.052 M) and 4 eq. 4-dimethyloaminopyridine (DMAP; 0.130 M) in 1-metylo-2-pyrolidone (NMP) were added and preactivated for 1 minute by mixing. Then the cocktail was added to peptidyl-resin with free α-amino groups and mixed. After that time peptidyl-resin was forceful washed with NMP (5 × 1 min). Peptidyl-resin was filtered of on Buchner's funnel and washed with NMP.

N-methylation (optimized procedure)

A solution of 3 eq. of 1,8-diazabicyclo [5,4,0]undec-7-ene (DBU; 0.065 M) in NMP was added to *o*-NBS protected peptide resin and mixed for 3 minutes. Then solution of 10 eq. of dimethylsulfate (0.217 M) in NMP was added and mixed for 15 minutes. After that time, resin was washed three times with NMP. Procedure was repeated, but the time of reaction with dimethylsulfate was 10 minutes in the second approach. Resin was washed well with NMP (5 × 1 min). Additionally resin was washed with NMP during filtration.

o-NBS deprotection (optimized procedure)

A solution of 10 eq. of 2-mercaptoethanol (0.217 M) and 5 eq. of DBU (0.108 M) was mixed for preactivation, than it was added to the peptide resin and mixed for 5 minutes. Resin was washed 3 times with NMP and procedure was repeated. Resin was washed well with NMP (5 × 1 min). Additionally resin was washed with NMP during filtration.

Chemical synthesis of 1-SW1 analog

Synthesis was performed using UA analogously to the syntheses of tripeptides. Peptide chain elongation was performed using HBTU/ HOBt/ DIPEA or HATU/ HOAt/ DIPEA. The coupling reaction

was carried out for 15 minutes and repeated twice. Only coupling Fmoc-Thr(tBu)-OH to *N*-methylated Phe residue required four times of coupling

Purification of peptides

Peptides **1-11** were purified by reverse-phase high performance liquid chromatography (RP-HPLC) on PLC 2050 Gilson HPLC (Gilson Glider Prep. Software (Gilson, France)) equipped with ReproSil-XR 300 C18 column (20 × 250 mm, 10 µm, 300 Å). The mobile phases were 0,1% TFA in H_2O (phase A) and 80% acetonitrile (phase B).

NMR characterization of peptides

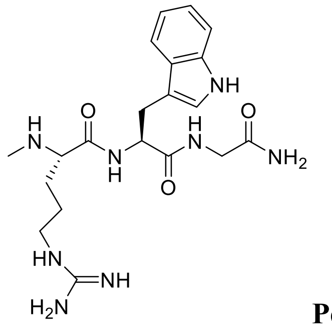

Peptide 1: ^{1}H NMR (500 MHz, CD_3OD) δ 7.64 (d, J = 8.0 Hz, 1H), 7.33 (d, J = 8.0 Hz, 1H), 7.16 (s, 1H), 7.10 (t, J = 7.5 Hz, 1H), 7.03 (t, J = 7.5 Hz, 1H), 4.84 – 4.79 (m, 2H), 3.91 (d, J = 17.0 Hz, 1H), 3.73 – 3.63 (m, 2H), 3.21 – 3.11 (m, 3H), 2.21 (s, 3H), 1.96 – 1.80 (m, 2H), 1.71 – 1.56 (m, 2H); ^{13}C {^{1}H} NMR (126 MHz, CD_3OD) δ 174.1, 173.9, 168.5, 158.7, 138.1, 128.6, 124.8, 122.6, 119.9, 119.4, 112.4, 110.6, 62.2, 56.1, 43.1, 41.7, 31.9, 29.0, 28.6, 24.9.

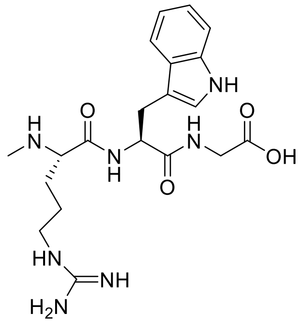

Peptide 2: ^{1}H NMR (500 MHz, CD_3OD) δ 7.67 (d, J = 8.0 Hz, 1H), 7.32 (d, J = 8.0 Hz, 1H), 7.17 (s, 1H), 7.09 (t, J = 7.5 Hz, 1H), 7.03 (t, J = 7.5 Hz, 1H), 4.98 – 4.94 (m, 1H), 3.94 (d, J = 17.0 Hz, 1H), 3.87 (d, J = 17.0 Hz, 1H), 3.65 – 3.60 (m, 1H), 3.40 – 3.36 (m, 1H), 3.19 – 3.09 (m, 3H), 2.08 (s, 3H), 1.92 – 1.79 (m, 2H), 1.68 – 1.57 (m, 2H); ^{13}C {1H} NMR (126 MHz, CD_3OD) δ 173.9, 173.1, 168.0, 158.7, 138.1, 128.7, 124.8, 122.6, 119.9, 119.5, 112.4, 110.9, 62.3, 55.4, 42.1, 41.7, 31.8, 29.4, 28.6, 24.9.

^{1}H NMR (500 MHz, DMSO-d6) δ 10.83 (s, 1H), 8.83 (d, J = 8.5 Hz, 1H), 8.43 (s, 1H), 8.06 (s, 1H), 7.68 (d, J = 8.0 Hz, 1H), 7.31 (d, J = 8.0 Hz, 1H), 7.15 (d, J = 2.2 Hz, 1H), 7.07 – 7.03 (m, 1H), 6.99 – 6.96 (m, 1H), 4.83 – 4.77 (m, 1H), 3.78 – 3.69 (m, 2H), 3.58 – 3.52 (m, 1H), 3.21 (dd, J = 14.5 Hz, J = 4.5 Hz, 2H), 3.09 – 3.02 (m, 2H), 2.96 – 2.90 (m, 1H), 1.91 (s, 3H), 1.72 – 1.58 (m, 2H), 1.51 – 1.40 (m, 2H); ^{13}C {^{1}H} NMR (126 MHz, DMSO-d6) δ 171.1, 156.8, 136.1, 127.2, 123.9, 120.9, 118.6, 118.3, 111.3, 109.8, 60.4, 53.3, 30.8, 28.3, 27.4, 23.9.

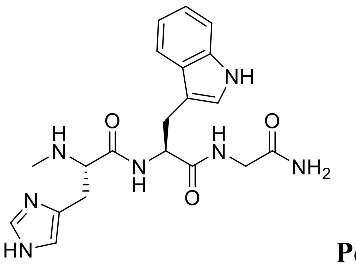

Peptide 3: ^{1}H NMR (500 MHz, CD_3OD) δ 8.69 (s, 1H), 7.63 (d, J = 8.0 Hz, 1H), 7.42 (s, 1H), 7.34 (d, J = 8.0 Hz, 1H), 7.16 (s, 1H), 7.13 – 7.09 (m, 1H), 7.05 – 7.02 (m, 1H), 4.81 – 4.79 (m, 2H), 3.98 (t, J = 5.9 Hz, 1H), 3.94 (s, 0.5H), 3.81 (s, 0.5H), 3.68 (d, J = 17.0 Hz, 1H), 3.44 – 3.39 (m, 1H), 3.30 – 3.26 (m, 1H), 3.18 – 3.11 (m, 1H), 2.31 (s, 3H); ^{13}C {^{1}H} NMR (126 MHz, CD_3OD) δ 174.6, 173.9, 167.5, 138.1, 136.1, 128.6, 127.6, 124.9, 122.7, 120.0, 119.4, 112.4, 110.4, 61.3, 56.2, 43.2, 32.1, 29.0, 26.8.

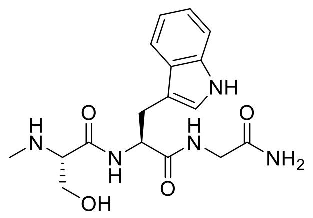

Peptide 4: ^{1}H NMR (500 MHz, CD_3OD) δ 7.65 (d, J = 8.0 Hz, 1H), 7.35 (d, J = 8.0 Hz, 1H), 7.17 (s, 1H), 7.14 – 7.09 (m, 1H), 7.07 – 7.03 (m, 1H), 4.83 – 4.78 (m, 1H), 3.97 – 3.83 (m, 3H), 3.75 – 3.69 (m, 2H), 3.41 – 3.37 (m, 1H), 3.23 – 3.13 (m, 1H), 2.33 (s, 3H); ^{13}C {^{1}H} NMR (126 MHz, CD_3OD) δ 173.9, 167.5, 128.8, 124.8, 122.6, 119.9, 119.4, 112.4, 110.8, 64.3, 60.9, 56.2, 43.1, 32.0, 28.9.

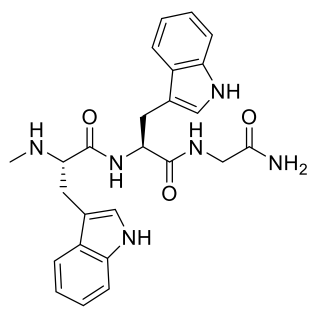

Peptide 5: ^{1}H NMR (500 MHz, CD_3OD) δ 10.57 – 10.32 (m, 1H), 7.62 (d, J = 8.0 Hz, 1H), 7.58 (d, J = 8.0 Hz, 1H), 7.37 (d, J = 8.0 Hz, 1H), 7.31 (d, J = 8.0 Hz, 1H), 7.17 (s, 1H), 7.15- 7.12 (m, 1H), 7.10 – 7.01 (m, 4H), 4.81 – 4.77 (m, 1H), 3.88 (t, J = 7.1 Hz, 1H), 3.79 – 3.73 (m, 1H), 3.65 – 3.60 (m, 1H), 3.34 – 3.32 (m, 1H), 3.24 – 3.19 (m, 1H), 3.09 – 3.03 (m, 1H), 2.04 (s, 3H); ^{13}C {^{1}H} NMR (126 MHz, CD_3OD) δ 173.9, 173.4, 168.6, 128.7, 128.2, 125.8, 124.9, 122.9, 122.6, 120.4, 119.9, 119.5, 118.9, 112.7, 112.4, 110.9, 107.3, 63.5, 55.9, 43.1, 32.1, 29.1, 28.0.

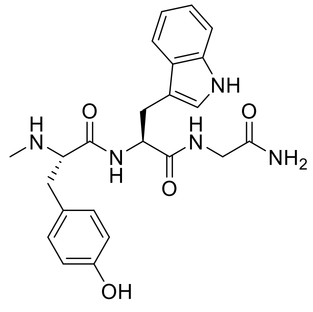

Peptide 6: ^{1}H NMR (500 MHz, CD_3OD) δ 7.83 (t, J = 5.8 Hz, 1H), 7.63 (d, J = 8.0 Hz, 1H), 7.32 (d, J = 8.0 Hz, 1H), 7.13 – 7.07 (m, 2H), 7.06 – 6.99 (m, 3H), 6.76 – 6.70 (m, 2H), 4.82 – 4.79 (m, 1H), 3.88 – 3.69 (m, 3H), 3.34 – 3.33 (m, 1H), 3.11 – 2.94 (m, 3H), 2.04 (s, 3H); ^{13}C {^{1}H} NMR (126 MHz, CD_3OD) δ 168.2, 158.3, 138.0, 131.7, 128.7, 125.3, 124.9, 122.6, 119.9, 119.5, 116.9, 112.4, 110.8, 64.2, 55.8, 43.1, 37.1, 32.1, 29.0.

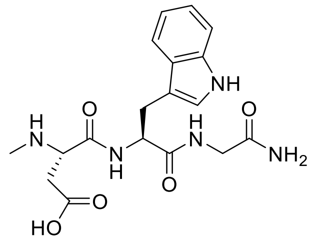

Peptide 7: ^{1}H NMR (500 MHz, CD_3OD) δ 7.64 (d, J = 8.0 Hz, 1H), 7.33 (d, J = 8.0 Hz, 1H), 7.16 (s, 1H), 7.12 – 7.08 (m, 1H), 7.06 – 7.02 (m, 1H), 4.84 – 4.79 (m, 2H), 3.92 – 3.87 (m, 2H), 3.74 (d, J = 17.0 Hz, 1H), 3.44 (dd, J = 15.0 Hz, J = 6.0 Hz, 1H), 3.15 – 3.09 (m, 1H), 2.94 (dd, J = 17.7 Hz, J = 5.5 Hz, 1H), 2.76 (dd, J = 17.7 Hz, J = 5.5 Hz, 1H), 2.18 (s, 3H); ^{13}C {^{1}H} NMR (126 MHz, CD_3OD) δ 168.2, 138.1, 128.7, 124.8, 122.6, 119.9, 119.4, 112.4, 56.2, 43.1, 32.3, 28.8.

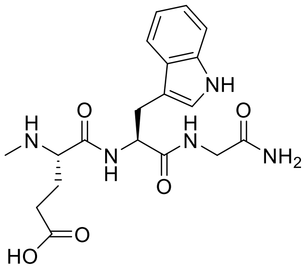

Peptide 8: ^{1}H NMR (500 MHz, CD_3OD) δ 7.66 (d, J = 8.0 Hz, 1H), 7.35 (d, J = 8.0 Hz, 1H), 7.18 (s, 1H), 7.13 – 7.09 (m, 1H), 7.07 – 7.03 (m, 1H), 4.85 – 4.79 (m, 2H), 3.91 (d, J = 17.0 Hz, 1H), 3.75 – 3.67 (m, 2H), 3.20 – 3.13 (m, 1H), 2.49 (t, J = 7.3 Hz, 2H), 2.21 (s, 3H), 2.17 – 2.03 (m, 2H); ^{13}C {^{1}H} NMR (126 MHz, CD_3OD) δ 175.9, 174.1, 173.8, 168.4, 138.1, 128.7, 124.8, 122.6, 119.9, 119.4, 112.4, 110.8, 62.1, 56.2, 43.2, 32.0, 29.9, 28.9, 26.8.

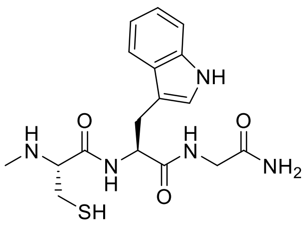

Peptide 9: ^{1}H NMR (500 MHz, CD_3OD) δ 7.63 (d, J = 8.0 Hz, 1H), 7.33 (d, J = 8.0 Hz, 1H), 7.16 (s, 1H), 7.10 (t, J = 7.5 Hz, 1H), 7.04 (t, J = 7.5 Hz, 1H), 4.79 – 4.74 (m, 2H), 3.91 – 3.83 (m, 2H), 3.67 (d, J = 17.0 Hz, 1H), 3.18 – 3.13 (m, 1H), 3.09 – 3.03 (m, 1H), 3.00 – 2.94 (m, 1H), 2.32 (s, 3H); ^{13}C {^{1}H} NMR (126 MHz, CD_3OD) δ 173.9, 167.5, 138.1, 128.7, 124.8, 122.6, 119.9, 119.4, 112.4, 110.7, 63.7, 56.4, 43.2, 32.2, 28.8, 25.4.

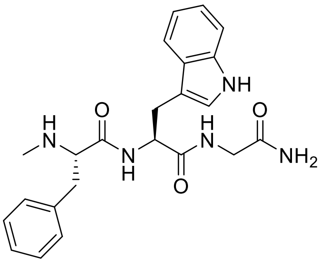

Peptide 10: ^{1}H NMR (500 MHz, CD_3OD) δ 7.63 (d, J = 8.0 Hz, 1H),7.35 – 7.27 (m, 4H), 7.24 – 7.18 (m, 2H), 7.13 – 7.06 (m, 2H), 7.05 – 7.01 (m, 1H), 4.82 – 4.78 (m, 1H), 3.89 – 3.81 (m, 2H), 3.70 (d, J = 17.0 Hz, 1H), 3.34 – 3.33 (m, 1H), 3.15 – 3.04 (m, 3H), 2.07 (s, 3H); ^{13}C {^{1}H} NMR (126 MHz, CD_3OD) δ 174.8, 173.3, 167.9, 138.0, 134.9, 130.6, 130.2, 128.9, 128.7, 124.9, 122.6, 119.9, 119.5, 112.4, 110.8, 63.9, 55.8, 43.1, 37.9, 32.1, 29.0.

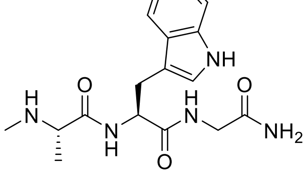

Peptide 11: ^{1}H NMR (500 MHz, CD_3OD) δ 7.63 (d, J = 8.0 Hz, 1H), 7.33 (d, J = 8.0 Hz, 1H), 7.15 (s, 1H), 7.12 – 7.07 (m, 1H), 7.05 – 7.01 (m, 1H), 4.77 – 4.73 (m, 1H), 3.92 – 3.85 (m, 1H), 3.73 – 3.66 (m, 2H), 3.34 – 3.32 (m, 1H), 3.18 – 3.11 (m, 1H), 2.28 (s, 3H), 1.47 – 1.45 (m, 3H); ^{13}C {^{1}H} NMR (126 MHz, CD_3OD) δ 173.9, 173.8, 169.9, 138.1,128.7, 124.8, 122.6, 119.9, 119.4, 112.4, 110.8, 58.4, 56.2, 43.1, 31.6, 29.0, 16.4.

Optimization of on-resin *N*-methylation

Table S1. Optimization of model peptide RWG-NH_2 *N*-methylation.

Entry	Time of sulfonylation [min]	Time of methylation [min]	Time of desulfonylation [min]	Method	HPLC purity of crude product [%]
**1**	60	15 + 15	5 + 5	UA	85
**2**	60	15 + 15	15 + 15	UA	n/d[a]
**3**	45	15 + 15	15 + 15	UA	n/d[a]
**4**	45	30 + 15	15 + 15	UA	88
**5**	30	30 + 15	15 + 15	UA	90
**6**	20	30 + 15	5 + 5	UA	87
**7**	10	30 + 15	5 + 5	UA	89
**8**	10	30 + 15	5 + 5	SS	90
**9**	10	20 + 15	5 + 5	UA	90
**10**	10	30 + 10	5 + 5	UA	88
**11**	10	15 + 15	5 + 5	UA	85
**12**	10	2 + 2	5 + 5	UA	58
**13**	5	15 + 15	5 + 5	UA	76
**14**	5	2 + 2	5 + 5	UA	34
**15**	5	15 + 5	5 + 5	UA	84
**16**	5	10 + 5	5 + 5	UA	75
**17**	**5**	**15 + 10**	**5 + 5**	UA	**83**
**18**	**5**	**15 + 10**	**5 + 5**	SS	**82**
**19**	**5**	**15 + 10**	**5 + 5**	MW	**80**
**20**	5	2 + 2	5 + 5	MW	75
**21**	5	2 + 2 (70°C)	5 + 5	MW	41
**22**	1	15 + 10	5 + 5	UA	56

[a]n/d - not determined due to the presence of high amount of impurities

Table S2. Optimization of RWG-OH *N*-methylation.

Entry	Time of sulfonylation [min]	Time of methylation [min]	Time of desulfonylation [min]	Method	HPLC purity of crude product [%]
**1**	5	2 + 2	5 + 5	UA	**59**
**2**	5	15 + 10	5 + 5	UA	**91**

Table S3. Optimization of HWG-NH_2 *N*-methylation.

Entry	Time of sulfonylation [min]	Time of methylation [min]	Time of desulfonylation [min]	Method	HPLC purity of crude product [%]
**1**	10	30 + 15	5 + 5	UA	**93**
**2**	10	30 + 15	5 + 5	SS	**90**
**3**	5	15 + 10	5 + 5	UA	**96**
**4**	5	15 + 10	5 + 5	SS	**93**
**5**	5	15 + 10	5 + 5	MW	**92**

Table S4. Optimization of SWG-NH_2 *N*-methylation.

Entry	Time of sulfonylation [min]	Time of methylation [min]	Time of desulfonylation [min]	Method	HPLC purity of crude product [%]
**1**	10	30 + 15	5 + 5	UA	**94**
**2**	10	30 + 15	5 + 5	SS	**90**
**3**	5	15 + 10	5 + 5	UA	**77**
**4**	5	15 + 10	5 + 5	SS	**88**
**5**	5	15 + 10	5 + 5	MW	**90**

Table S5. Optimization of WWG-NH_2 *N*-methylation.

Entry	Time of sulfonylation [min]	Time of methylation [min]	Time of desulfonylation [min]	Method	HPLC purity of crude product [%]
**1**	10	30 + 15	5 + 5	UA	**94**
**2**	10	30 + 15	5 + 5	SS	**84**
**3**	5	15 + 10	5 + 5	UA	**74**
**4**	5	15 + 10	5 + 5	SS	**86**

Table S6. Optimization of YWG-NH_2 *N*-methylation.

Entry	Time of sulfonylation [min]	Time of methylation [min]	Time of desulfonylation [min]	Method	HPLC purity of crude product [%]
**1**	10	30 + 15	5 + 5	UA	**91**
**2**	10	30 + 15	5 + 5	SS	**95**
**3**	5	15 + 10	5 + 5	UA	**87**
**4**	5	15 + 10	5 + 5	SS	**88**

Table S7. Optimization of DWG-NH_2 *N*-methylation.

Entry	Time of sulfonylation [min]	Time of methylation [min]	Time of desulfonylation [min]	Method	HPLC purity of crude product [%]
**1**	10	30 + 15	5 + 5	UA	**26**
**2**	10 (40°C)	30 + 15 (40°C)	5 + 5 (40°C)	UA	**11**
**3**	10	30	5 + 5	UA	**72**
**4**	5	15	5 + 5	UA	**54**
**5**	5	15	5 + 5	SS	**65**
**6**	5	15	5 + 5	MW	**73**

Table S8. Optimization of EWG-NH_2 *N*-methylation.

Entry	Time of sulfonylation [min]	Time of methylation [min]	Time of desulfonylation [min]	Method	HPLC purity of crude product [%]
**1**	10	30 + 15	5 + 5	UA	**93**
**2**	5	15 + 10	5 + 5	UA	**84**
**3**	5	15 + 10	5 + 5	SS	**93**

Table S9. Optimization of CWG-NH_2 *N*-methylation.

Entry	Time of sulfonylation [min]	Time of methylation [min]	Time of desulfonylation [min]	Method	HPLC purity of crude product [%]
**1**	10	30 + 15	5 + 5	UA	**82**
**2**	10	30 + 15	5 + 5	SS	**84**
**3**	5	15 + 10	5 + 5	UA	**78**
**4**	5	15 + 10	5 + 5	SS	**74**
**5**	5	15 + 10	5 + 5	MW	**80**

Table S10. Optimization of FWG-NH_2 *N*-methylation.

Entry	Time of sulfonylation [min]	Time of methylation [min]	Time of desulfonylation [min]	Method	HPLC purity of crude product [%]
**1**	5	5 + 5	5 + 5	UA	**94**
**2**	5	2 + 2	5 + 5	UA	**98**
**3**	5	2 + 2	5 + 5	SS	**98**

Table S11. Optimization of AWG-NH_2 *N*-methylation.

Entry	Time of sulfonylation [min]	Time of methylation [min]	Time of desulfonylation [min]	Method	HPLC purity of crude product [%]
**1**	5	5 + 5	5 + 5	UA	**86**
**2**	5	2 + 2	5 + 5	UA	**99**
**3**	5	2 + 2	5 + 5	SS	**100**

HPLC chromatograms of synthesized peptides

HPLC conditions: Shimadzu Prominence-I LC-2050C 3D equipped with a Kinetex XB-C18 column (150 × 4.6 mm, 5 µm) and a UV-Vis detector. Flow rate: 1ml/min, linear gradient 10-90% B for 20 min, detection at 214 nm, 30°C. Crude peptides were dissolved in H_2O/ACN, filtrated though 0.45 µm regenerated cellulose filters. Purified peptides were dissolved in H_2O and injected.

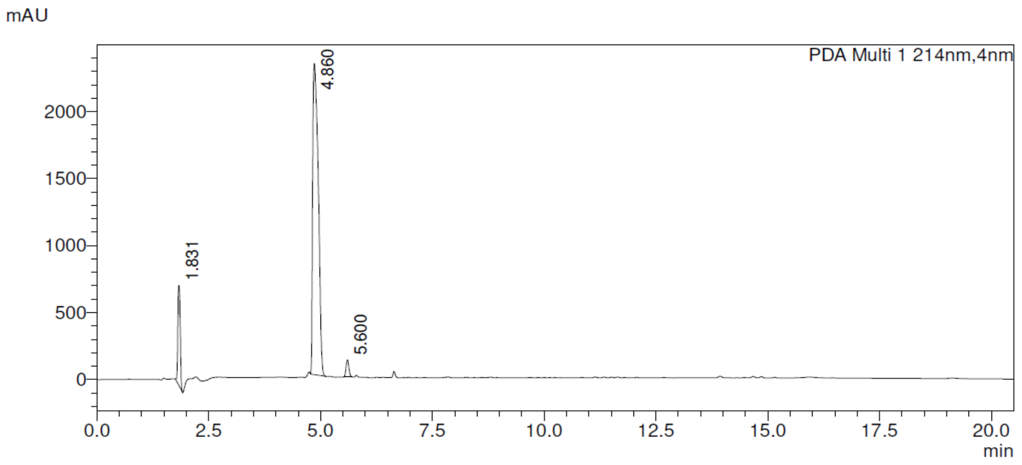

Figure S1. HPLC chromatogram of crude peptide RWG-NH_2, (eluent A 0.1%TFA in H_2O, eluent B 0.1%TFA in 80% ACN, gradient 10-90%B in 20 min, flow rate = 1.0 mL/min, T= 30°C, λ = 214 nm) t_R = 4.860 min (major, RWG-NH_2), t_R = 5.600 min (minor).

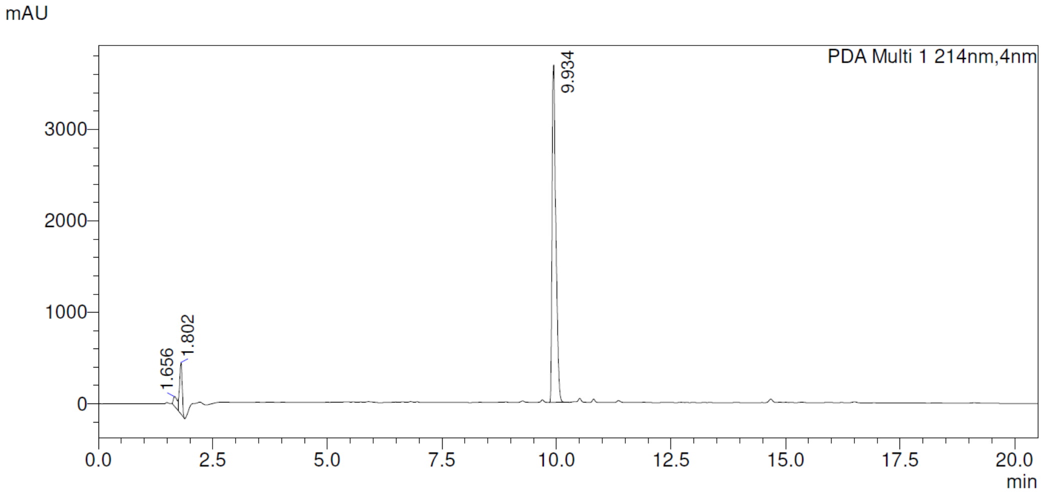

Figure S2. HPLC chromatogram of crude peptide *o*-NBS-RWG-NH_2, (eluent A 0.1%TFA in H_2O, eluent B 0.1%TFA in 80% ACN, gradient 10-90%B in 20 min, flow rate = 1.0 mL/min, T= 30°C, λ = 214 nm) t_R = 9.934 min (*o*-NBS-RWG-NH_2).

Me-RWG-NH_2

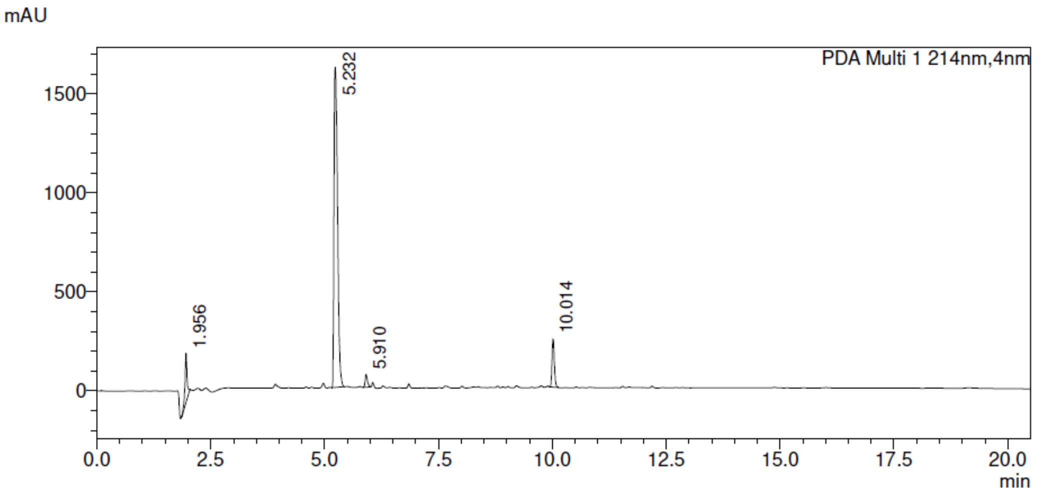

Figure S3. HPLC chromatogram of crude peptide (*N*-Me)RWG-NH_2 (Table S1, entry 17), (eluent A 0.1%TFA in H_2O, eluent B 0.1%TFA in 80% ACN, gradient 10-90%B in 20 min, flow rate = 1.0 mL/min, T= 30°C, λ = 214 nm) t_R = 5.232 min ((*N*-Me)RWG-NH_2), t_R = 5.910 min (minor), t_R = 10.014 min (*o*-NBS-RWG-NH_2)

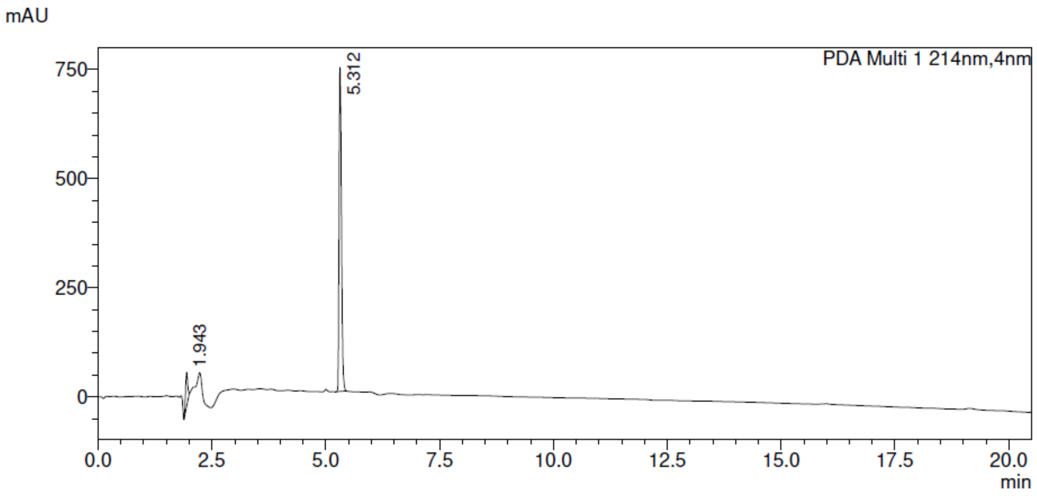

Figure S4. HPLC chromatogram of purified peptide (*N*-Me)RWG-NH_2 (Table S1, entry 17), (eluent A 0.1%TFA in H_2O, eluent B 0.1%TFA in 80% ACN, gradient 10-90%B in 20 min, flow rate = 1.0 mL/min, T= 30°C, λ = 214 nm) t_R = 5.312 min ((*N*-Me)RWG-NH_2).

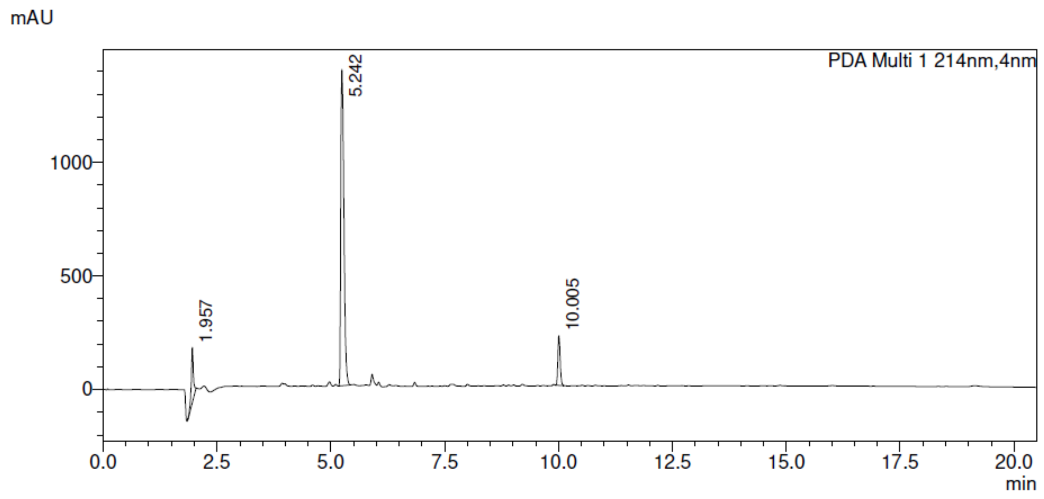

Figure S5. HPLC chromatogram of crude peptide (*N*-Me)RWG-NH_2 (Table S1, entry 18), (eluent A 0.1%TFA in H_2O, eluent B 0.1%TFA in 80% ACN, gradient 10-90%B in 20 min, flow rate = 1.0 mL/min, T= 30°C, λ = 214 nm) t_R = 5.242 min (major, (*N*-Me)RWG-NH_2), t_R = 10.005 min (minor, *o*-NBS-RWG-NH_2).

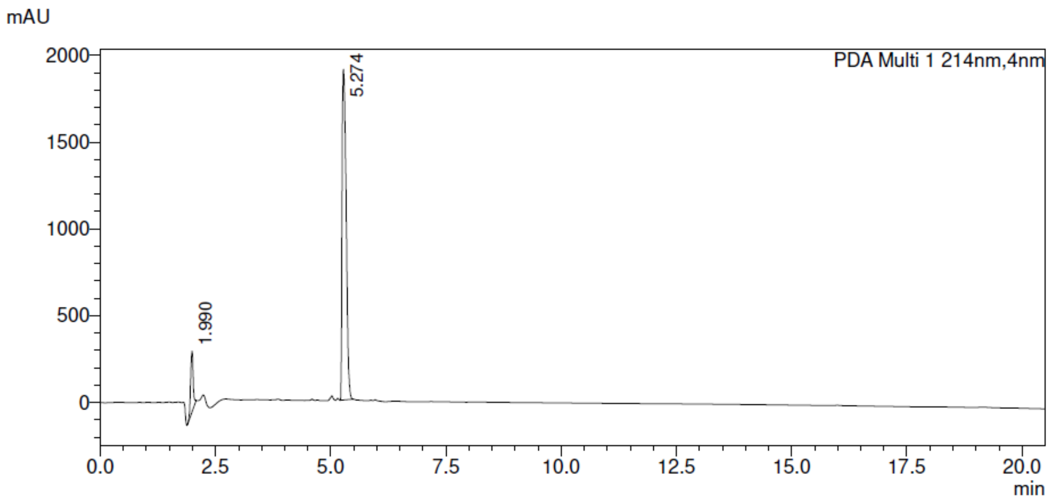

Figure S6. HPLC chromatogram of purified peptide (*N*-Me)RWG-NH_2 (Table S1, entry 18), (eluent A 0.1%TFA in H_2O, eluent B 0.1%TFA in 80% ACN, gradient 10-90%B in 20 min, flow rate = 1.0 mL/min, T= 30°C, λ = 214 nm) t_R = 5.274 min ((*N*-Me)RWG-NH_2).

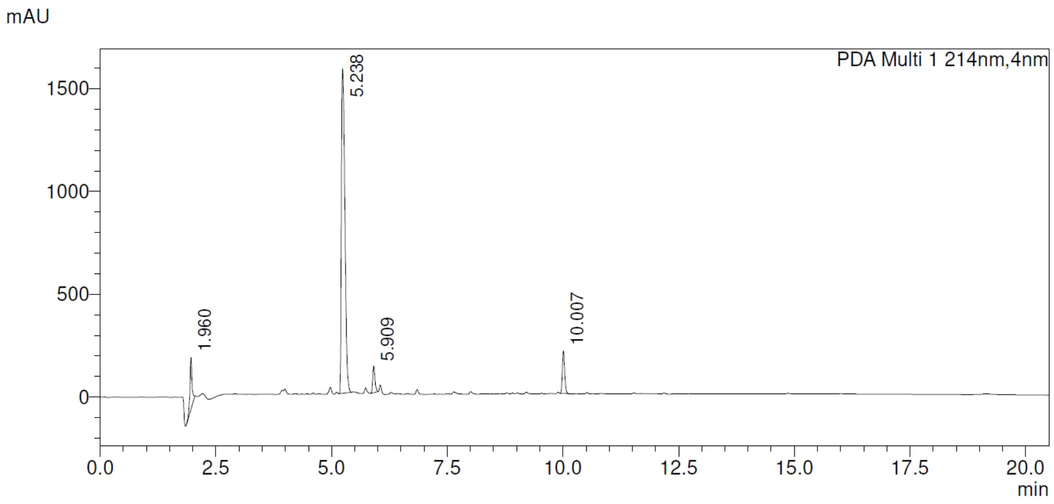

Figure S7. HPLC chromatogram of crude peptide (*N*-Me)RWG-NH_2 (Table S1, entry 19), (eluent A 0.1%TFA in H_2O, eluent B 0.1%TFA in 80% ACN, gradient 10-90%B in 20 min, flow rate = 1.0 mL/min, T= 30°C, λ = 214 nm) t_R = 5.238 min (major, (*N*-Me)RWG-NH_2), t_R = 5.909 min (minor), t_R = 10.007 min (minor, *o*-NBS-RWG-NH_2).

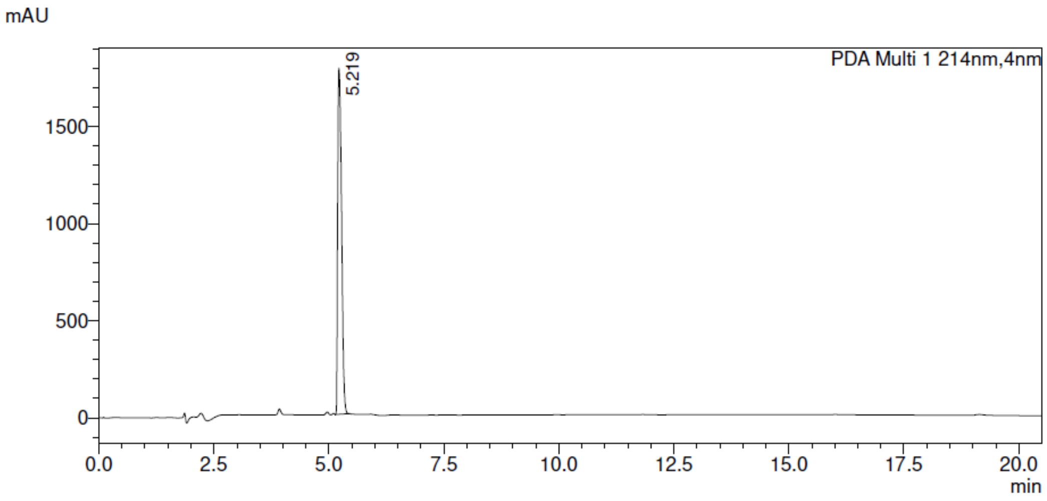

Figure S8. HPLC chromatogram of purified peptide (*N*-Me)RWG-NH_2 (Table S1, entry 19), (eluent A 0.1%TFA in H_2O, eluent B 0.1%TFA in 80% ACN, gradient 10-90%B in 20 min, flow rate = 1.0 mL/min, T= 30°C, λ = 214 nm) t_R = 5.219 min (major).

Me-RWG-OH

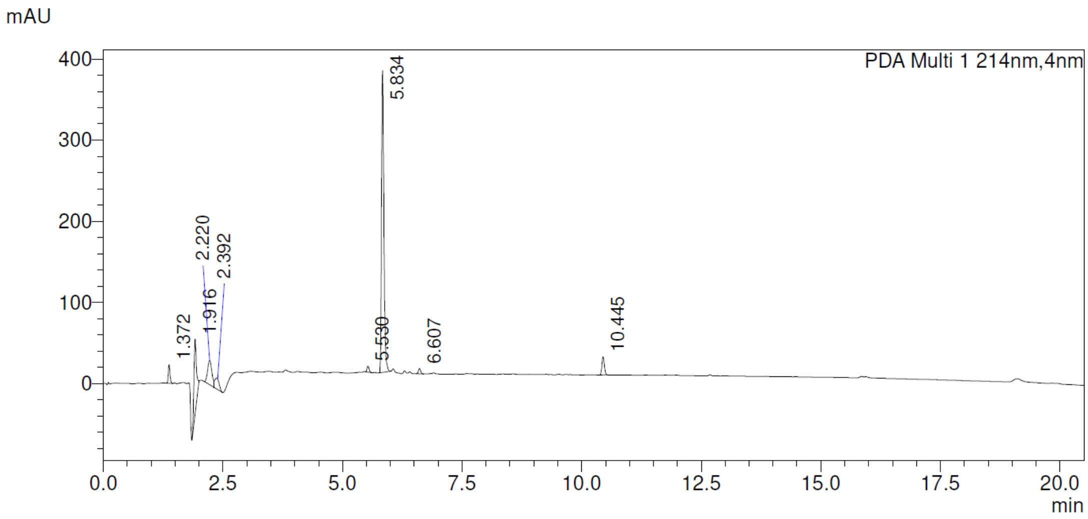

Figure S9. HPLC chromatogram of crude peptide (*N*-Me)RWG-OH (Table S2, entry 2), (eluent A 0.1%TFA in H_2O, eluent B 0.1%TFA in 80% ACN, gradient 10-90%B in 20 min, flow rate = 1.0 mL/min, T= 30°C, λ = 214 nm) t_R = 5.834 min (major, (*N*-Me)RWG-OH), t_R = 6.607 (minor), t_R = 10.445 min (minor, *o*-NBS-RWG-NH_2).

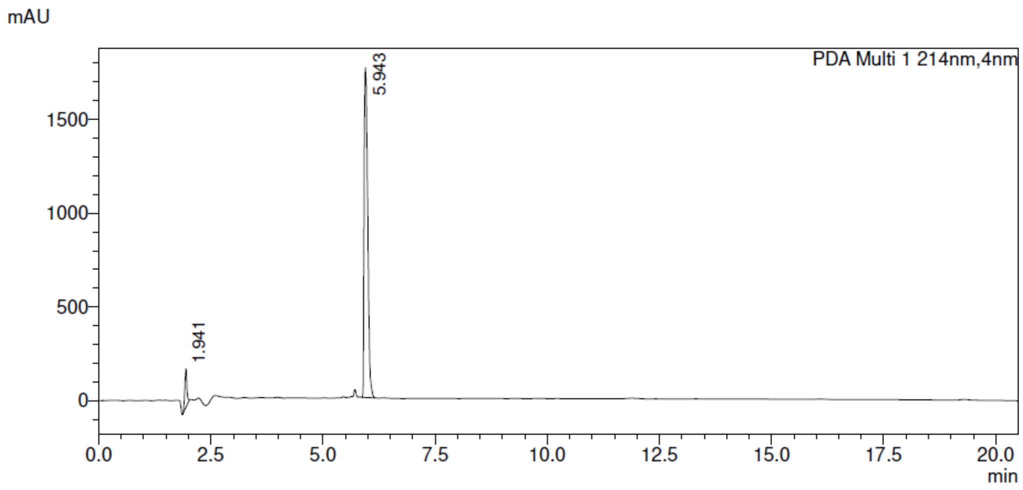

Figure S10. HPLC chromatogram of purified peptide (*N*-Me)RWG-OH (Table S2, entry 2), (eluent A 0.1%TFA in H_2O, eluent B 0.1%TFA in 80% ACN, gradient 10-90%B in 20 min, flow rate = 1.0 mL/min, T= 30°C, λ = 214 nm) t_R = 5.943 min (major).

Me-HWG-NH_2

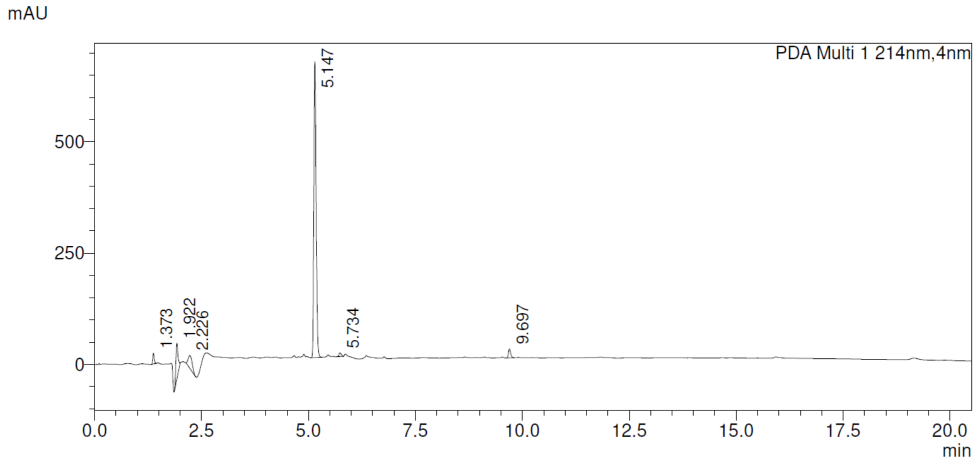

Figure S11. HPLC chromatogram of purified peptide (*N*-Me)HWG-NH_2 (Table S1, entry 3), (eluent A 0.1%TFA in H_2O, eluent B 0.1%TFA in 80% ACN, gradient 10-90%B in 20 min, flow rate = 1.0 mL/min, T= 30°C, λ = 214 nm) t_R = 5.147 min (major, (*N*-Me)HWG-NH_2), t_R = 9.697 (minor).

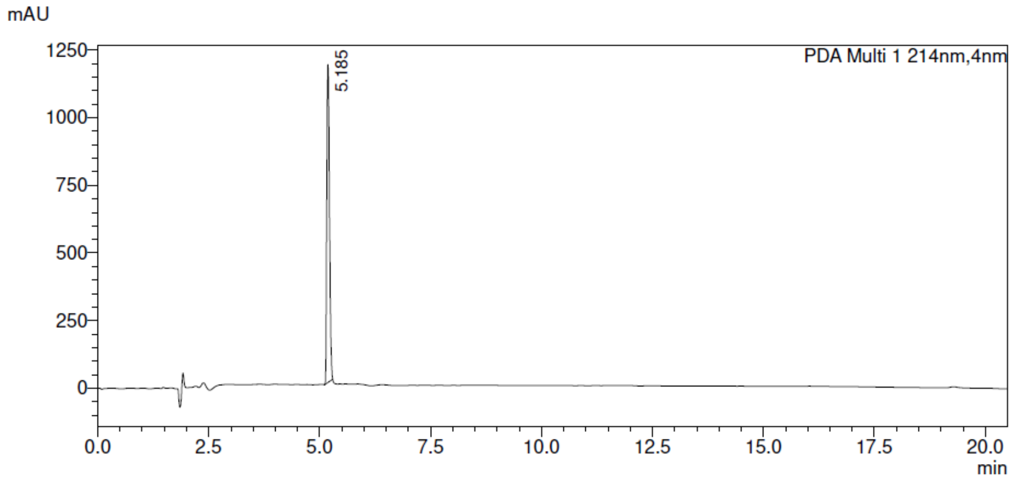

Figure S12. HPLC chromatogram of purified peptide (*N*-Me)HWG-NH_2 (Table S3, entry 3), (eluent A 0.1%TFA in H_2O, eluent B 0.1%TFA in 80% ACN, gradient 10-90%B in 20 min, flow rate = 1.0 mL/min, T= 30°C, λ = 214 nm) t_R = 5.185 min (major).

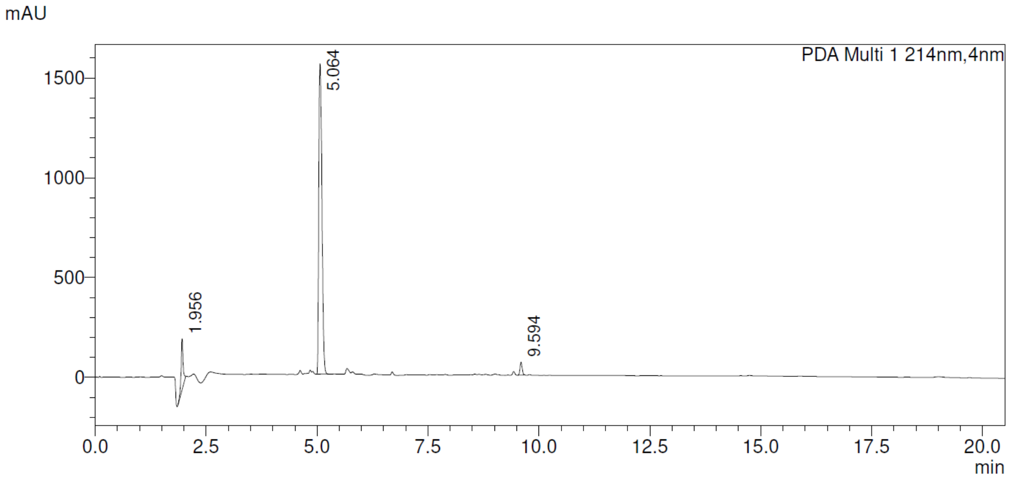

Figure S13. HPLC chromatogram of crude peptide (*N*-Me)HWG-NH_2 (Table S3, entry 4), (eluent A 0.1%TFA in H_2O, eluent B 0.1%TFA in 80% ACN, gradient 10-90%B in 20 min, flow rate = 1.0 mL/min, T= 30°C, λ = 214 nm) t_R = 5.064 min (major, (*N*-Me)HWG-NH_2), t_R = 9.594 min (minor).

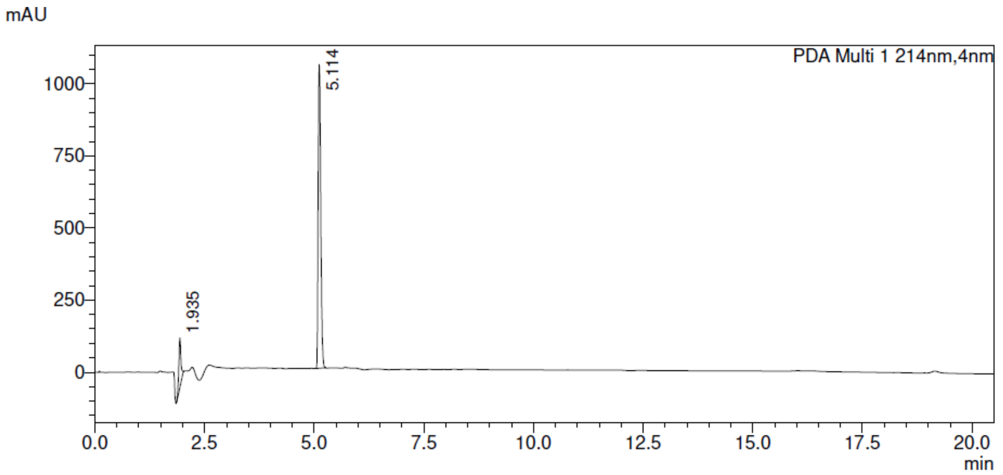

Figure S14. HPLC chromatogram of purified peptide (*N*-Me)HWG-NH_2 (Table S3, entry 4), (eluent A 0.1%TFA in H_2O, eluent B 0.1%TFA in 80% ACN, gradient 10-90%B in 20 min, flow rate = 1.0 mL/min, T= 30°C, λ = 214 nm) t_R = 5.114 min (major).

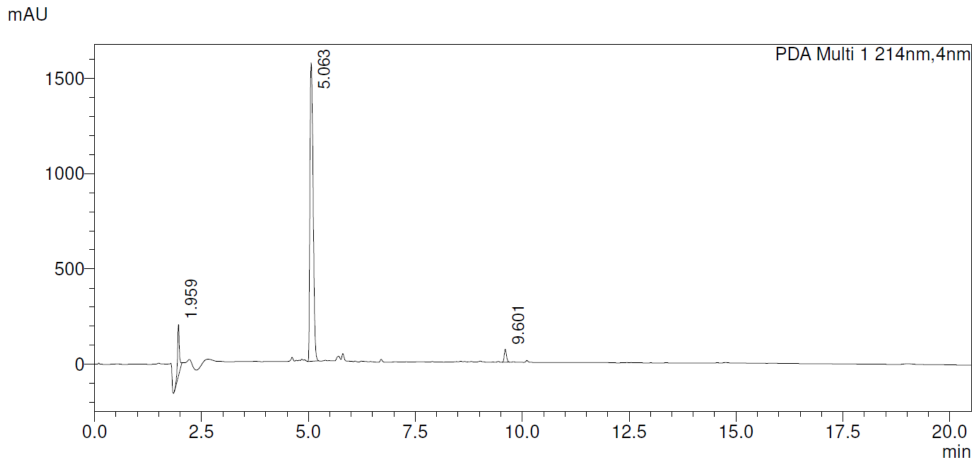

Figure S15. HPLC chromatogram of crude peptide (*N*-Me)HWG-NH_2 (Table S3, entry 5), , (eluent A 0.1%TFA in H_2O, eluent B 0.1%TFA in 80% ACN, gradient 10-90%B in 20 min, flow rate = 1.0 mL/min, T= 30°C, λ = 214 nm) t_R = 5.063 min (major), t_R = 9.601 min (minor).

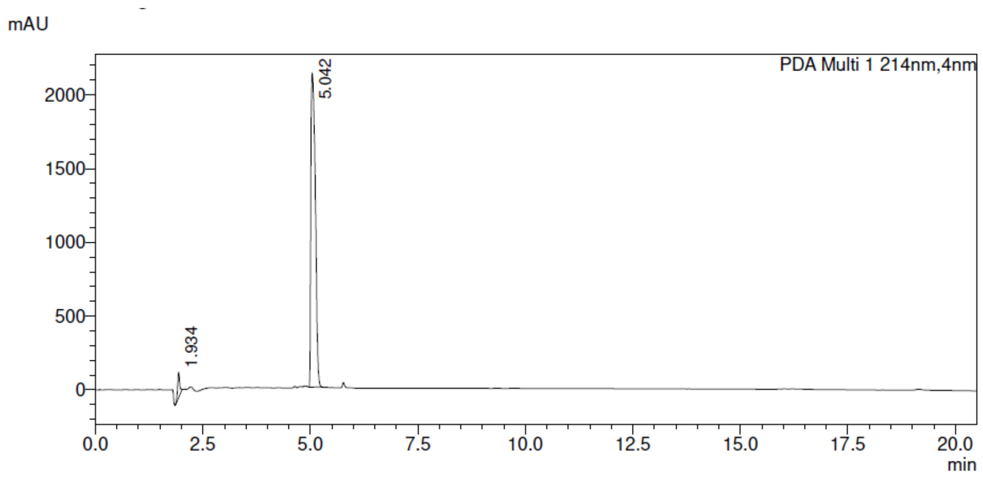

Figure S16. HPLC chromatogram of purified peptide (*N*-Me)HWG-NH_2 (Table S3, entry 5), (eluent A 0.1%TFA in H_2O, eluent B 0.1%TFA in 80% ACN, gradient 10-90%B in 20 min, flow rate = 1.0 mL/min, T= 30°C, λ = 214 nm) t_R = 5.042 min (major).

Me-SWG-NH$_2$

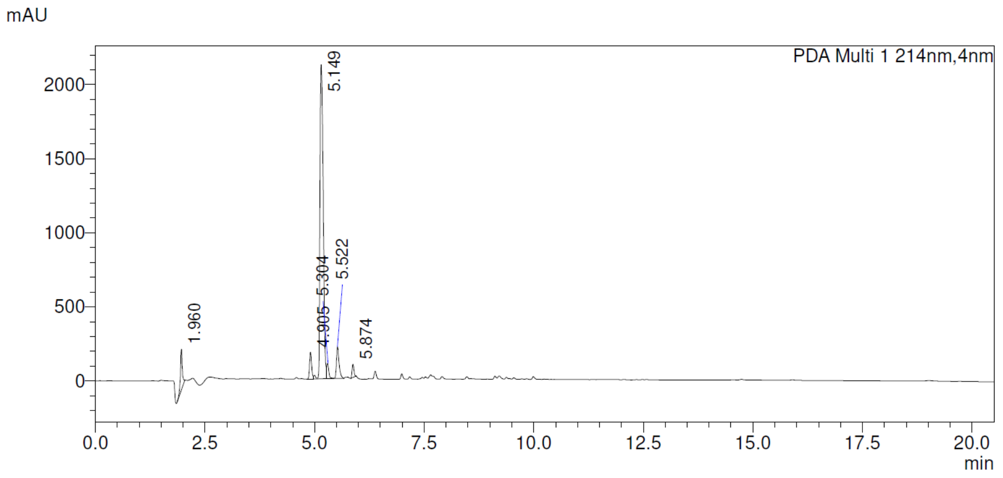

Figure S17. HPLC chromatogram of crude peptide (*N*-Me)SWG-NH$_2$ (Table S4, entry 3), (eluent A 0.1%TFA in H_2O, eluent B 0.1%TFA in 80% ACN, gradient 10-90%B in 20 min, flow rate = 1.0 mL/min, T= 30°C, λ = 214 nm) t_R = 5.149 min (major, (*N*-Me)SWG-NH$_2$), t_R = 4.905 min (minor), t_R = 5.304 min (minor), t_R = 5.522 min (minor), t_R = 5.874 min (minor).

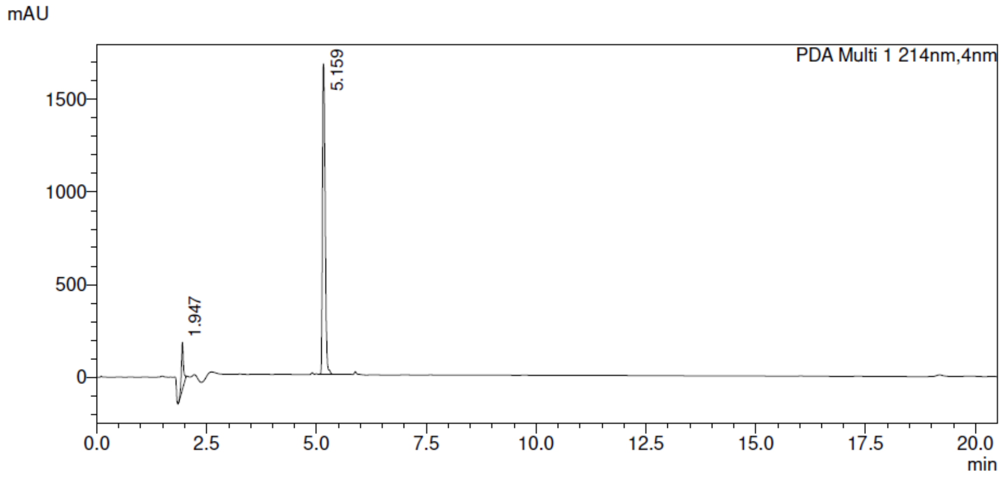

Figure S18. HPLC chromatogram of purified peptide (*N*-Me)SWG-NH$_2$ (Table S4, entry 3), (eluent A 0.1%TFA in H_2O, eluent B 0.1%TFA in 80% ACN, gradient 10-90%B in 20 min, flow rate = 1.0 mL/min, T= 30°C, λ = 214 nm) t_R = 5.159 min (major).

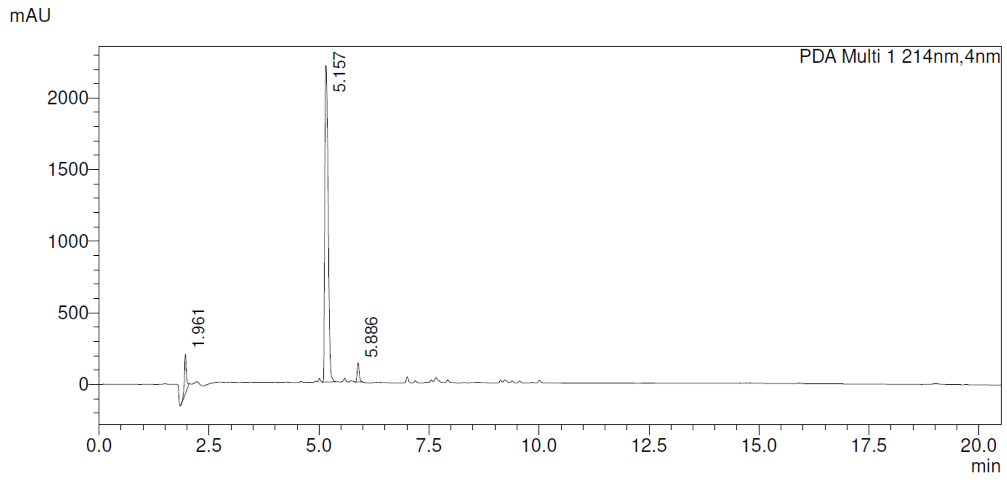

Figure S19. HPLC chromatogram of crude peptide (*N*-Me)SWG-NH_2 (Table S4, entry 4), (eluent A 0.1%TFA in H_2O, eluent B 0.1%TFA in 80% ACN, gradient 10-90%B in 20 min, flow rate = 1.0 mL/min, T= 30°C, λ = 214 nm) t_R = 5.157 min (major, (*N*-Me)SWG-NH_2), t_R = 5.886 min (minor).

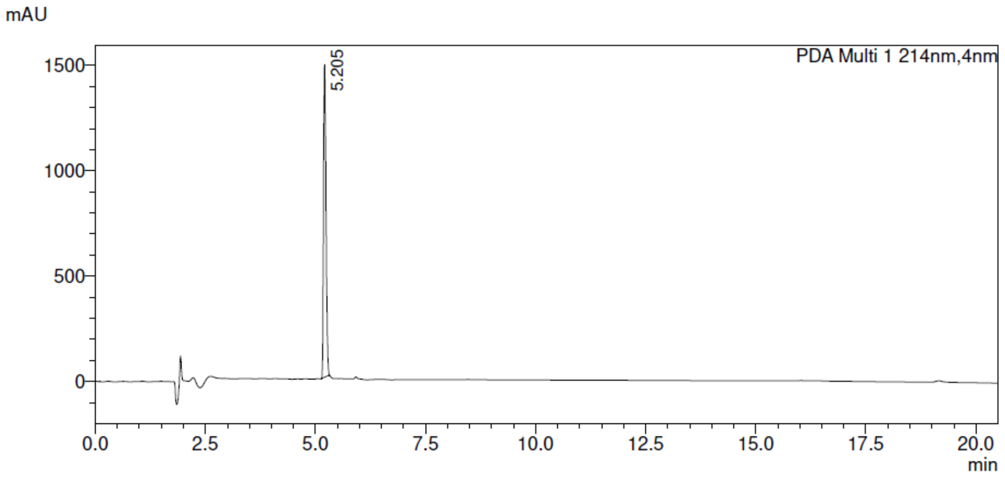

Figure S20. HPLC chromatogram of purified peptide (*N*-Me)SWG-NH_2 (Table S4, entry 4), (eluent A 0.1%TFA in H_2O, eluent B 0.1%TFA in 80% ACN, gradient 10-90%B in 20 min, flow rate = 1.0 mL/min, T= 30°C, λ = 214 nm) t_R = 5.205 min (major).

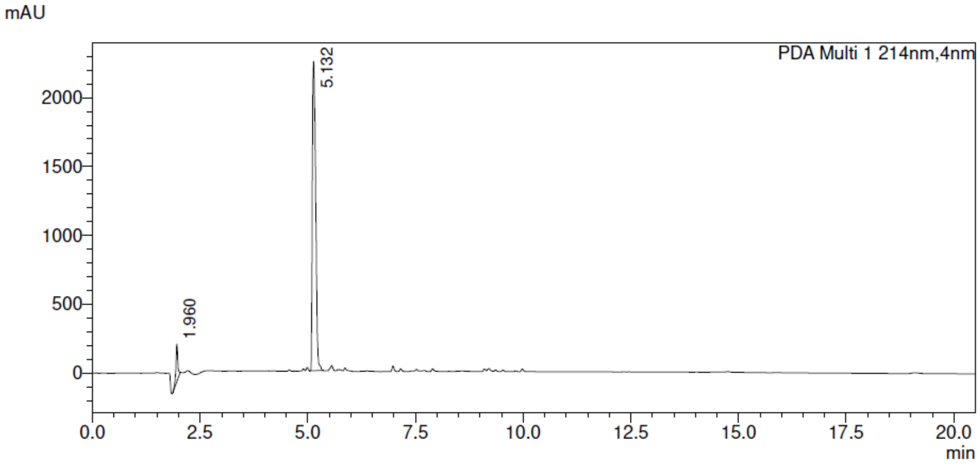

Figure S21. HPLC chromatogram of crude peptide (*N*-Me)SWG-NH_2 (Table S4, entry 5), (eluent A 0.1%TFA in H_2O, eluent B 0.1%TFA in 80% ACN, gradient 10-90%B in 20 min, flow rate = 1.0 mL/min, T= 30°C, λ = 214 nm) t_R = 5.132 min (major).

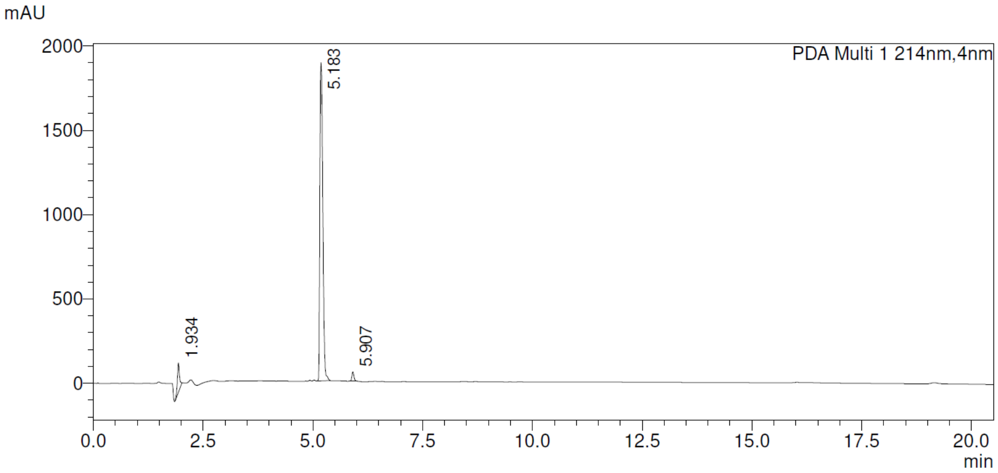

Figure S22. HPLC chromatogram of purified peptide (*N*-Me)SWG-NH_2 (Table S4, entry 5), (eluent A 0.1%TFA in H_2O, eluent B 0.1%TFA in 80% ACN, gradient 10-90%B in 20 min, flow rate = 1.0 mL/min, T= 30°C, λ = 214 nm) t_R = 5.183 min (major), t_R = 5.907 min (minor).

Me-WWG-NH₂

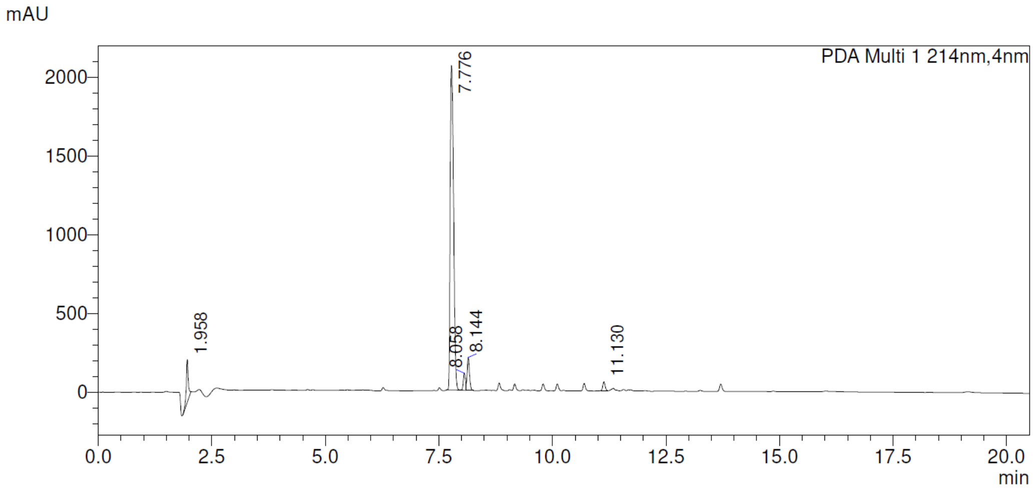

Figure S23. HPLC chromatogram of crude peptide (*N*-Me)WWG-NH_2 (Table S5, entry 3), (eluent A 0.1%TFA in H_2O, eluent B 0.1%TFA in 80% ACN, gradient 10-90%B in 20 min, flow rate = 1.0 mL/min, T= 30°C, λ = 214 nm) t_R = 7.776 min (major, (*N*-Me)WWG-NH_2), t_R = 8.058 min (minor), t_R = 8.144 min (minor), t_R = 11.130 min (minor).

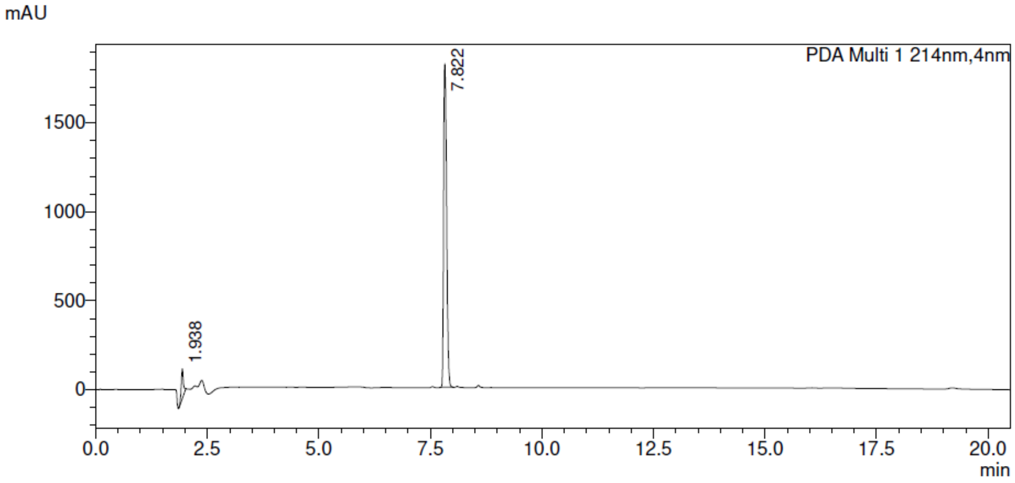

Figure S24. HPLC chromatogram of purified peptide (*N*-Me)WWG-NH_2 (Table S5, entry 3), (eluent A 0.1%TFA in H_2O, eluent B 0.1%TFA in 80% ACN, gradient 10-90%B in 20 min, flow rate = 1.0 mL/min, T= 30°C, λ = 214 nm) t_R = 7.822 min (major).

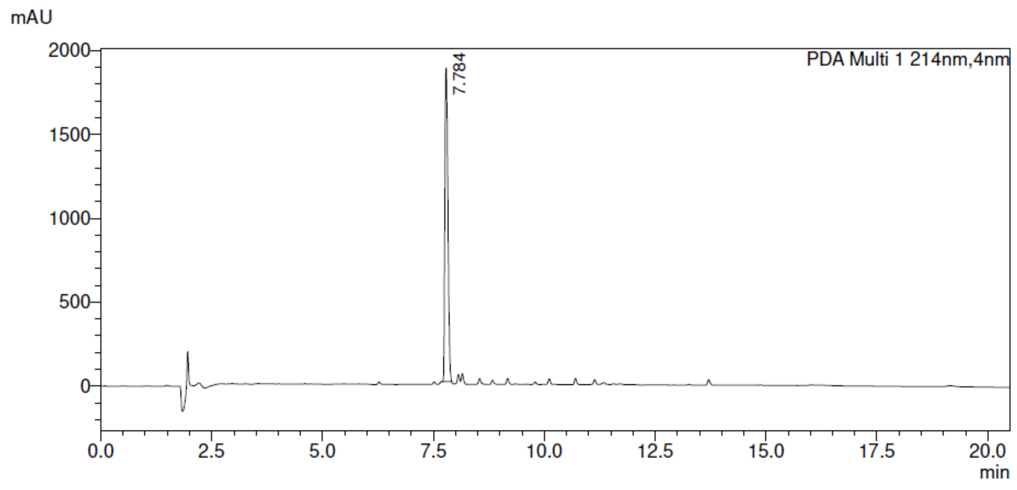

Figure S25. HPLC chromatogram of crude peptide (*N*-Me)WWG-NH_2 (Table S5, entry 4), (eluent A 0.1%TFA in H_2O, eluent B 0.1%TFA in 80% ACN, gradient 10-90%B in 20 min, flow rate = 1.0 mL/min, T= 30°C, λ = 214 nm) t_R = 7.784 min (major).

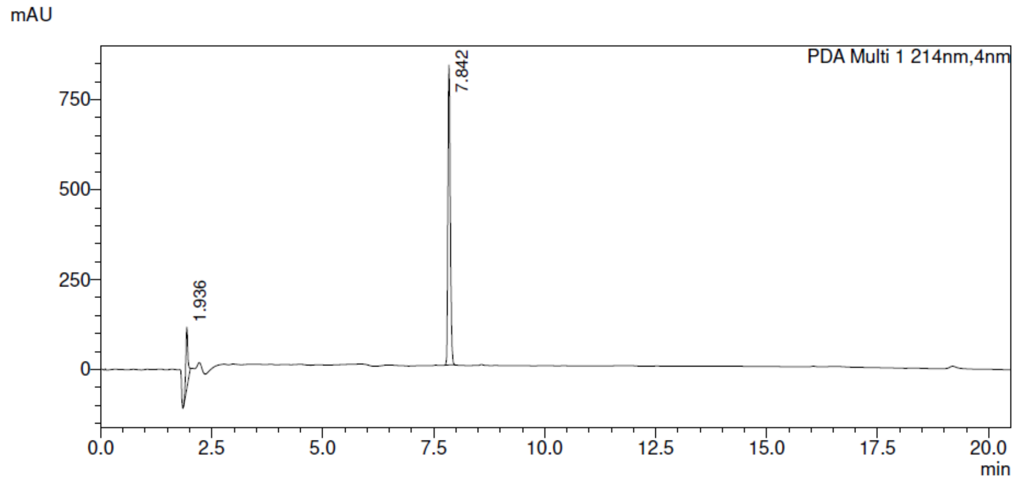

Figure S26. HPLC chromatogram of purified peptide (*N*-Me)WWG-NH_2 (Table S5, entry 4), (eluent A 0.1%TFA in H_2O, eluent B 0.1%TFA in 80% ACN, gradient 10-90%B in 20 min, flow rate = 1.0 mL/min, T= 30°C, λ = 214 nm) t_R = 7.842 min (major).

Me-YWG-NH$_2$

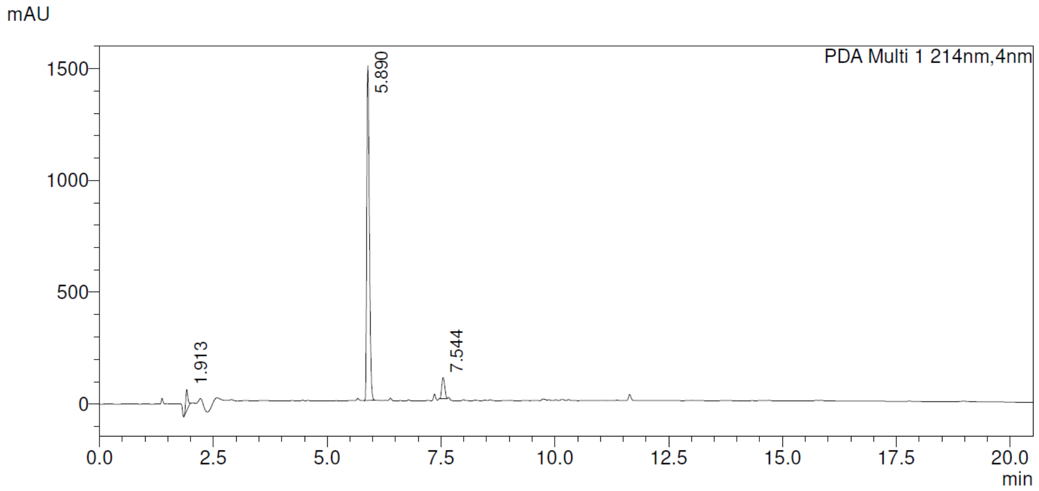

Figure S27. HPLC chromatogram of crude peptide (*N*-Me)YWG-NH$_2$ (Table S6, entry 3), (eluent A 0.1%TFA in H_2O, eluent B 0.1%TFA in 80% ACN, gradient 10-90%B in 20 min, flow rate = 1.0 mL/min, T= 30°C, λ = 214 nm) t_R = 5.890 min (major), t_R = 7.544 min (minor).

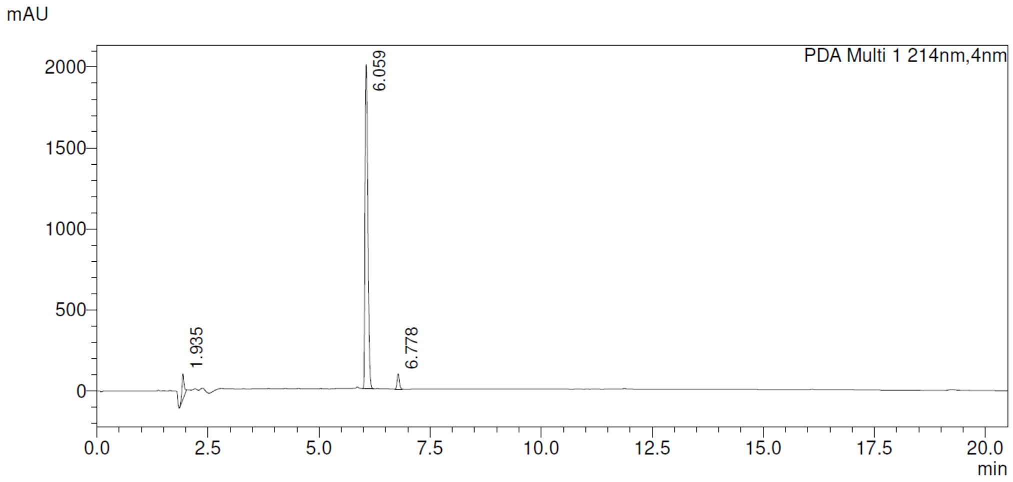

Figure S28. HPLC chromatogram of purified peptide (*N*-Me)YWG-NH$_2$ (Table S6, entry 3), (eluent A 0.1%TFA in H_2O, eluent B 0.1%TFA in 80% ACN, gradient 10-90%B in 20 min, flow rate = 1.0 mL/min, T= 30°C, λ = 214 nm) t_R = 6.059 min (major), t_R = 6.778 min (minor).

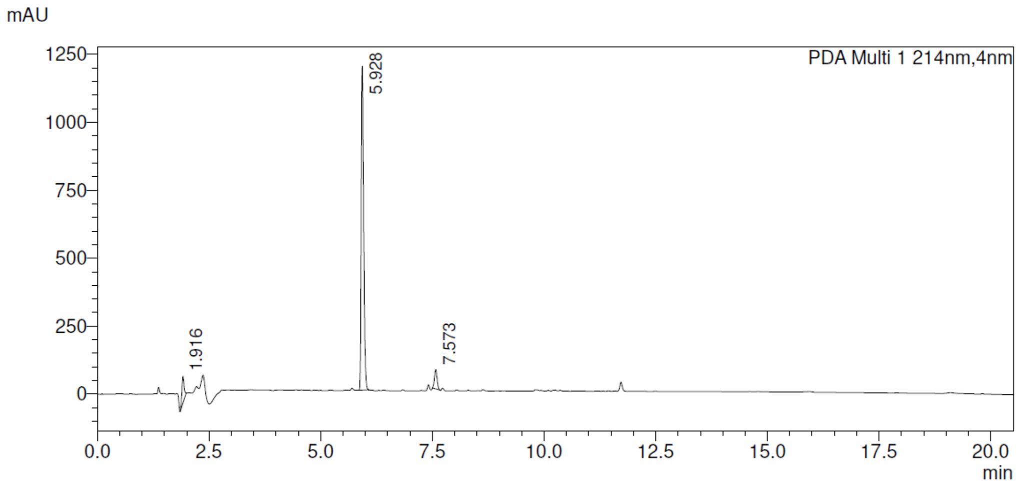

Figure S29. HPLC chromatogram of crude peptide (*N*-Me)YWG-NH_2 (Table S6, entry 4), (eluent A 0.1%TFA in H_2O, eluent B 0.1%TFA in 80% ACN, gradient 10-90%B in 20 min, flow rate = 1.0 mL/min, T= 30°C, λ = 214 nm) t_R = 5.928 min (major), t_R = 7.573 min (minor).

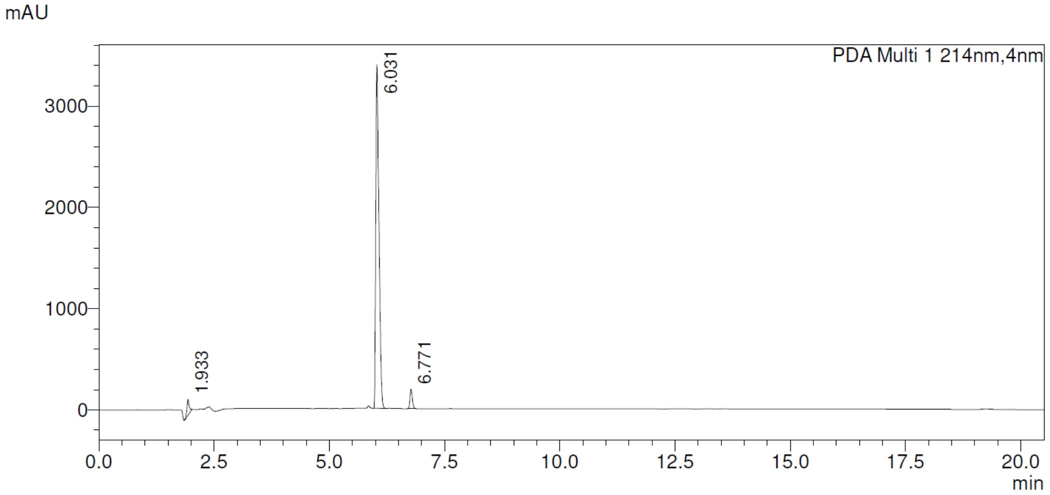

Figure S30. HPLC chromatogram of purified peptide (*N*-Me)YWG-NH_2 (Table S6, entry 4), (eluent A 0.1%TFA in H_2O, eluent B 0.1%TFA in 80% ACN, gradient 10-90%B in 20 min, flow rate = 1.0 mL/min, T= 30°C, λ = 214 nm) t_R = 6.031 min (major), t_R = 6.771 min (minor).

Me-DWG-NH_2

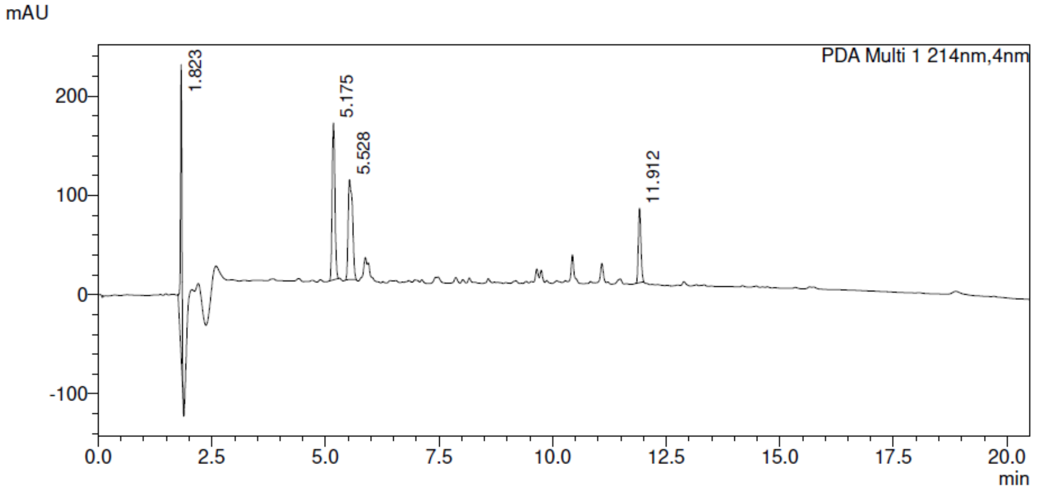

Figure S31. HPLC chromatogram of crude peptide (*N*-Me)DWG-NH_2 (Table S7, entry 1), (eluent A 0.1%TFA in H_2O, eluent B 0.1%TFA in 80% ACN, gradient 10-90%B in 20 min, flow rate = 1.0 mL/min, T= 30°C, λ = 214 nm) t_R = 5.175 min (major), t_R = 5.528 min ((*N*-Me)DWG-NH_2), t_R = 11.912 min (minor).

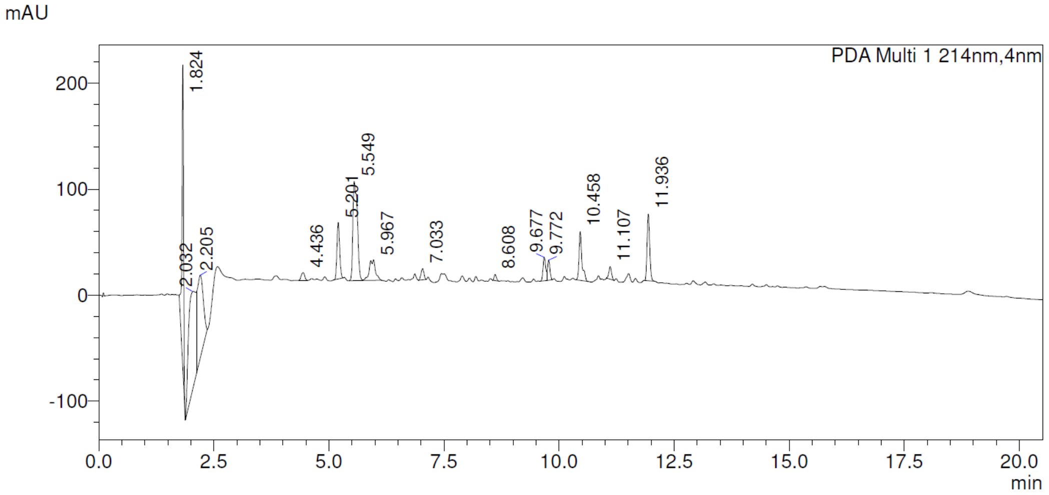

Figure S32. HPLC chromatogram of crude peptide (*N*-Me)DWG-NH_2 (Table S7, entry 2), (eluent A 0.1%TFA in H_2O, eluent B 0.1%TFA in 80% ACN, gradient 10-90%B in 20 min, flow rate = 1.0 mL/min, T= 30°C, λ = 214 nm), t_R = 4.436 min (minor), t_R = 5.201 min ((*N*-Me)DWG-NH_2), t_R = 5.549 min (major), t_R = 5.967 min (minor), t_R = 7.033 min (minor), t_R = 8.608 min (minor), t_R = 9.677 min (minor), t_R = 9.772 min (minor), t_R = 10.458 min (minor), t_R = 11.107 min (minor), t_R = 11.936 min (minor).

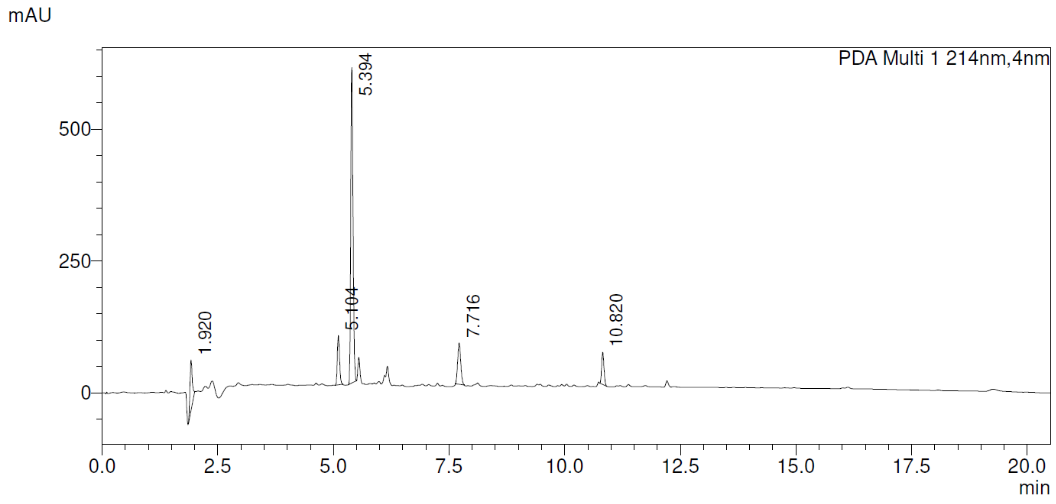

Figure S33. HPLC chromatogram of crude peptide (*N*-Me)DWG-NH_2 (Table S7, entry 4), (eluent A 0.1%TFA in H_2O, eluent B 0.1%TFA in 80% ACN, gradient 10-90%B in 20 min, flow rate = 1.0 mL/min, T= 30°C, λ = 214 nm) t_R = 5.394 min (major, (*N*-Me)DWG-NH_2), t_R = 5.104 min (minor), t_R = 7.716 min (minor), t_R = 10.820 min (minor).

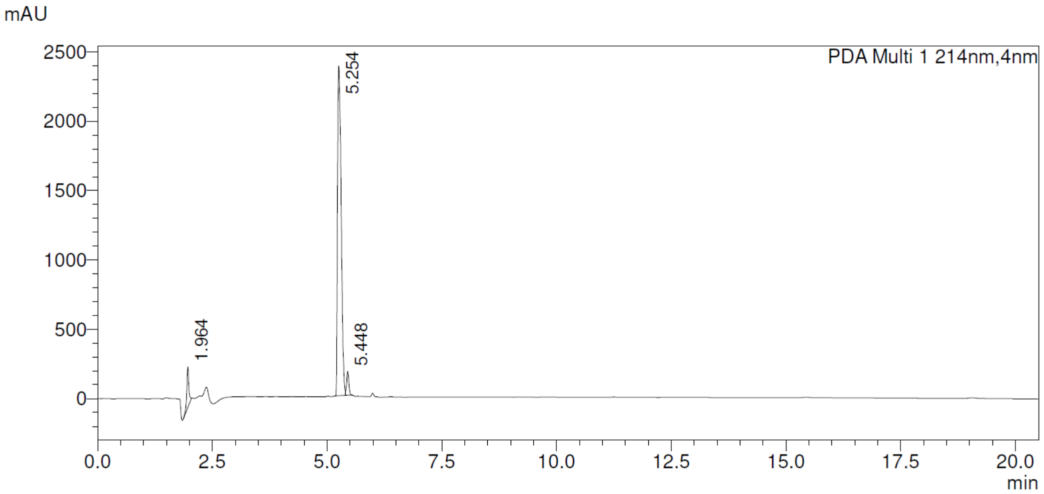

Figure S34. HPLC chromatogram of purified peptide (*N*-Me)DWG-NH_2 (Table S7, entry 4), (eluent A 0.1%TFA in H_2O, eluent B 0.1%TFA in 80% ACN, gradient 10-90%B in 20 min, flow rate = 1.0 mL/min, T= 30°C, λ = 214 nm) t_R = 5.254 min (major, (*N*-Me)DWG-NH_2), t_R = 5.448 min (minor).

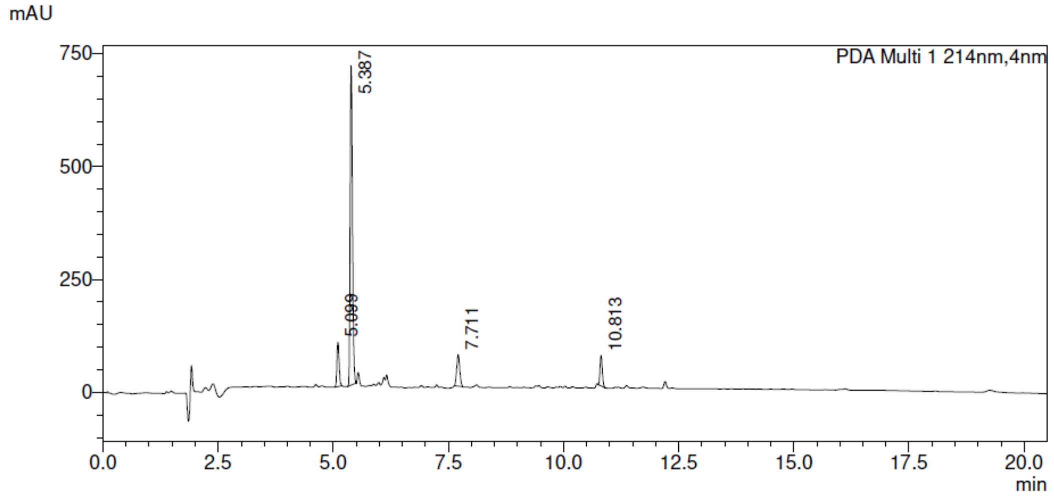

Figure S35. HPLC chromatogram of crude peptide (*N*-Me)DWG-NH_2 (Table S7, entry 5), (eluent A 0.1%TFA in H_2O, eluent B 0.1%TFA in 80% ACN, gradient 10-90%B in 20 min, flow rate = 1.0 mL/min, T= 30°C, λ = 214 nm) t_R = 5.387 min (major, (*N*-Me)DWG-NH_2), t_R = 5.099 min (minor), t_R = 7.711 (minor), t_R = 10.813 (minor).

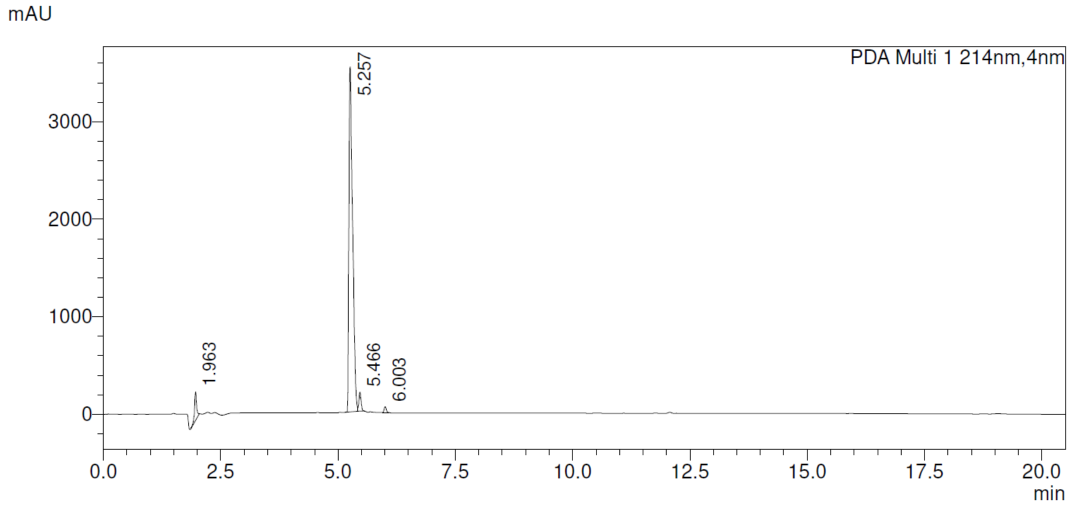

Figure S36. HPLC chromatogram of purified peptide (*N*-Me)DWG-NH_2 (Table S7, entry 5), (eluent A 0.1%TFA in H_2O, eluent B 0.1%TFA in 80% ACN, gradient 10-90%B in 20 min, flow rate = 1.0 mL/min, T= 30°C, λ = 214 nm) t_R = 5.257 min (major, (*N*-Me)DWG-NH_2), t_R = 5.466 min (minor), t_R = 6.003 min (minor).

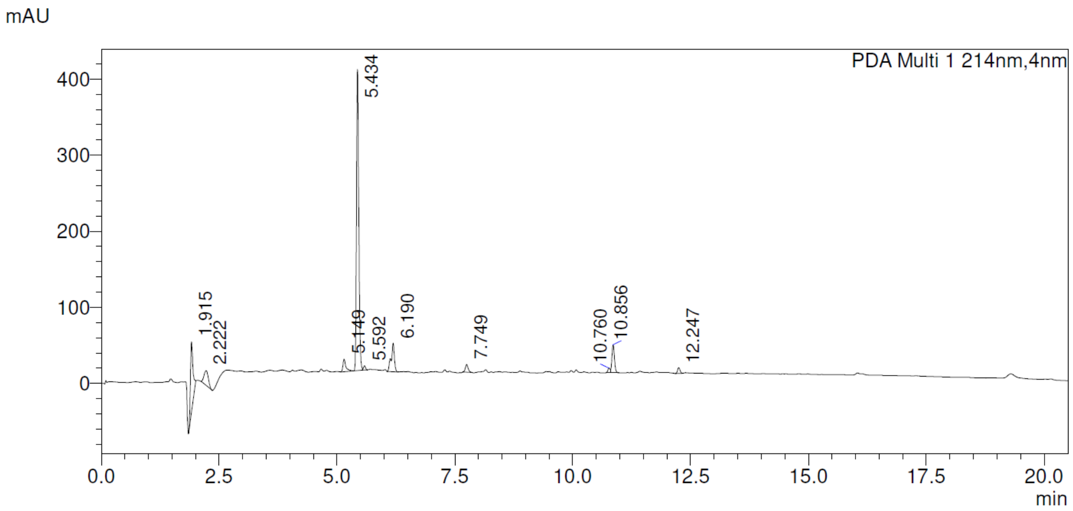

Figure S37. HPLC chromatogram of crude peptide (*N*-Me)DWG-NH_2 (Table S7, entry 6), (eluent A 0.1%TFA in H_2O, eluent B 0.1%TFA in 80% ACN, gradient 10-90%B in 20 min, flow rate = 1.0 mL/min, T= 30°C, λ = 214 nm) t_R = 5.434 min (major, (*N*-Me)DWG-NH_2), t_R = 5.149 min (minor), t_R = 5.592 min (minor), t_R = 6.190 min (minor), t_R = 7.749 min (minor), t_R = 10.760 min (minor), t_R = 10.856 min (minor), t_R = 12.247 min (minor).

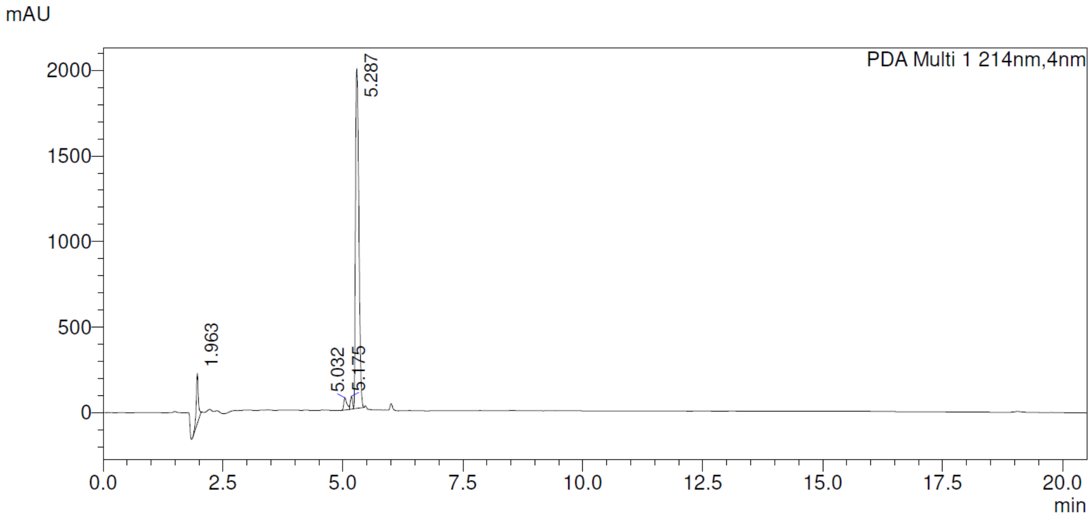

Figure S38. HPLC chromatogram of purified peptide (*N*-Me)DWG-NH_2 (Table S7, entry 6), (eluent A 0.1%TFA in H_2O, eluent B 0.1%TFA in 80% ACN, gradient 10-90%B in 20 min, flow rate = 1.0 mL/min, T= 30°C, λ = 214 nm) t_R = 5.287 min (major, (*N*-Me)DWG-NH_2), t_R = 5.032 min (minor), t_R = 5.175 min (minor).

Me-EWG-NH$_2$

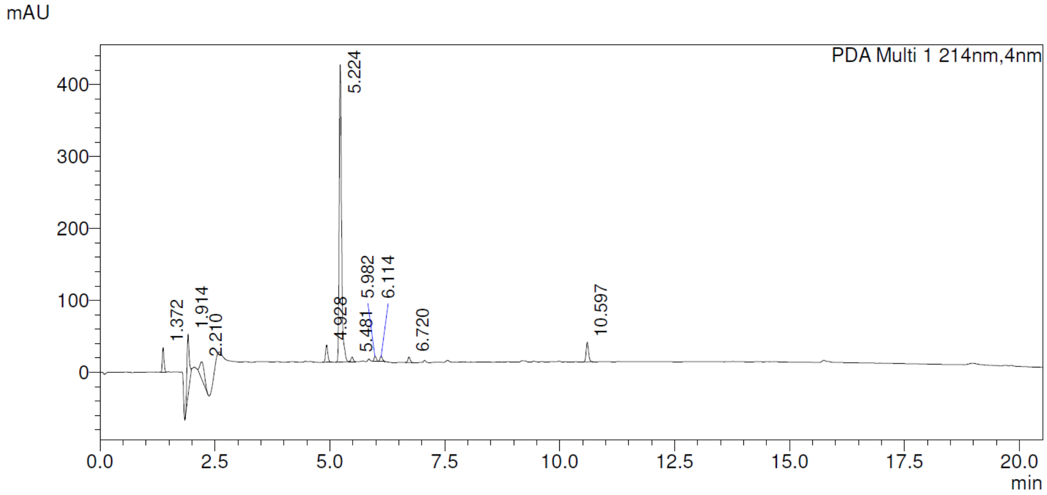

Figure S39. HPLC chromatogram of crude peptide (N-Me)EWG-NH$_2$ (Table S8, entry 2), (eluent A 0.1%TFA in H_2O, eluent B 0.1%TFA in 80% ACN, gradient 10-90%B in 20 min, flow rate = 1.0 mL/min, T= 30°C, λ = 214 nm) t_R = 5.224 min (major, (N-Me)EWG-NH$_2$), t_R = 4.928 min (minor), t_R = 5.481 min (minor), t_R = 5.982 min (minor), t_R = 6.114 min (minor), t_R = 6.720 min (minor), t_R = 10.597 min (minor).

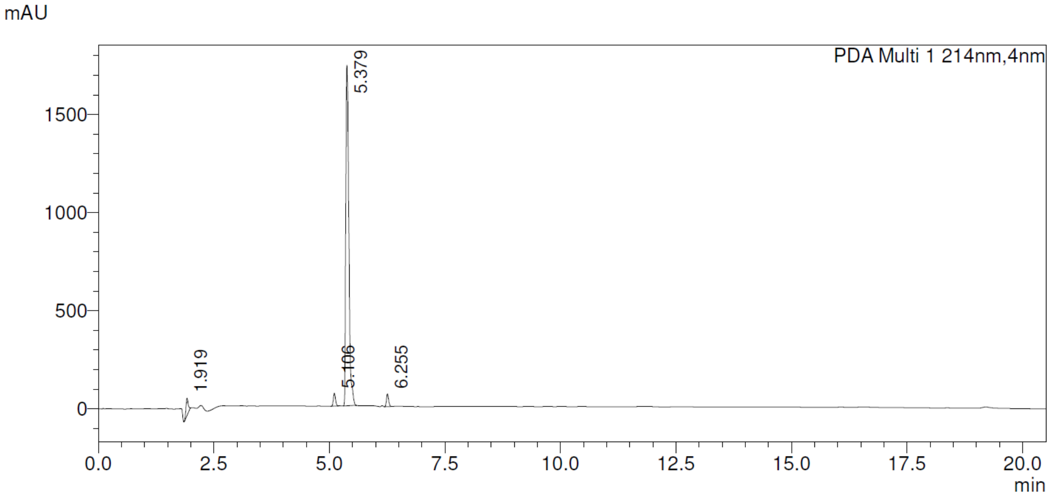

Figure S40. HPLC chromatogram of purified peptide (N-Me)EWG-NH$_2$ (Table S8, entry 2), (eluent A 0.1%TFA in H_2O, eluent B 0.1%TFA in 80% ACN, gradient 10-90%B in 20 min, flow rate = 1.0 mL/min, T= 30°C, λ = 214 nm) t_R = 5.379 min (major, (N-Me)EWG-NH$_2$), t_R = 5.106 min (minor), t_R = 6.255 min (minor).

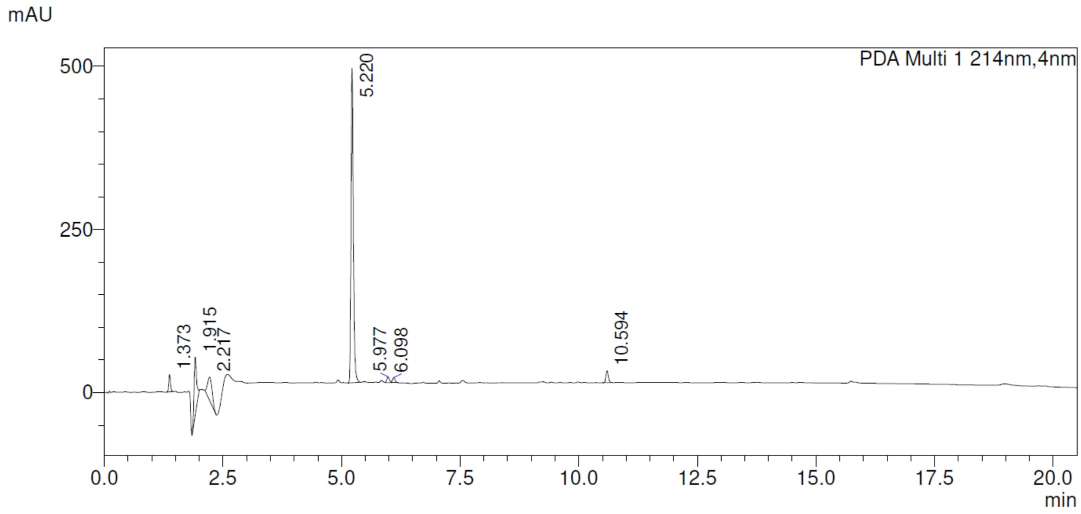

Figure S41. HPLC chromatogram of crude peptide (*N*-Me)EWG-NH_2 (Table S8, entry 3) (eluent A 0.1%TFA in H_2O, eluent B 0.1%TFA in 80% ACN, gradient 10-90%B in 20 min, flow rate = 1.0 mL/min, T= 30°C, λ = 214 nm) t_R = 5.220 min (major, (*N*-Me)EWG-NH_2), t_R = 5.977 min (minor), t_R = 6.098 min (minor), t_R = 10.594 min (minor).

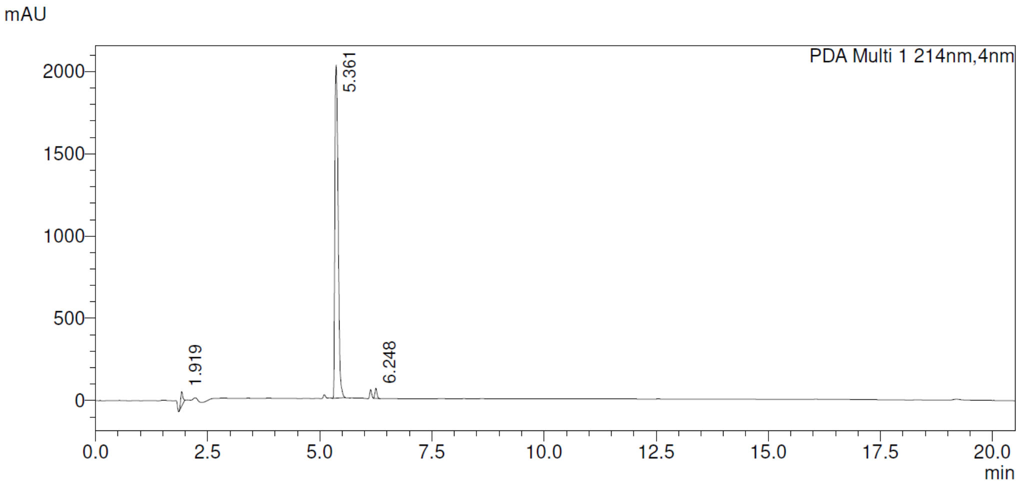

Figure S42. HPLC chromatogram of purified peptide (*N*-Me)EWG-NH_2 (Table S8, entry 3) (eluent A 0.1%TFA in H_2O, eluent B 0.1%TFA in 80% ACN, gradient 10-90%B in 20 min, flow rate = 1.0 mL/min, T= 30°C, λ = 214 nm) t_R = 5.361 min (major, (*N*-Me)EWG-NH_2), t_R = 6.248 min (minor).

Me-CWG-NH$_2$

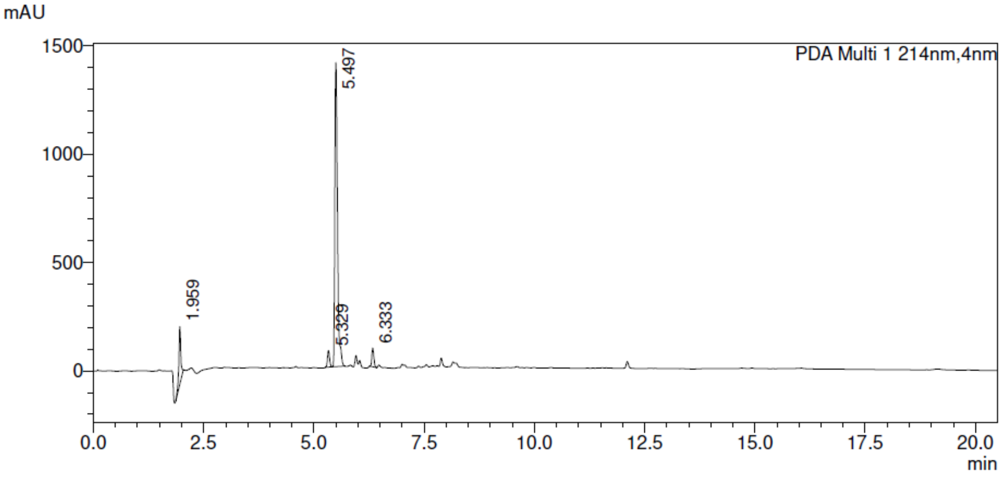

Figure S43. HPLC chromatogram of crude peptide (*N*-Me)CWG-NH$_2$ (Table S9, entry 3), (eluent A 0.1%TFA in H_2O, eluent B 0.1%TFA in 80% ACN, gradient 10-90%B in 20 min, flow rate = 1.0 mL/min, T= 30°C, λ = 214 nm) t_R = 5.497 min (major, (*N*-Me)CWG-NH$_2$), t_R = 5.329 min (minor), t_R = 6.333 min (minor).

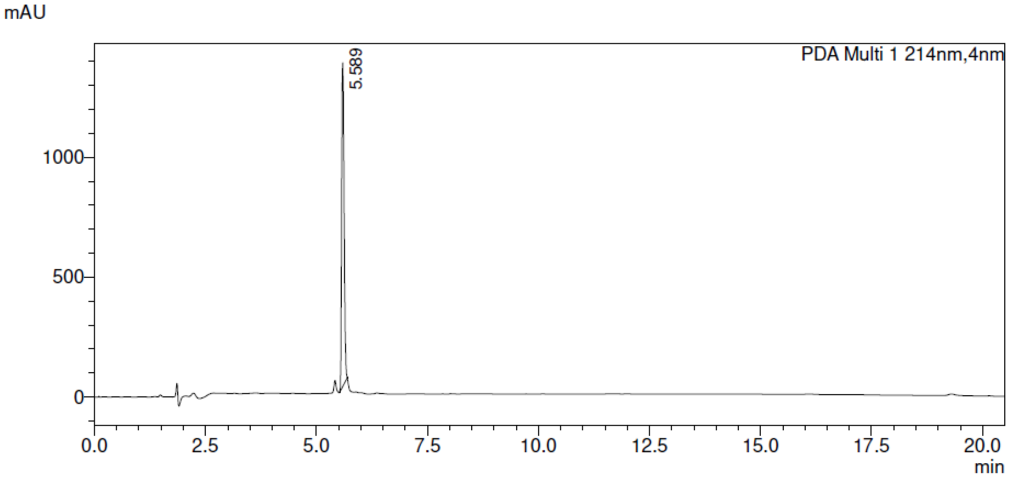

Figure S44. HPLC chromatogram of purified peptide (*N*-Me)CWG-NH$_2$ (Table S9, entry 3) (eluent A 0.1%TFA in H_2O, eluent B 0.1%TFA in 80% ACN, gradient 10-90%B in 20 min, flow rate = 1.0 mL/min, T= 30°C, λ = 214 nm) t_R = 5.589 min (major, (*N*-Me)CWG-NH$_2$), t_R = 5.417 min (minor).

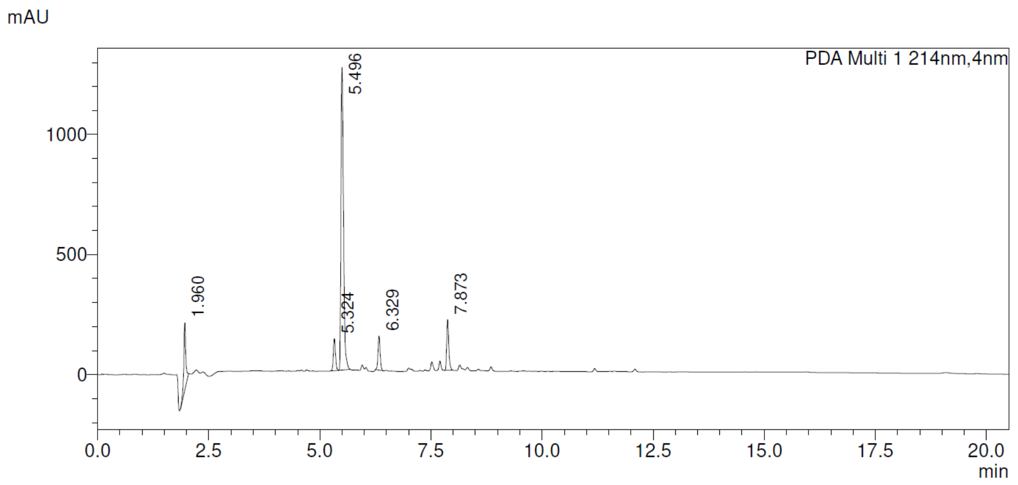

Figure S45. HPLC chromatogram of crude peptide (*N*-Me)CWG-NH_2 (Table S9, entry 4), (eluent A 0.1%TFA in H_2O, eluent B 0.1%TFA in 80% ACN, gradient 10-90%B in 20 min, flow rate = 1.0 mL/min, T= 30°C, λ = 214 nm) t_R = 5.496 min (major, (*N*-Me)CWG-NH_2), t_R = 5.324 min (minor), t_R = 5.324 min (minor), t_R = 6.329 min (minor), t_R = 7.873 min (minor).

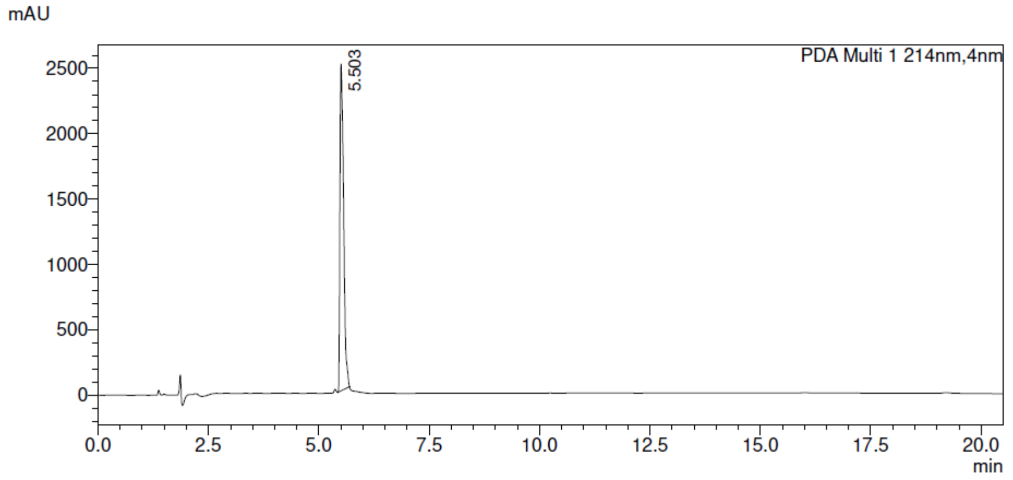

Figure S46. HPLC chromatogram of purified peptide (*N*-Me)CWG-NH_2 (Table S9, entry 4), (eluent A 0.1%TFA in H_2O, eluent B 0.1%TFA in 80% ACN, gradient 10-90%B in 20 min, flow rate = 1.0 mL/min, T= 30°C, λ = 214 nm) t_R = 5.503 min (major, (*N*-Me)CWG-NH_2).

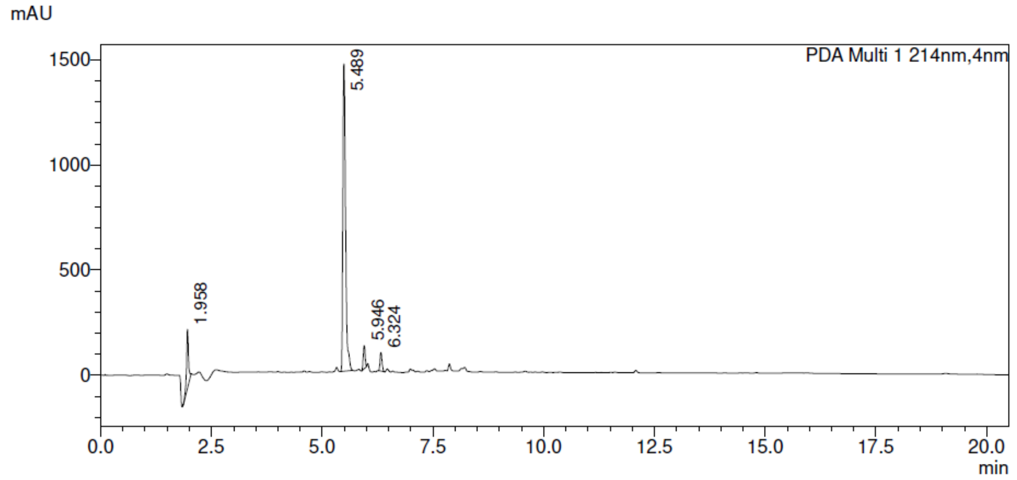

Figure S47. HPLC chromatogram of crude peptide (*N*-Me)CWG-NH_2 (Table S9, entry 5) (eluent A 0.1%TFA in H_2O, eluent B 0.1%TFA in 80% ACN, gradient 10-90%B in 20 min, flow rate = 1.0 mL/min, T= 30°C, λ = 214 nm) t_R = 5.489 min (major, (*N*-Me)CWG-NH_2), t_R = 5.946 min (minor), t_R = 6.324 min (minor).

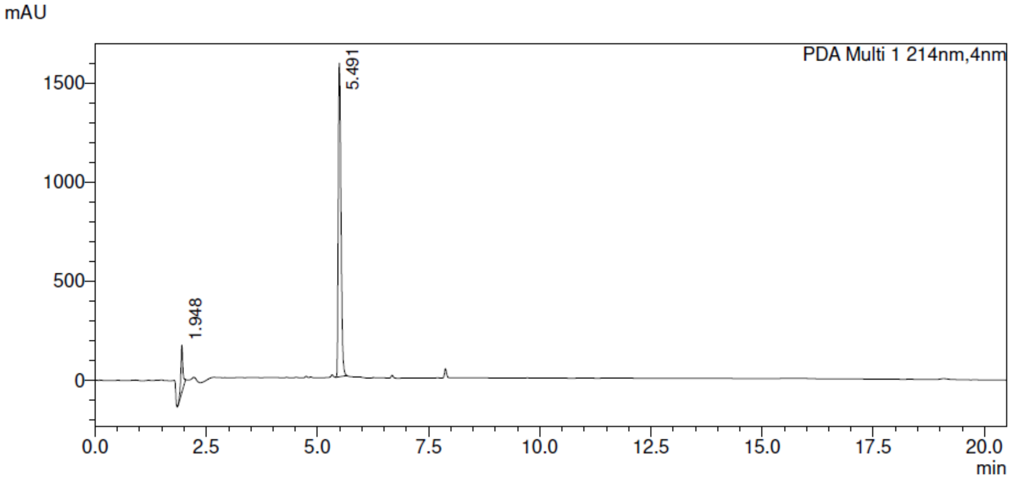

Figure S48. HPLC chromatogram of purified peptide (*N*-Me)CWG-NH_2 (Table S9, entry 5), (eluent A 0.1%TFA in H_2O, eluent B 0.1%TFA in 80% ACN, gradient 10-90%B in 20 min, flow rate = 1.0 mL/min, T= 30°C, λ = 214 nm) t_R = 5.491 min (major, (*N*-Me)CWG-NH_2).

Me-FWG-NH2

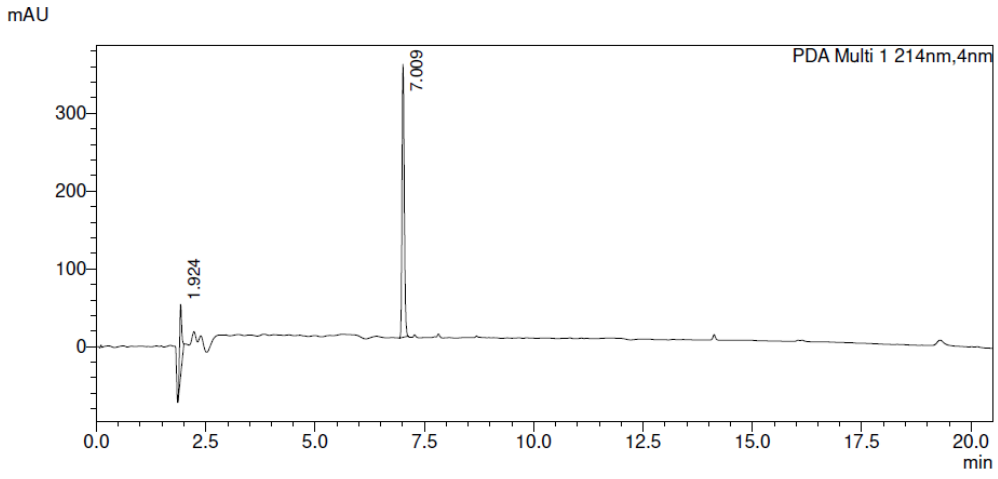

Figure S49. HPLC chromatogram of crude peptide (*N*-Me)FWG-NH2 (Table S10, entry 2), (eluent A 0.1%TFA in H_2O, eluent B 0.1%TFA in 80% ACN, gradient 10-90%B in 20 min, flow rate = 1.0 mL/min, T= 30°C, λ = 214 nm) t_R = 7.009 min (major, (*N*-Me)FWG-NH2).

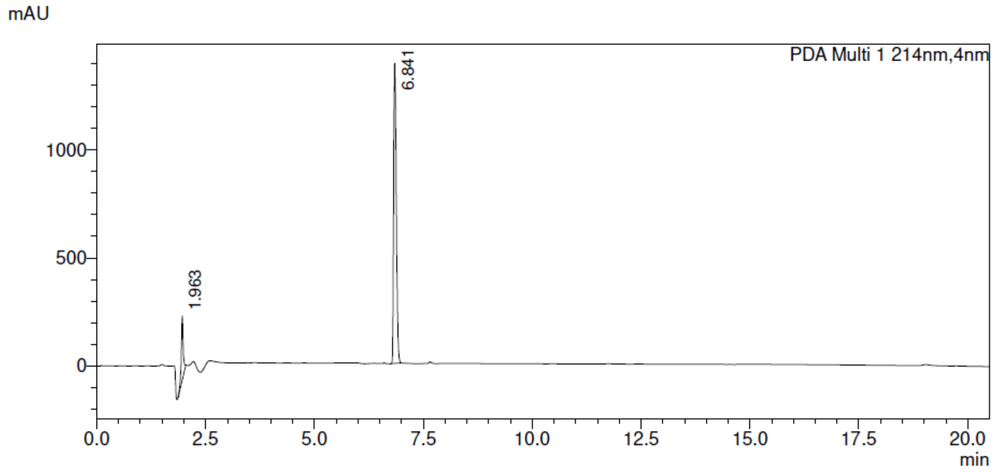

Figure S50. HPLC chromatogram of purifide peptide (*N*-Me)FWG-NH2 (Table S10, entry 2), (eluent A 0.1%TFA in H_2O, eluent B 0.1%TFA in 80% ACN, gradient 10-90%B in 20 min, flow rate = 1.0 mL/min, T= 30°C, λ = 214 nm) t_R = 6.541 min (major, (*N*-Me)FWG-NH2).

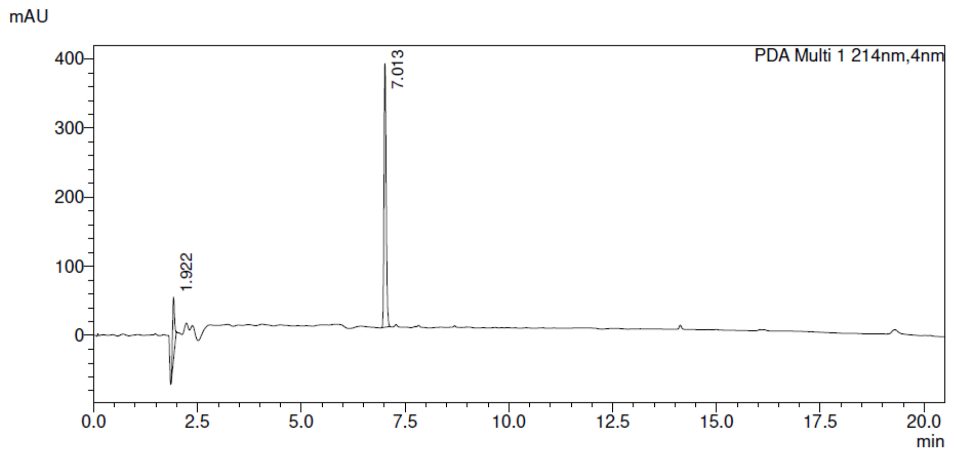

Figure S51. HPLC chromatogram of crude peptide (*N*-Me)FWG-NH_2 (Table S10, entry 3), (eluent A 0.1%TFA in H_2O, eluent B 0.1%TFA in 80% ACN, gradient 10-90%B in 20 min, flow rate = 1.0 mL/min, T= 30°C, λ = 214 nm) t_R = 7.013 min (major, (*N*-Me)FWG-NH_2).

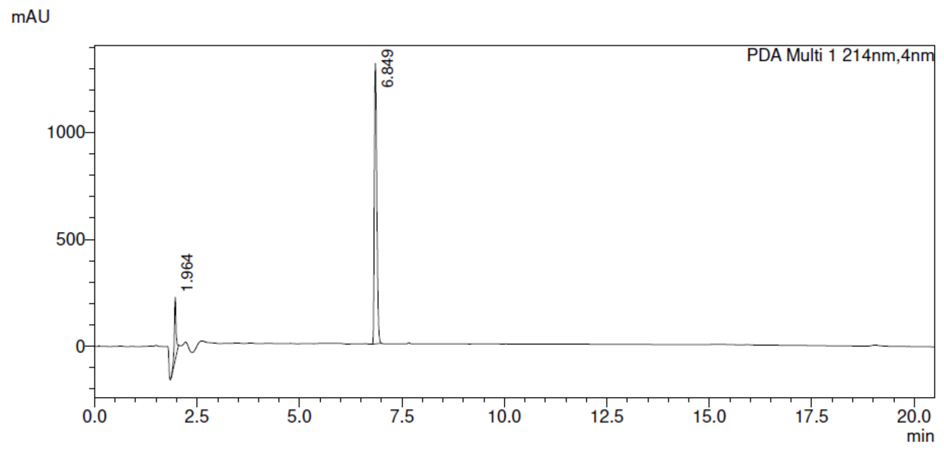

Figure S52. HPLC chromatogram of purified peptide (*N*-Me)FWG-NH_2 (Table S10, entry 3), (eluent A 0.1%TFA in H_2O, eluent B 0.1%TFA in 80% ACN, gradient 10-90%B in 20 min, flow rate = 1.0 mL/min, T= 30°C, λ = 214 nm) t_R = 6.849 min (major, (*N*-Me)FWG-NH_2).

Me-AWG-NH₂

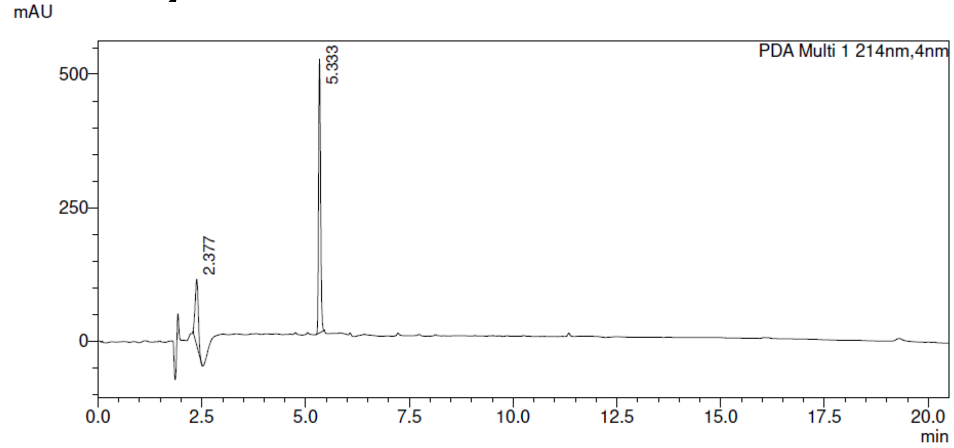

Figure S53. HPLC chromatogram of crude peptide (*N*-Me)AWG-NH_2 (Table S11, entry 2), (eluent A 0.1%TFA in H_2O, eluent B 0.1%TFA in 80% ACN, gradient 10-90%B in 20 min, flow rate = 1.0 mL/min, T= 30°C, λ = 214 nm) t_R = 5.333 min (major, (*N*-Me)AWG-NH_2).

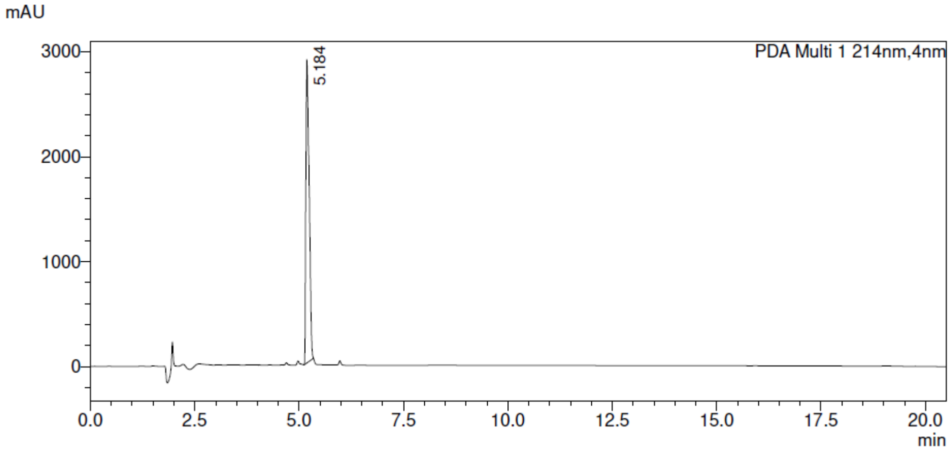

Figure S54. HPLC chromatogram of purified peptide (*N*-Me)AWG-NH_2 (Table S11, entry 2), (eluent A 0.1%TFA in H_2O, eluent B 0.1%TFA in 80% ACN, gradient 10-90%B in 20 min, flow rate = 1.0 mL/min, T= 30°C, λ = 214 nm) t_R = 5.184 min (major, (*N*-Me)AWG-NH_2).

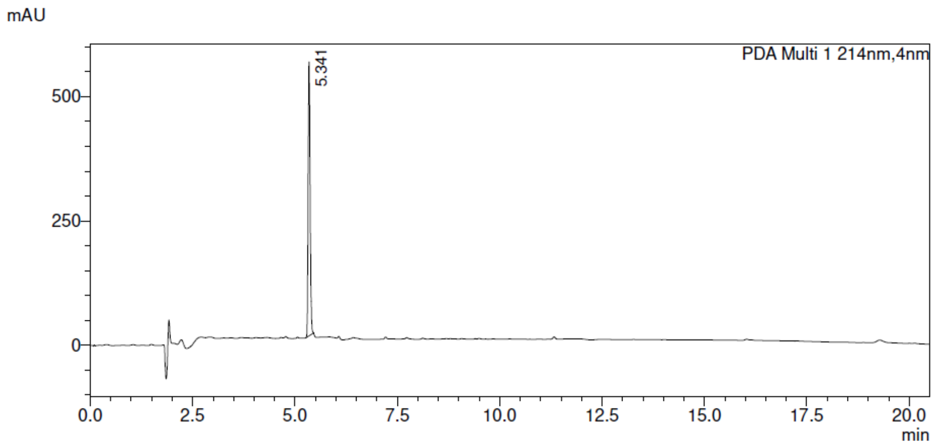

Figure S55. HPLC chromatogram of crude peptide (*N*-Me)AWG-NH_2 (Table S11, entry 3), (eluent A 0.1%TFA in H_2O, eluent B 0.1%TFA in 80% ACN, gradient 10-90%B in 20 min, flow rate = 1.0 mL/min, T= 30°C, λ = 214 nm) t_R = 5.341 min (major, (*N*-Me)AWG-NH_2).

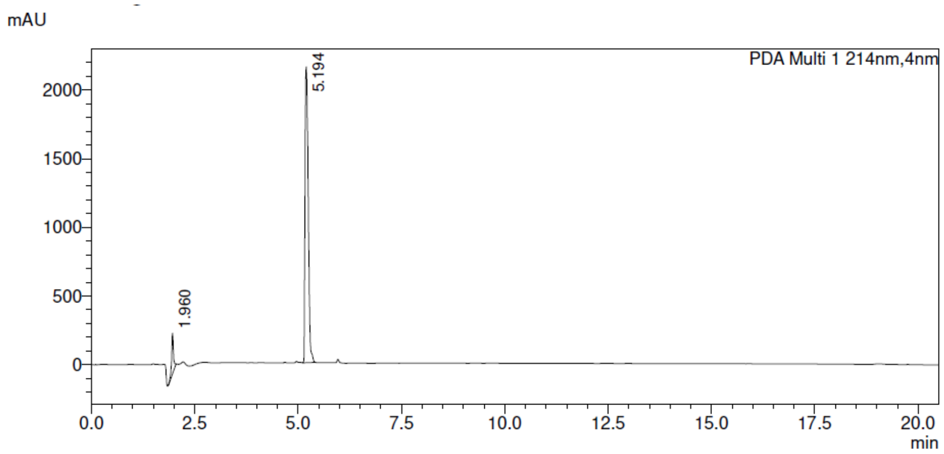

Figure S56. HPLC chromatogram of purified peptide (*N*-Me)AWG-NH_2 (Table S11, entry 3), (eluent A 0.1%TFA in H_2O, eluent B 0.1%TFA in 80% ACN, gradient 10-90%B in 20 min, flow rate = 1.0 mL/min, T= 30°C, λ = 214 nm) t_R = 5.194 min (major, (*N*-Me)AWG-NH_2).

MS analyses of synthesized peptides

MALDI MS (Autoflex maX MALDI-TOF spectrometer, Bruker Daltonics, Germany) using 2,5-dihydroxybenzoic acid matrix (DHB)

Table S12. Calculated and found masses of obtained peptides

Peptide	Calculated $[M+H]^+$	Found $[M+H]^+$
(*N*-Me)RWG-NH_2	431.247	431.241
(*N*-Me)RWG-OH	432.237	432.224
(*N*-Me)HWG-NH_2	412.207	412.193
(*N*-Me)SWG-NH_2	362.187	362.180
(*N*-Me)WWG-NH_2	461.227	461.224
(*N*-Me)YWG-NH_2	438.217	438.207
(*N*-Me)DWG-NH_2	390.177	390,167
(*N*-Me)EWG-NH_2	404.197	404.152
(*N*-Me)CWG-NH_2	378.157	378.153
(*N*-Me)FWG-NH_2	422.217	422.220
(*N*-Me)AWG-NH_2	346.187	346.180
1-SW1	955.517	955.393

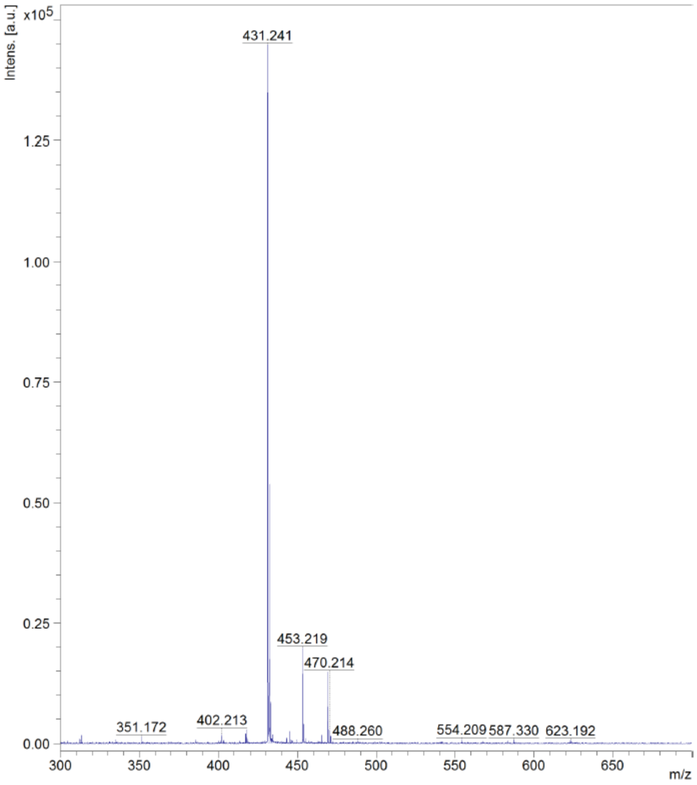

Figure S57. Mass analysis of peptide (*N*-Me)RWG-NH_2. MS (MALDI-TOF) m/z: Calculated $[M+H]^+$ 431.247; Found $[M+H]^+$ 431.241

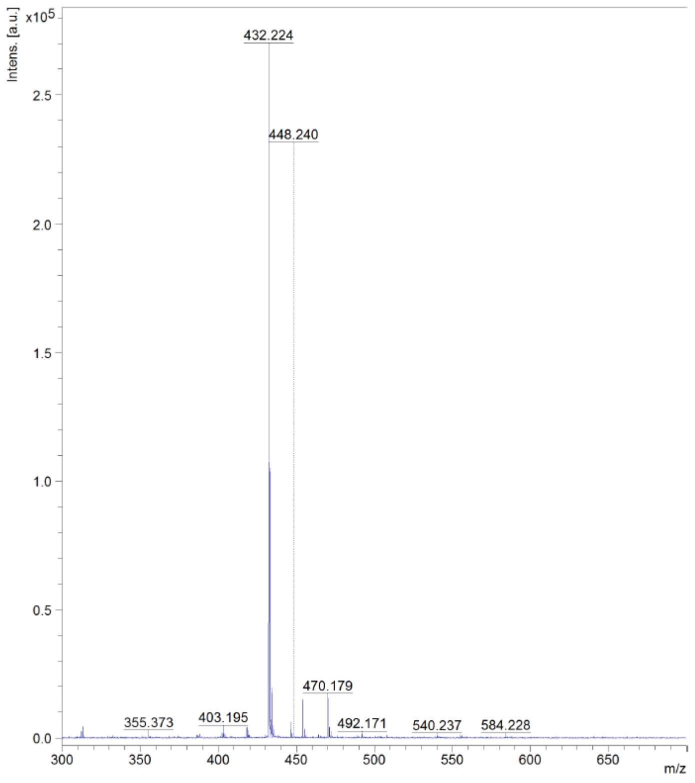

Figure S58. Mass analysis of peptide (*N*-Me)RWG-OH; MS (MALDI-TOF) m/z: Calculated $[M+H]^+$ 432.237; Found $[M+H]^+$ 432.22

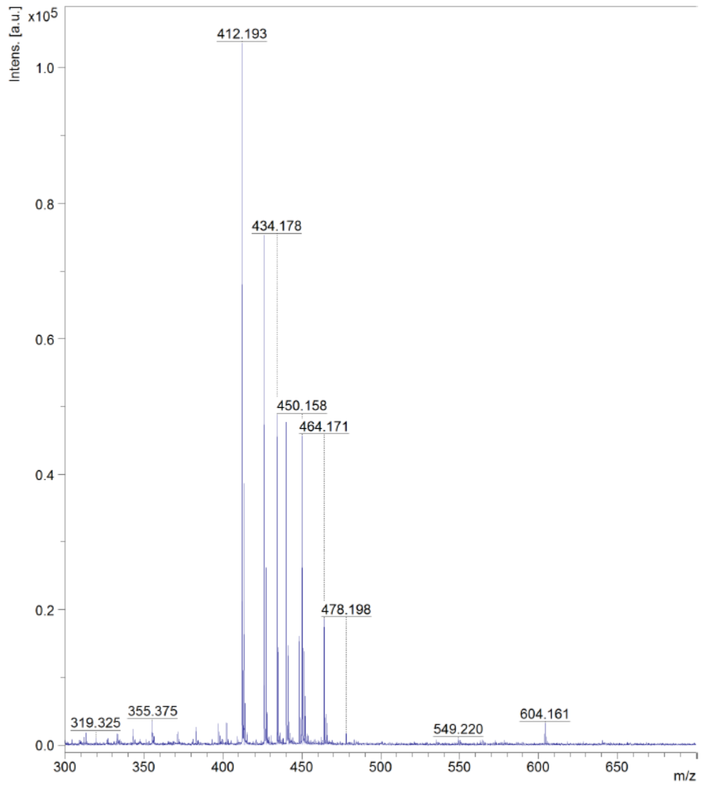

Figure S59. Mass analysis of peptide (*N*-Me)HWG-NH_2; MS (MALDI-TOF) m/z: Calculated $[M+H]^+$ 412.207; Found $[M+H]^+$ 412.19

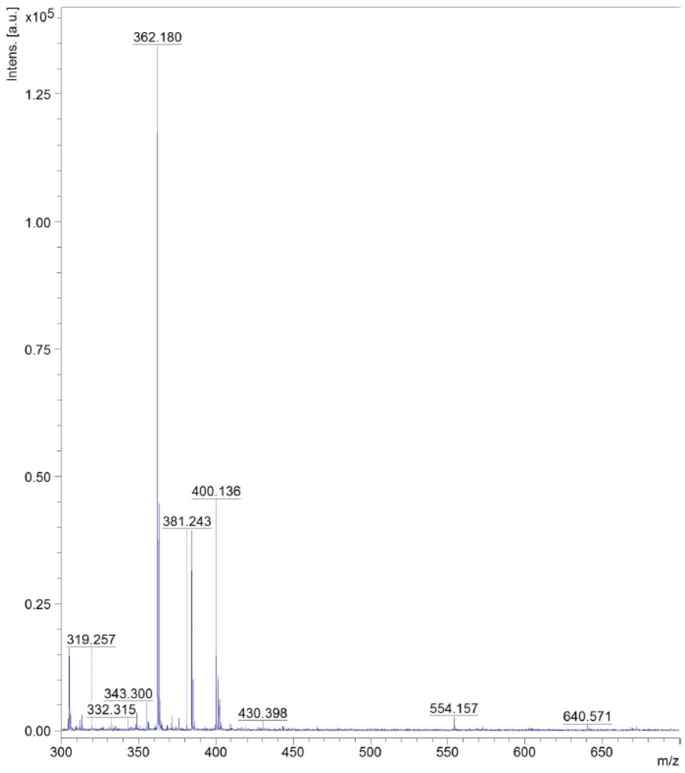

Figure S60. Mass analysis of peptide (*N*-Me)SWG-NH_2; MS (MALDI-TOF) m/z: Calculated $[M+H]^+$ 362.187; Found $[M+H]^+$ 362.18

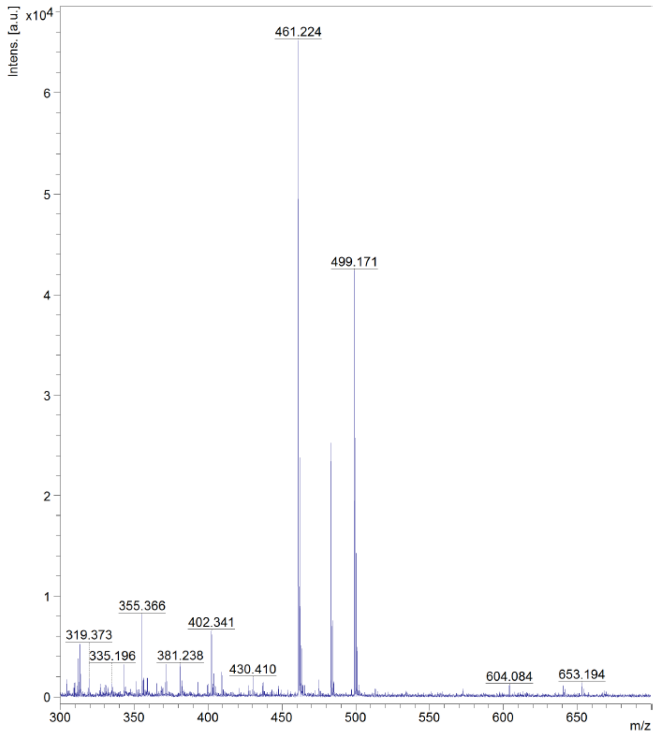

Figure S61. Mass analysis of peptide (*N*-Me)WWG-NH_2; MS (MALDI-TOF) m/z: Calculated $[M+H]^+$ 461.227; Found $[M+H]^+$ 461.22

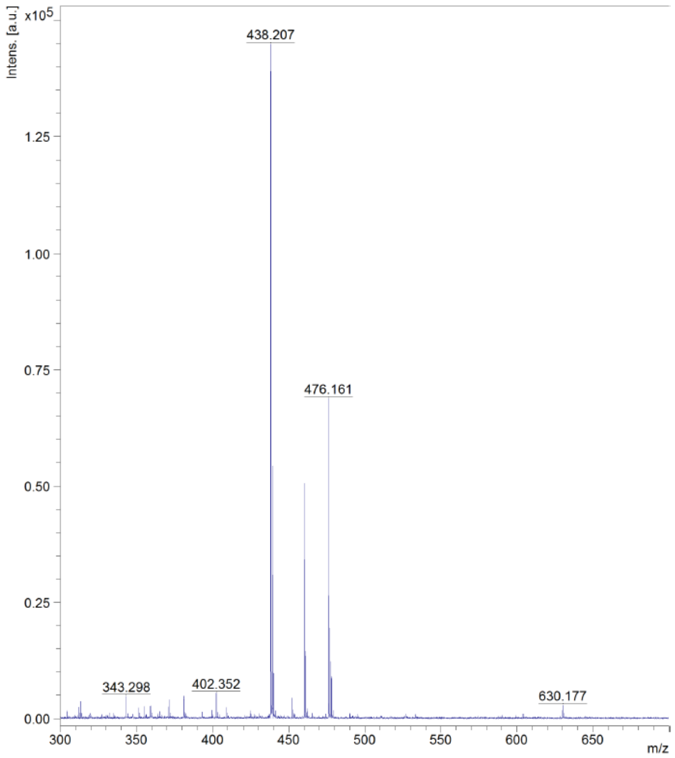

Figure S62. Mass analysis of peptide (*N*-Me)YWG-NH_2; MS (MALDI-TOF) m/z: Calculated $[M+H]^+$ 438.217; Found $[M+H]^+$ 438.21

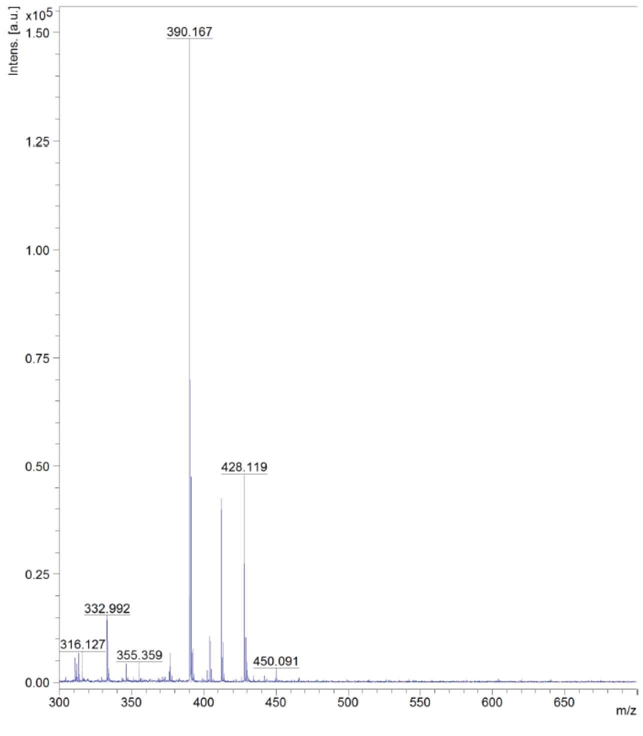

Figure S63. Mass analysis of peptide (*N*-Me)DWG-NH_2; MS (MALDI-TOF) m/z: Calculated $[M+H]^+$ 390.177; Found $[M+H]^+$ 390.17

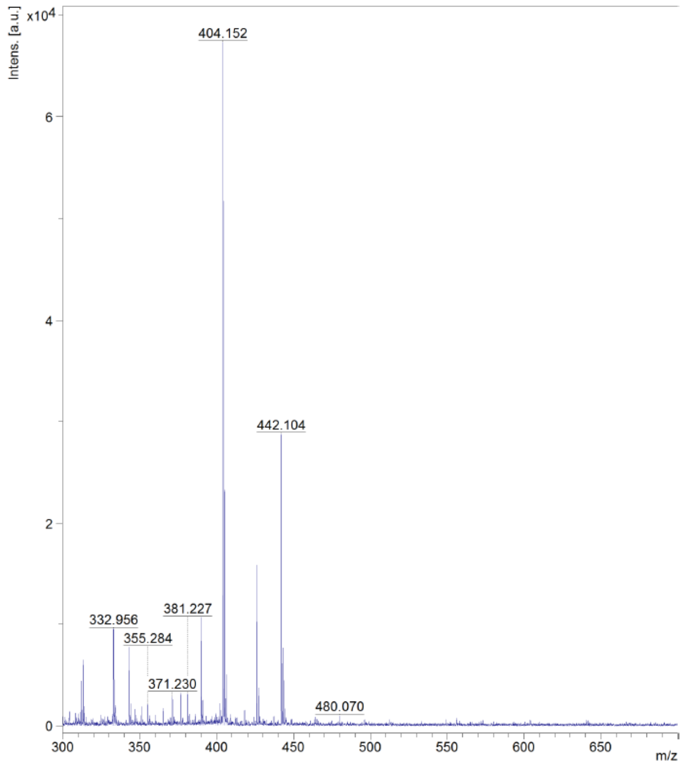

Figure S64. Mass analysis of peptide (*N*-Me)EWG-NH_2; MS (MALDI-TOF) m/z: Calculated $[M+H]^+$ 404.197; Found $[M+H]^+$ 404.15

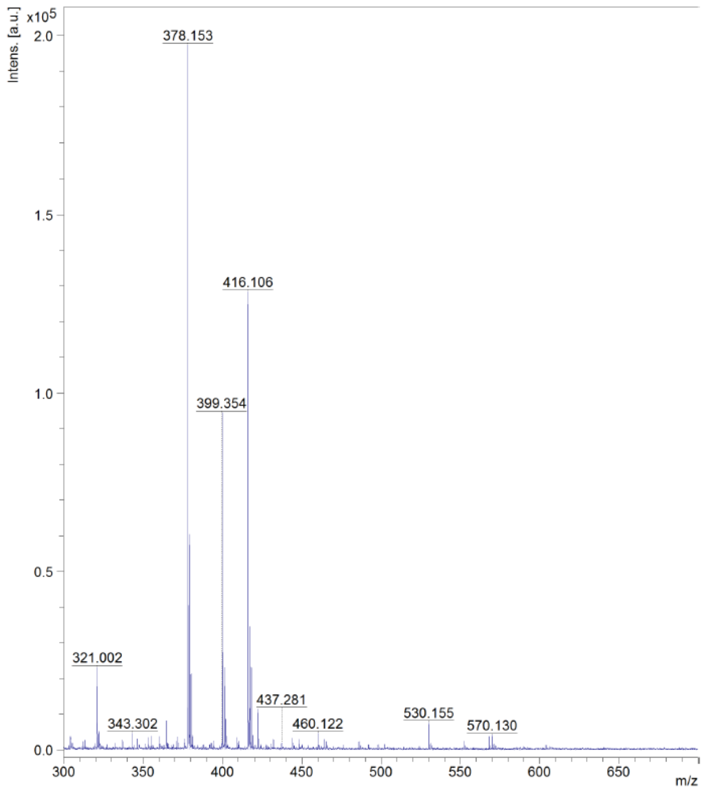

Figure S65. Mass analysis of peptide (*N*-Me)CWG-NH_2; MS (MALDI-TOF) m/z: Calculated $[M+H]^+$ 378.157; Found $[M+H]^+$ 378.15

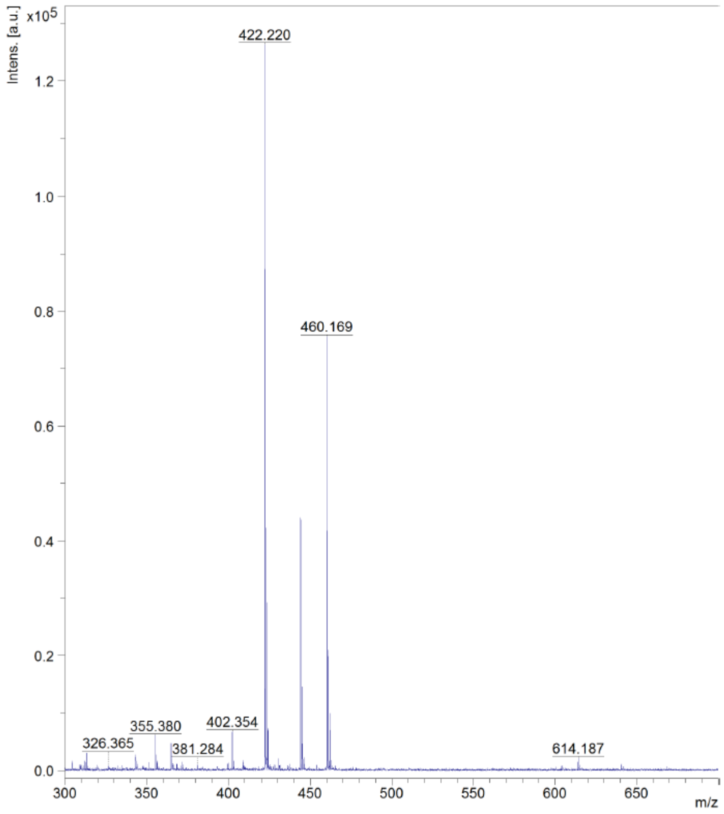

Figure S66. Mass analysis of peptide (*N*-Me)FWG-NH_2; MS (MALDI-TOF) m/z: Calculated $[M+H]^+$ 422.217; Found $[M+H]^+$ 422.22

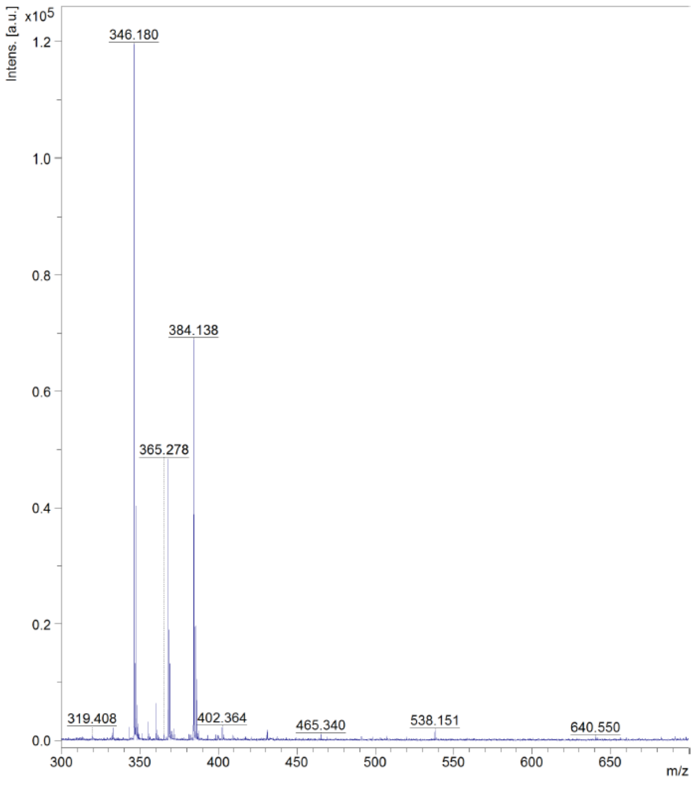

Figure S67. Mass analysis of peptide (*N*-Me)AWG-NH_2; MS (MALDI-TOF) m/z: Calculated $[M+H]^+$ 346.187; Found $[M+H]^+$ 346.18

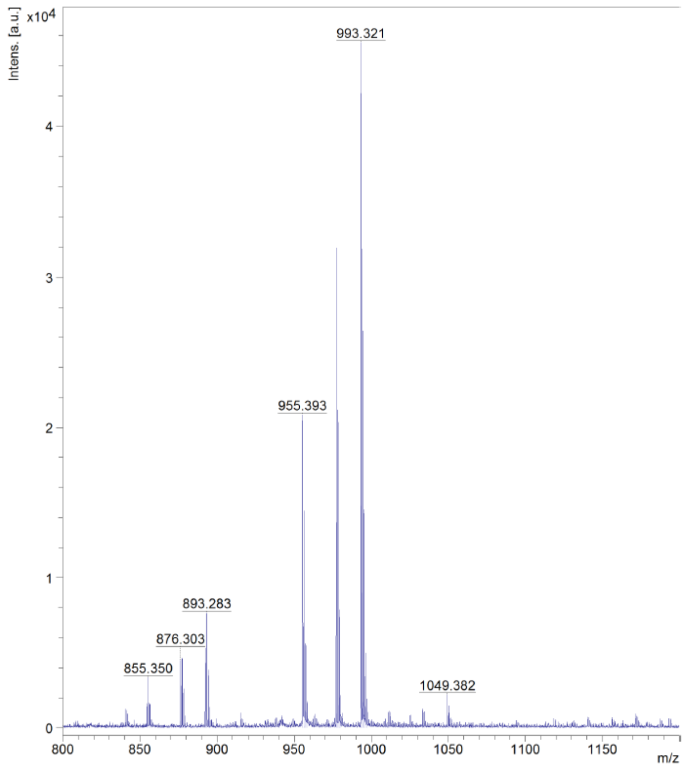

Figure S68. Mass analysis of peptide 1-SW1; MS (MALDI-TOF) m/z: Calculated $[M+H]^+$ 955.517; Found $[M+H]^+$ 955.393

NMR spectra of selected peptides

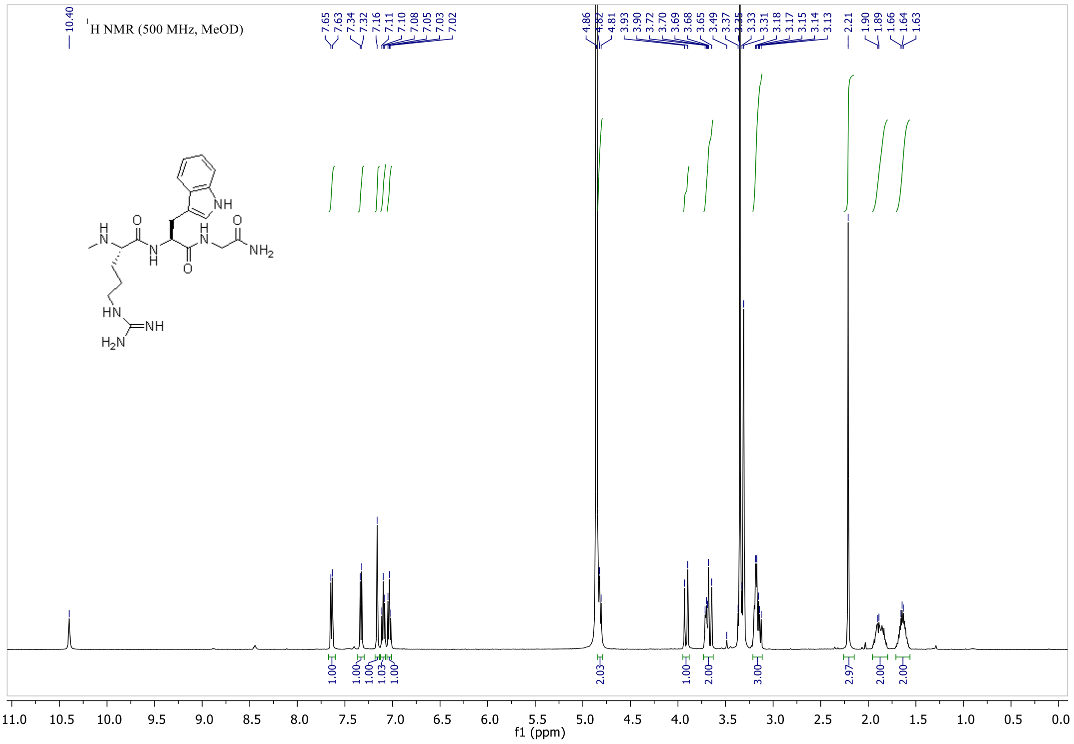

Figure S69. ^{1}H NMR spectra of peptide **1**.

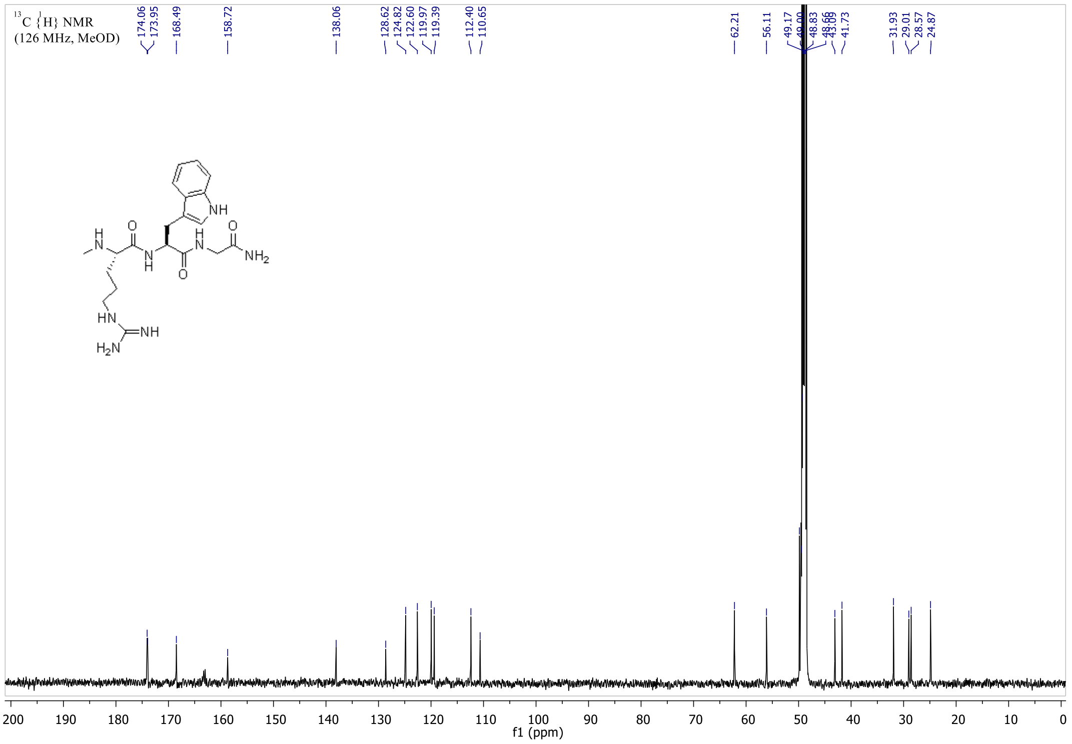

Figure S70. ^{13}C {^{1}H} NMR spectra of peptide **1**.

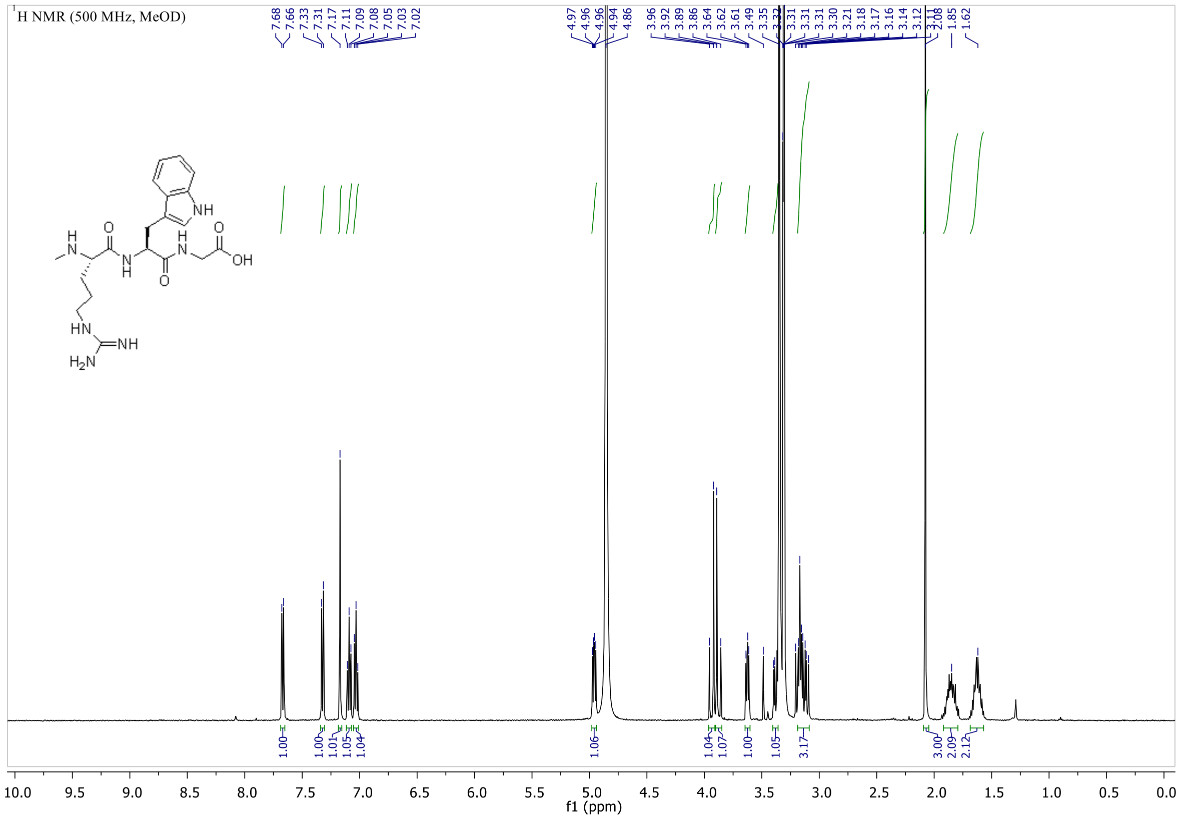

Figure S71. ^{1}H NMR spectra of peptide **2**.

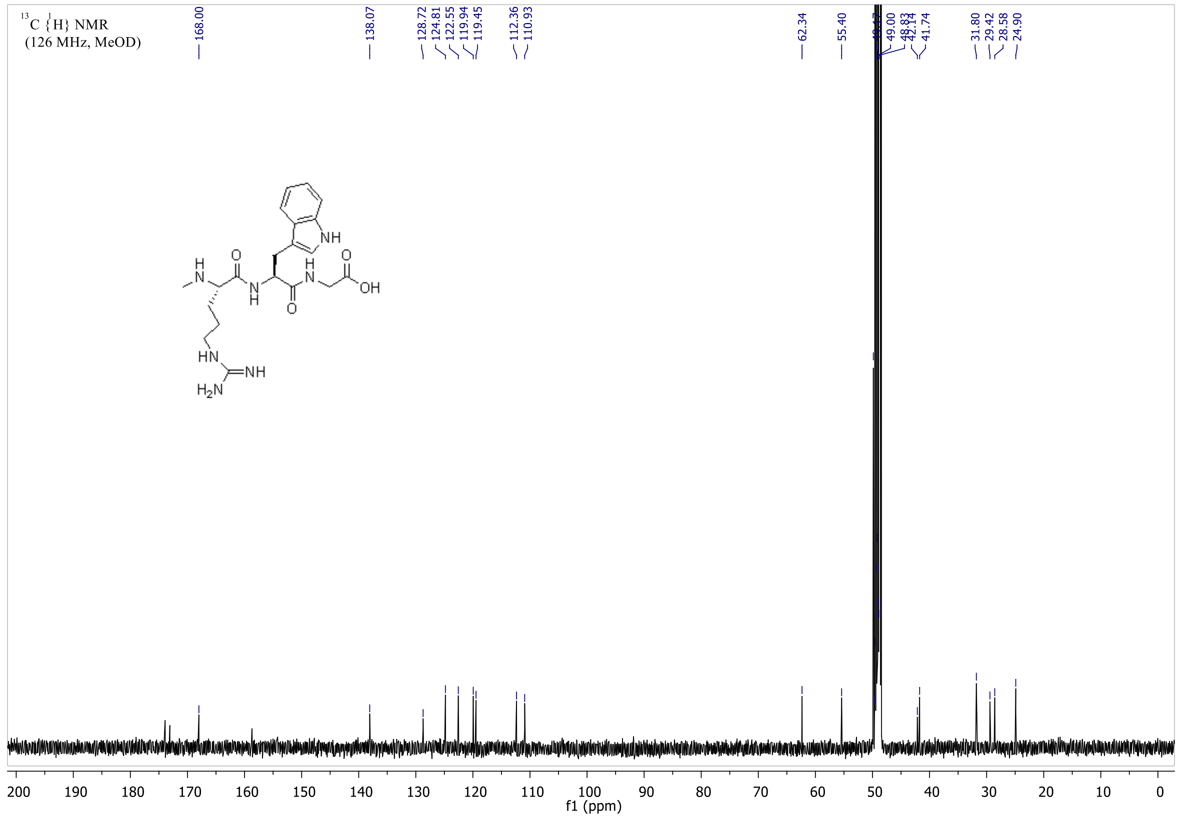

Figure S72. ^{13}C {^{1}H} NMR spectra of peptide **2**.

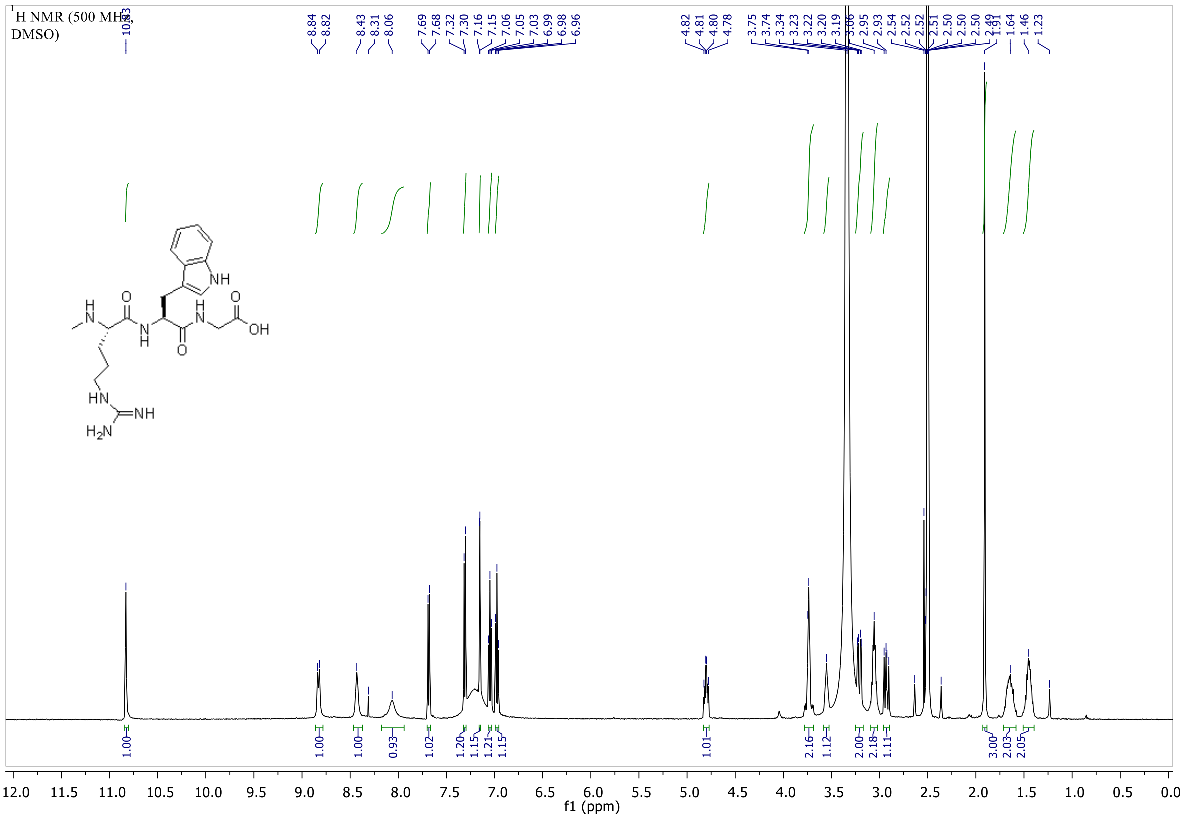

Figure S73. ^{1}H NMR spectra of peptide **2**.

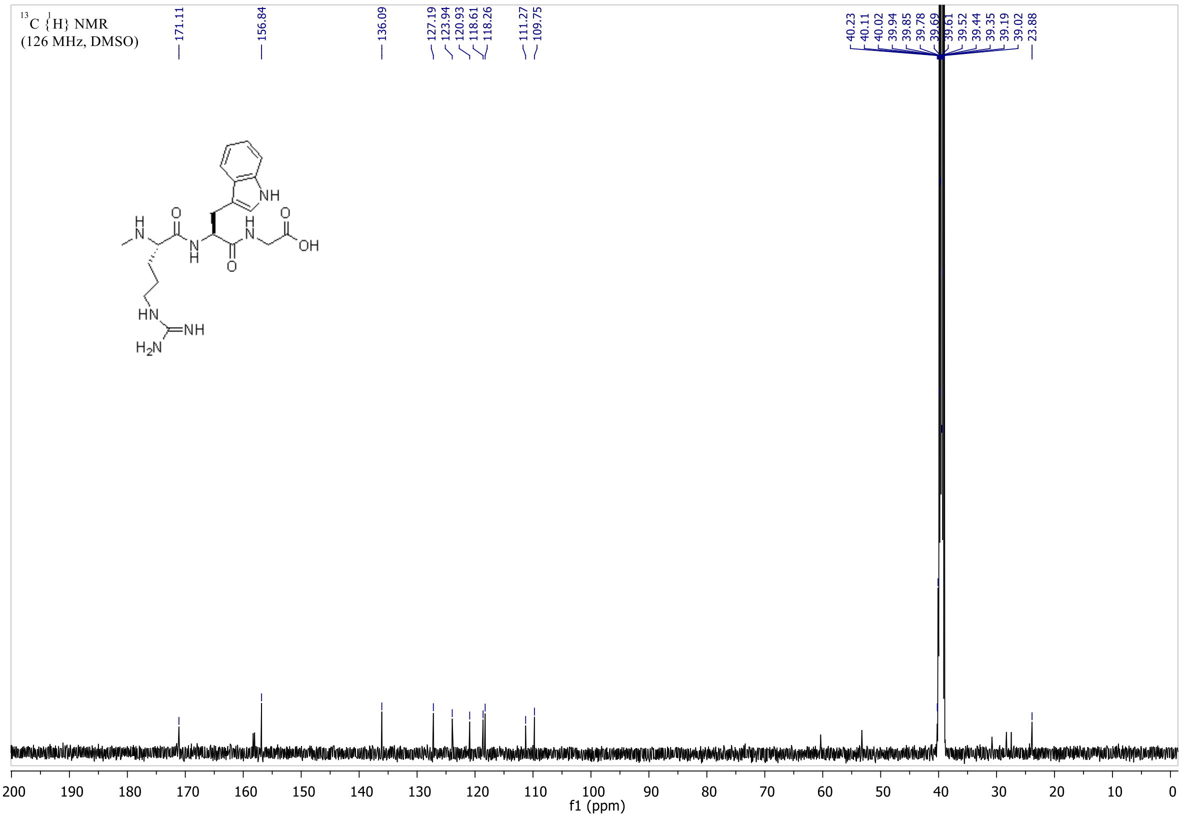

Figure S74. ^{13}C {^{1}H} NMR spectra of peptide **2**.

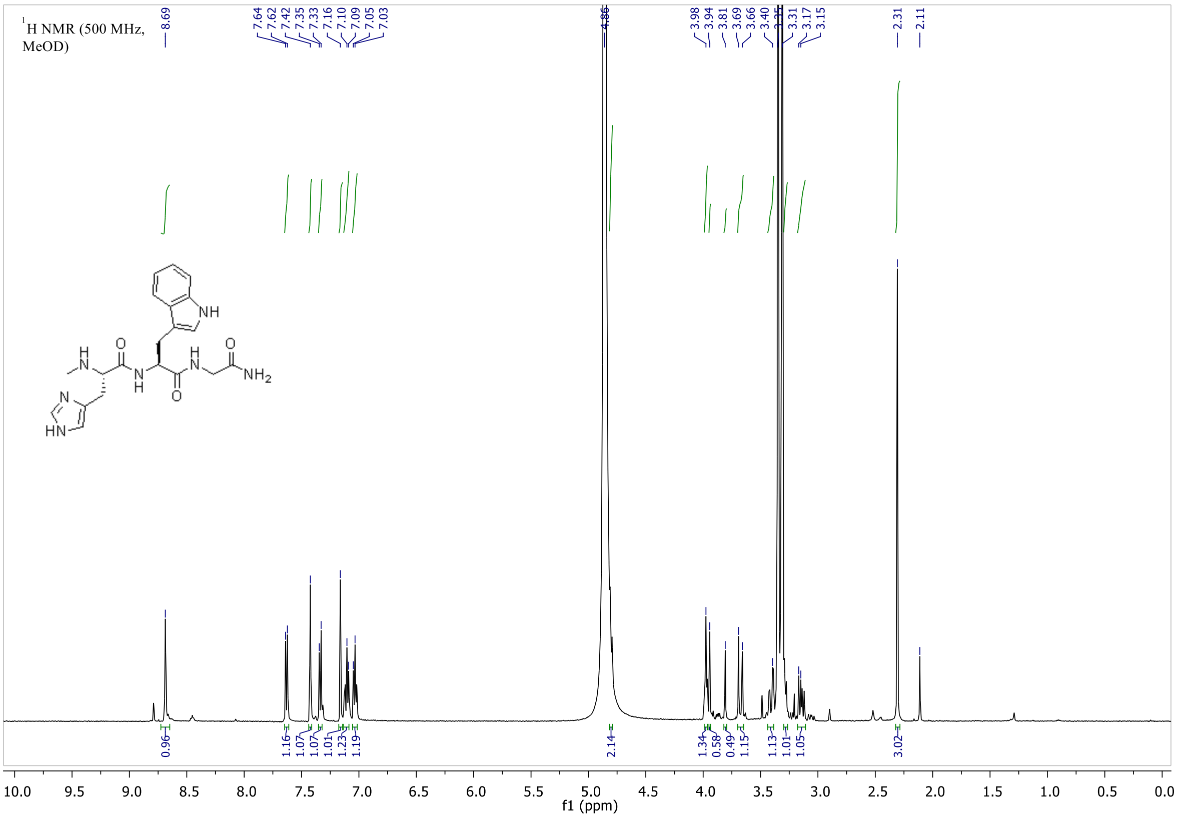

Figure S75. ^{1}H NMR spectra of peptide **3**.

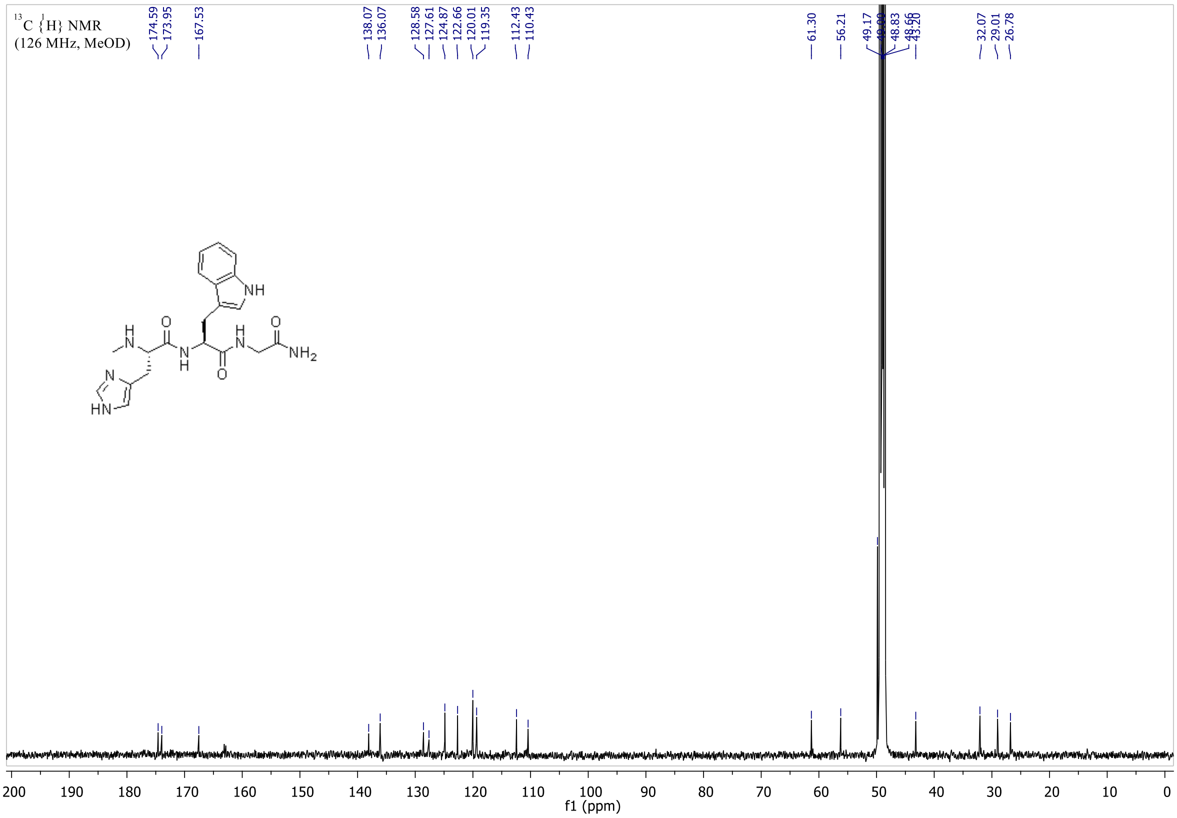

Figure S76. ^{13}C {^{1}H} NMR spectra of peptide **3**.

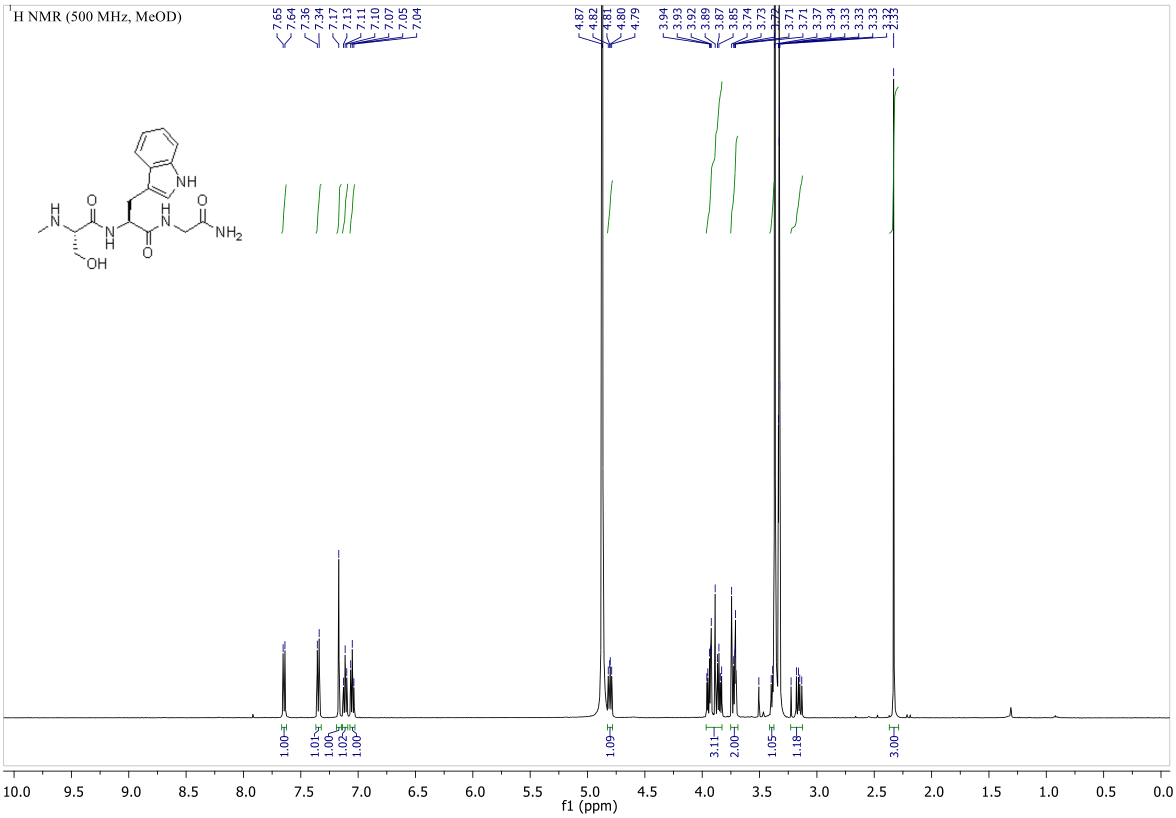

Figure S77. ^{1}H NMR spectra of peptide **4**.

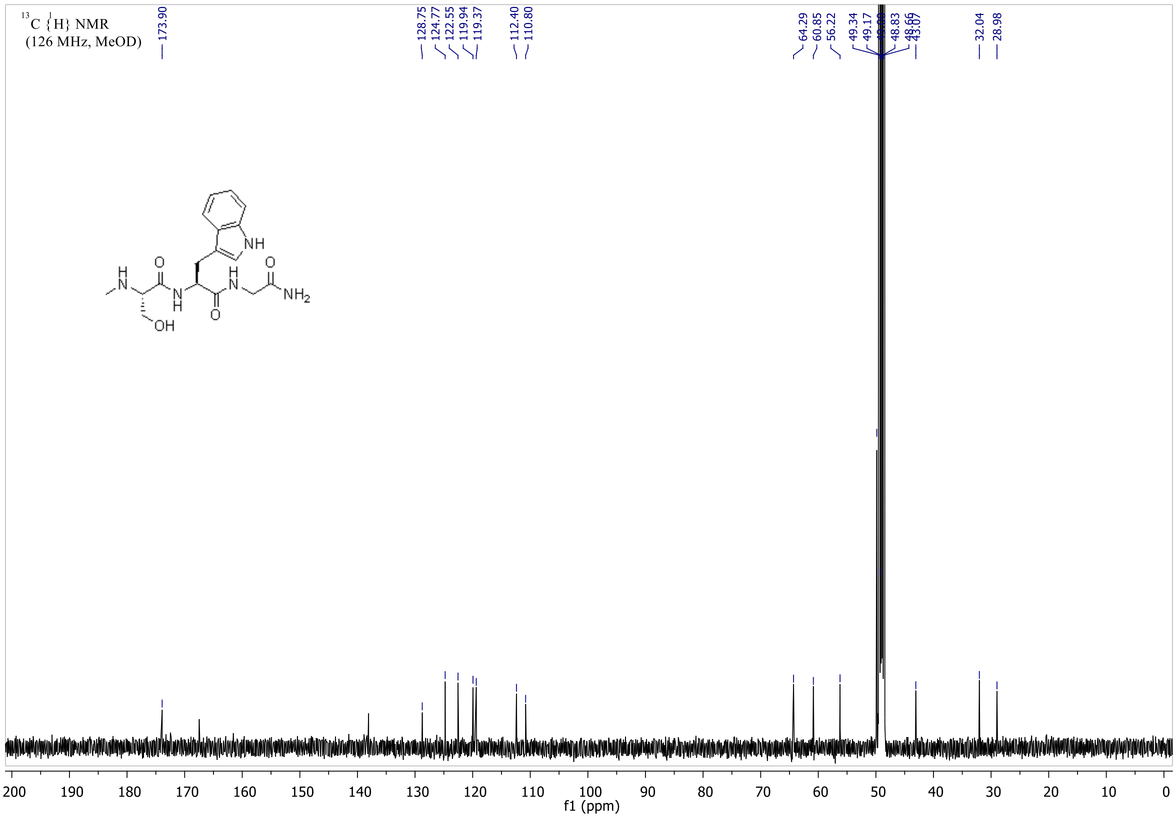

Figure S78. ^{13}C {^{1}H} NMR spectra of peptide **4**.

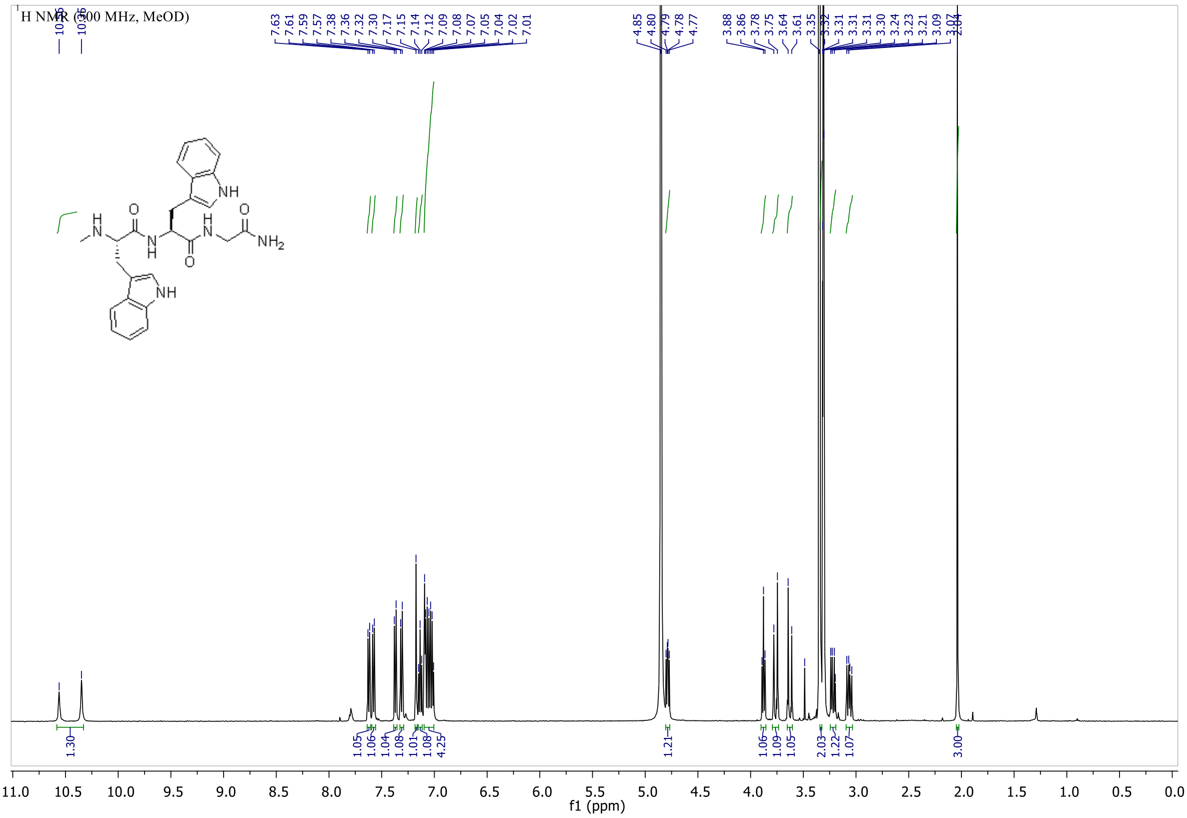

Figure S79. ^{1}H NMR spectra of peptide **5**.

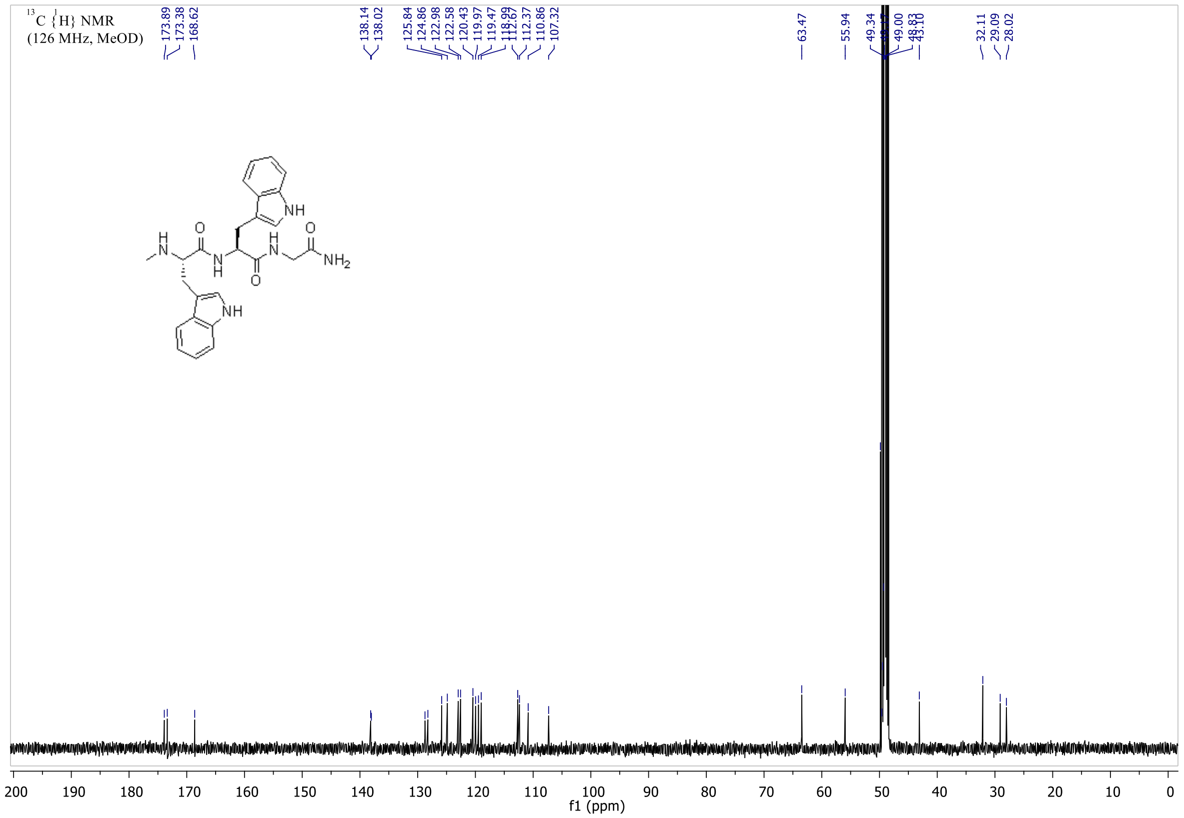

Figure S80. ^{13}C {^{1}H} NMR spectra of peptide **5**.

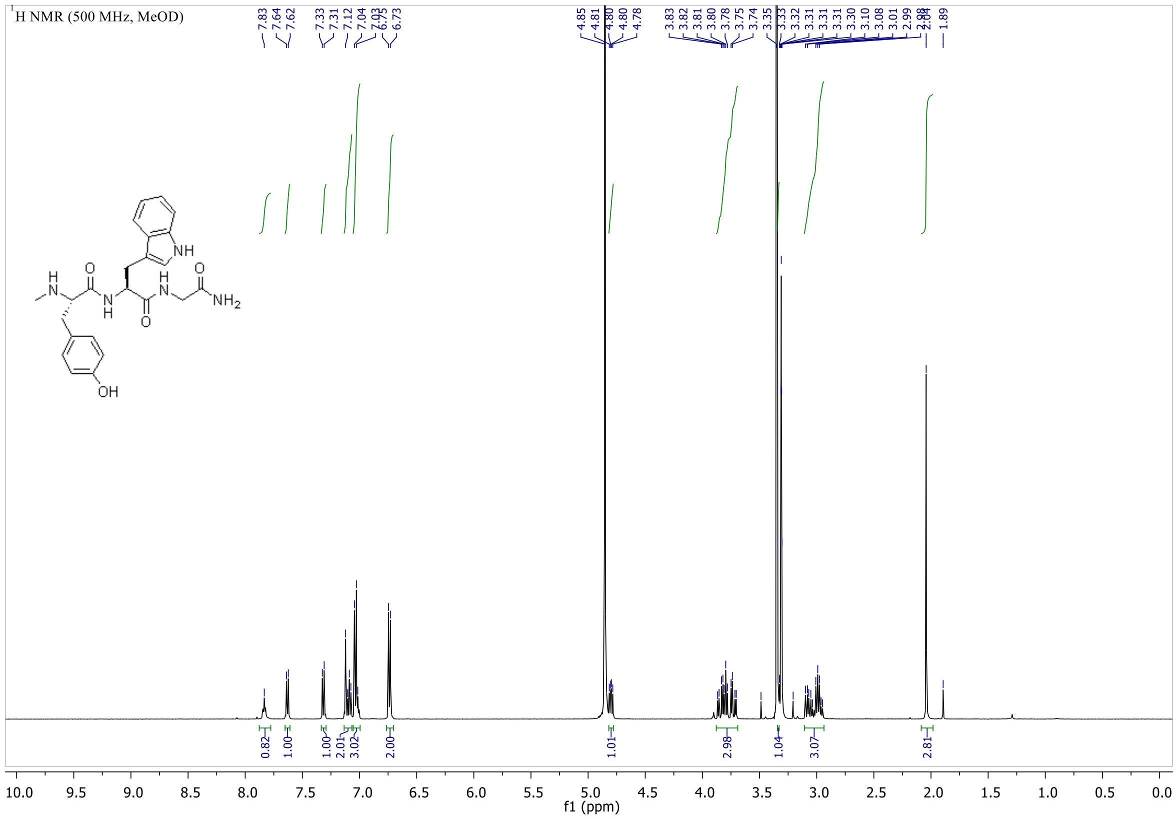

Figure S81. ^{1}H NMR spectra of peptide **6**.

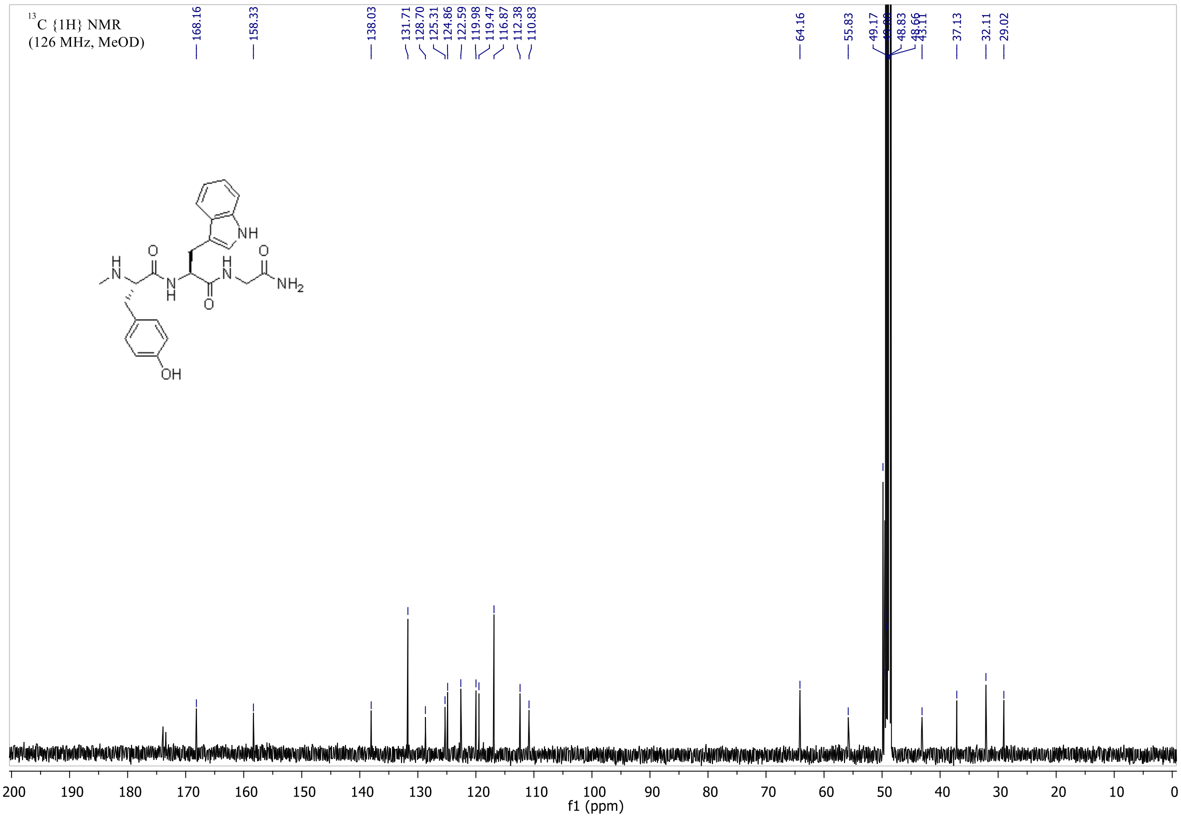

Figure S82. ^{13}C {^{1}H} NMR spectra of peptide **6**.

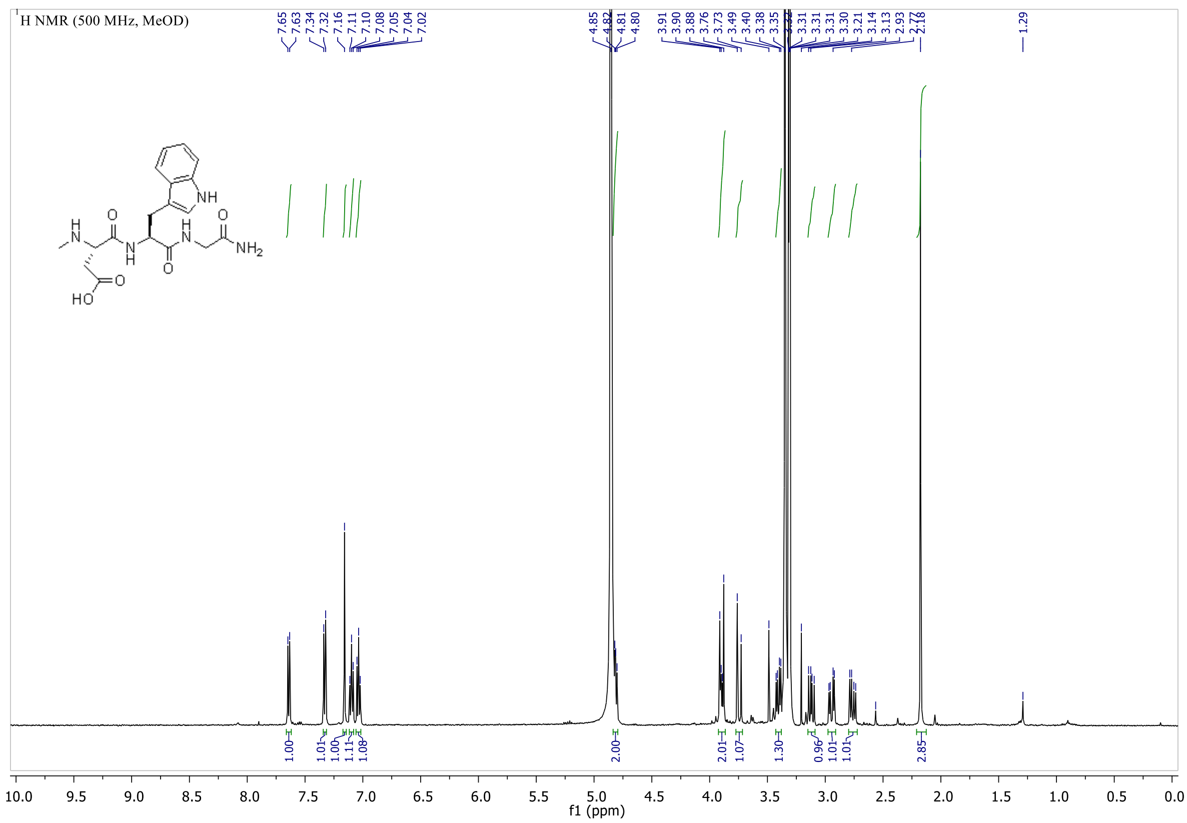

Figure S83. ^{1}H NMR spectra of peptide **7**.

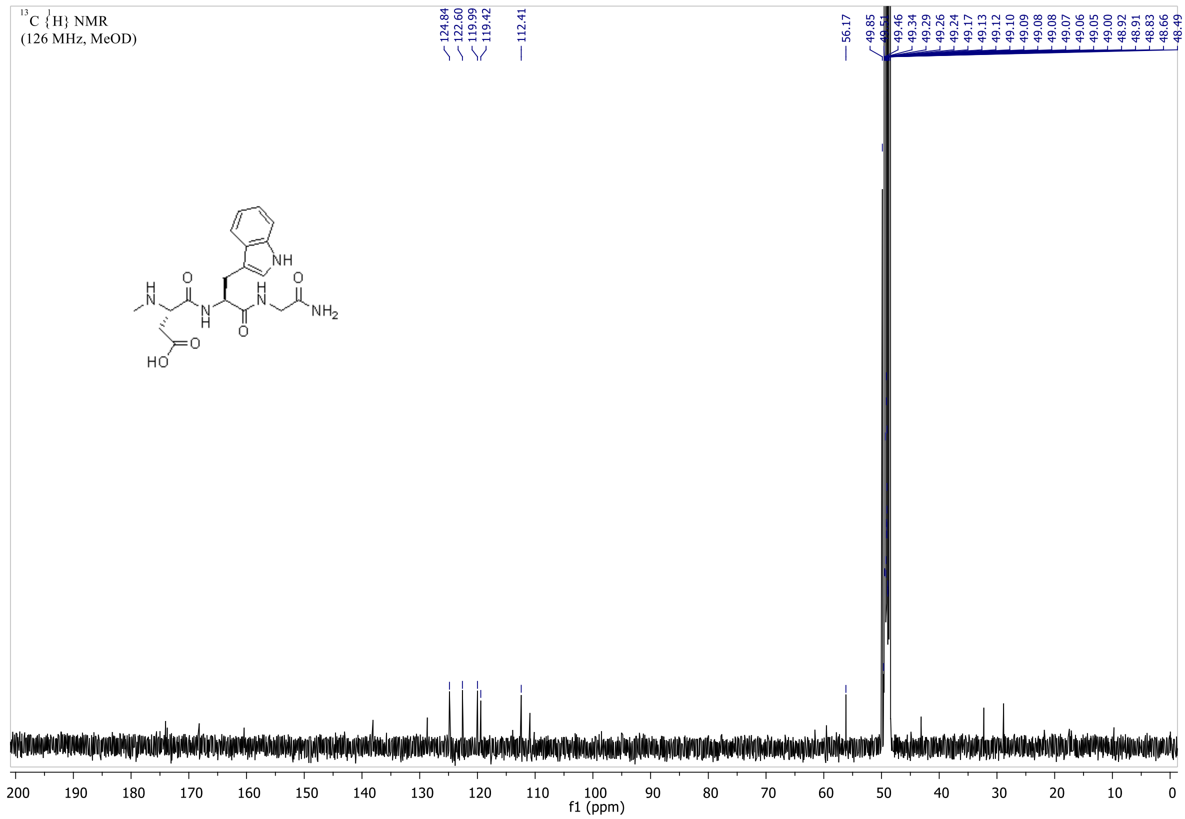

Figure S84. ^{13}C {^{1}H} NMR spectra of peptide **7**.

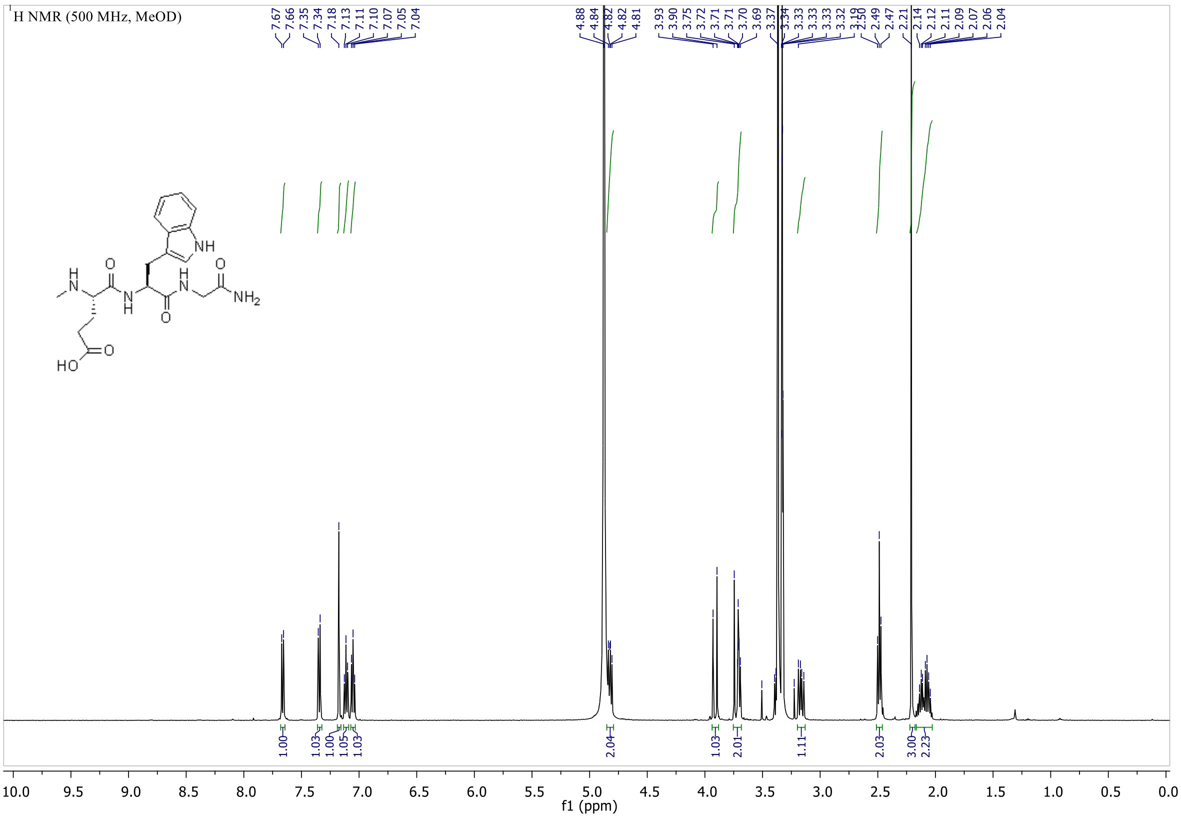

Figure S85. ^{1}H NMR spectra of peptide **8**.

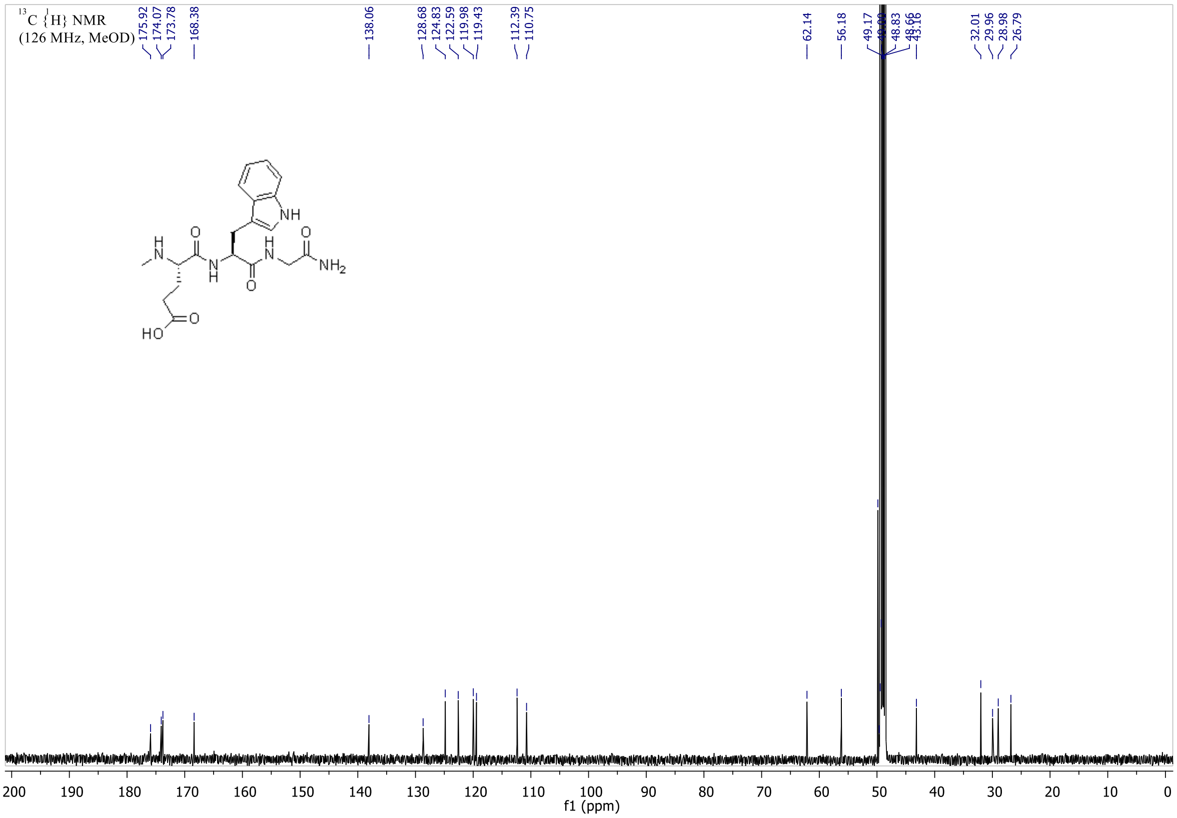

Figure S86. $^{13}C\ \{^{1}H\}$ NMR spectra of peptide **8**.

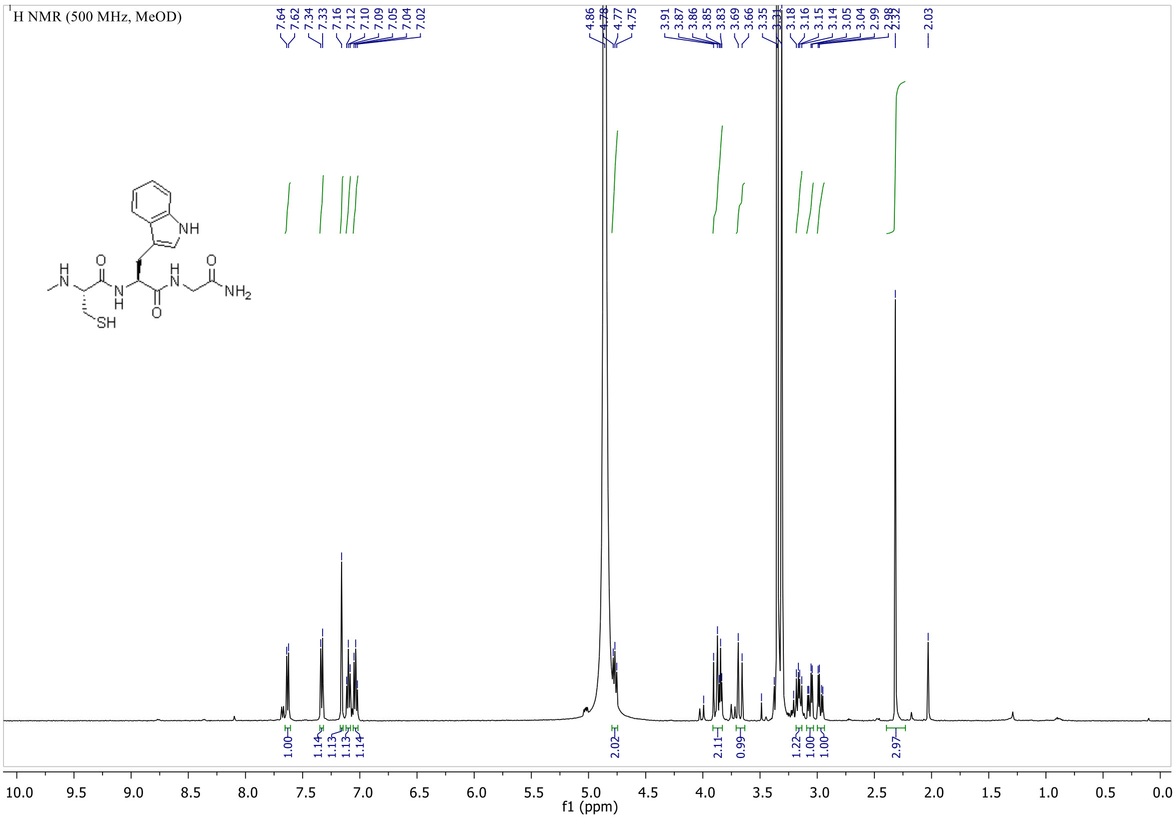

Figure S87. ^{1}H NMR spectra of peptide **9**.

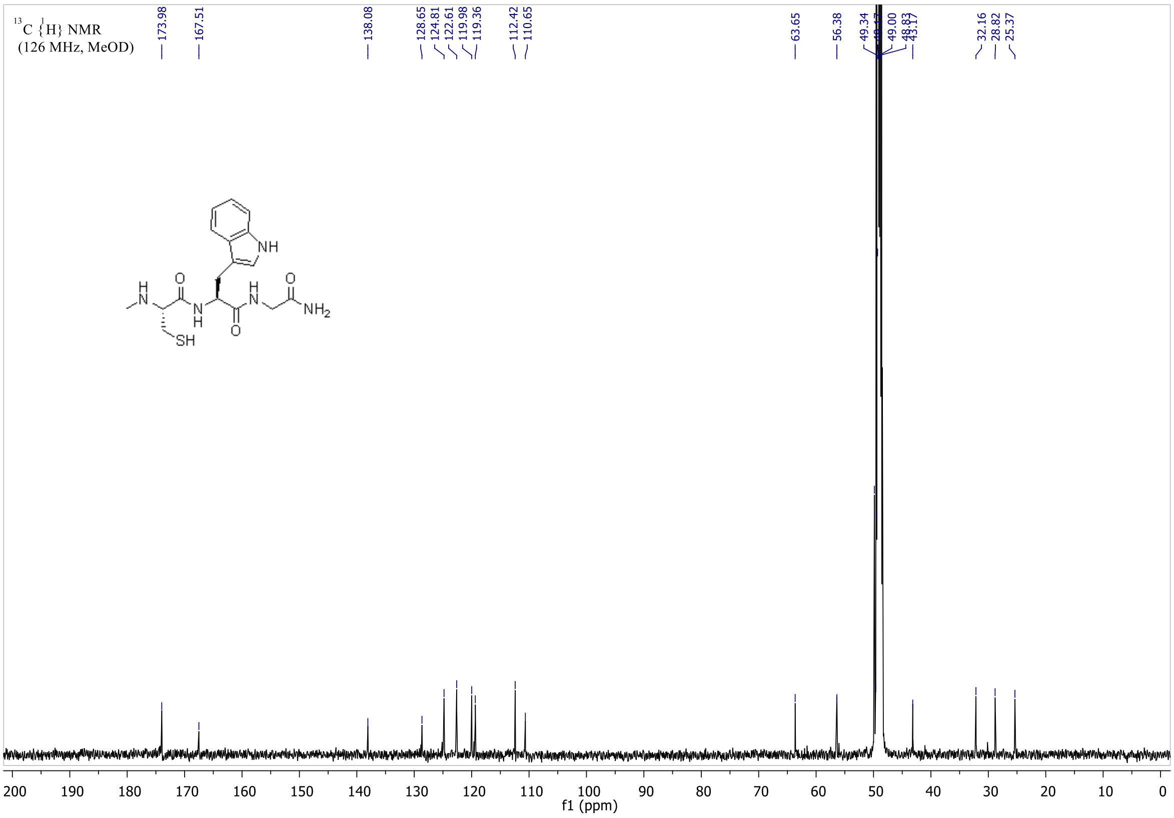

Figure S88. ^{13}C {^{1}H} NMR spectra of peptide **9**.

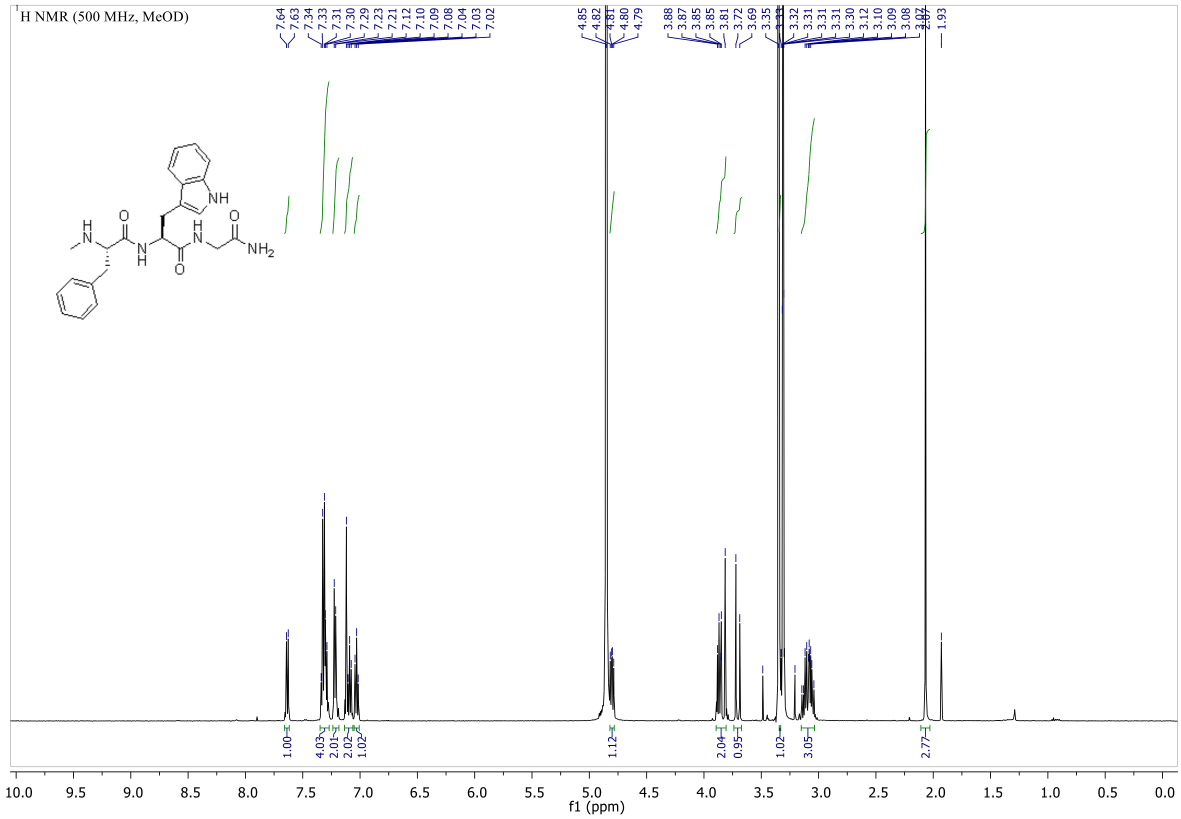

Figure S89. ^{1}H NMR spectra of peptide **10**.

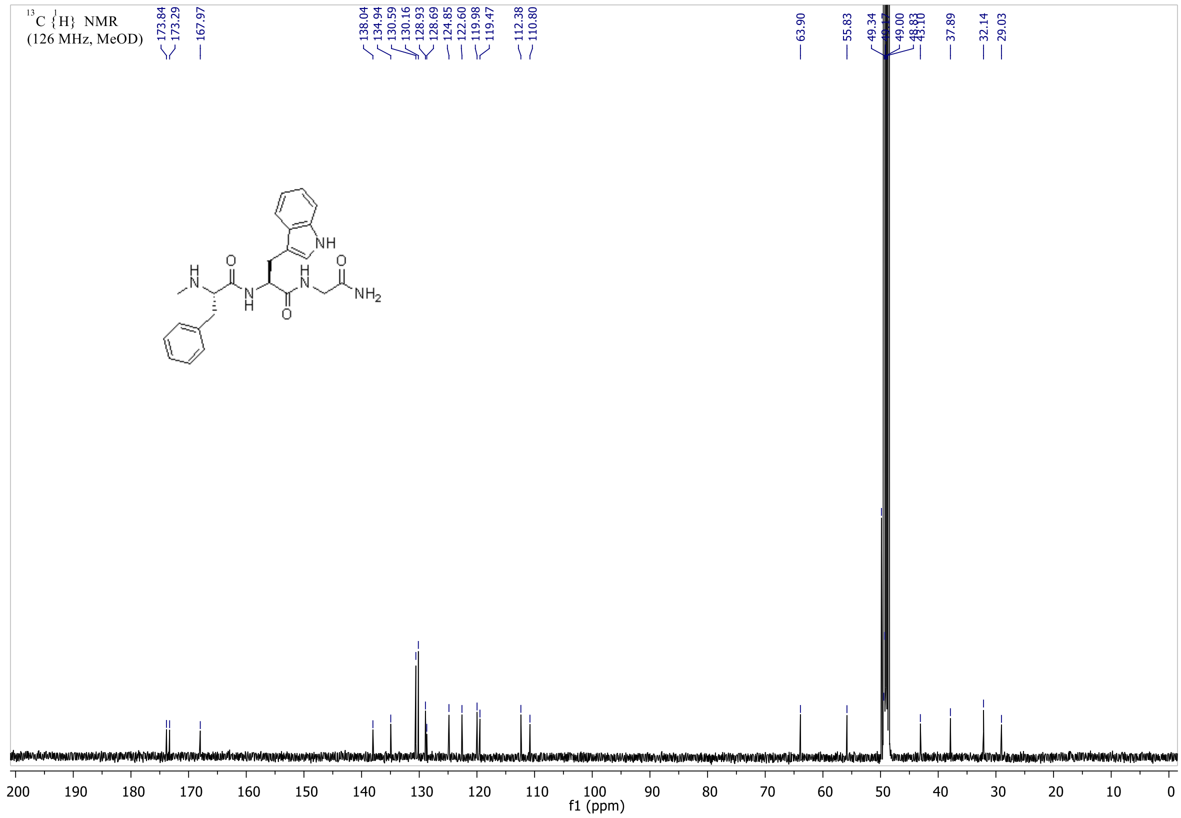

Figure S90. ^{13}C {^{1}H} NMR spectra of peptide **10**.

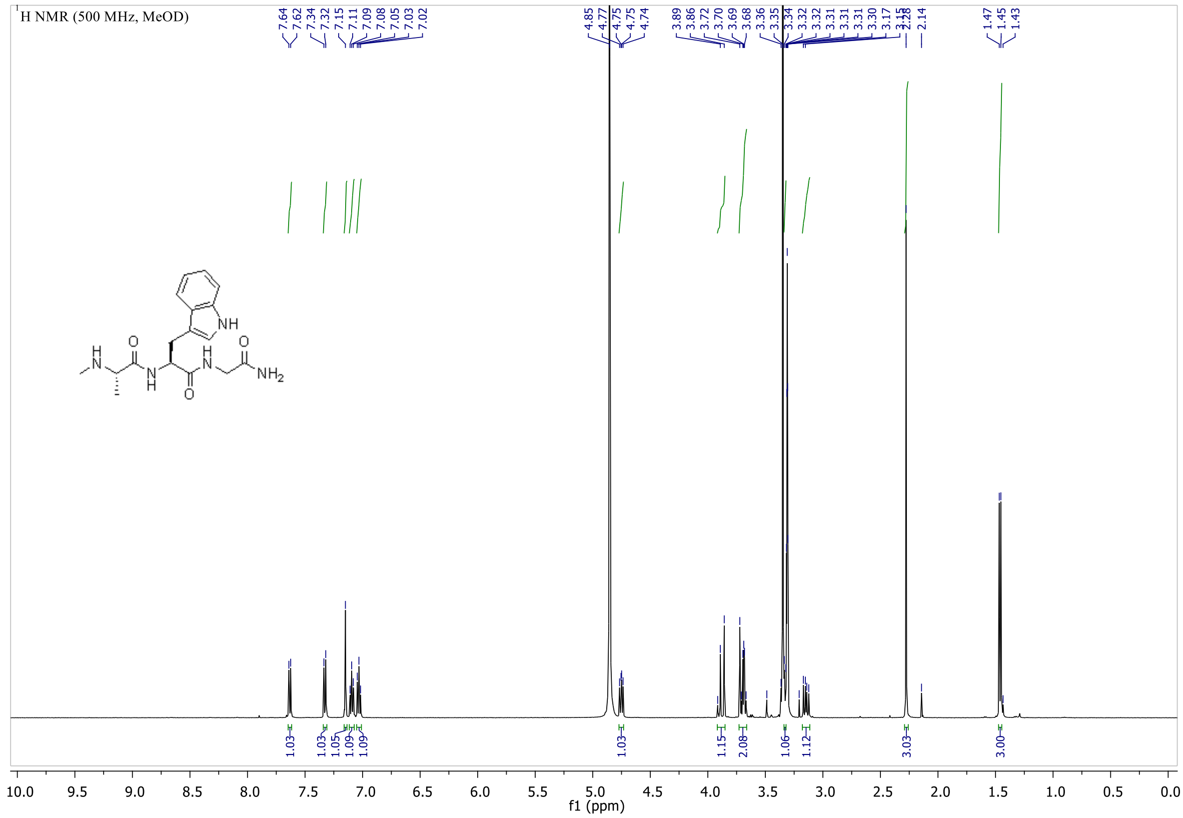

Figure S91. ^{1}H NMR spectra of peptide **11**.

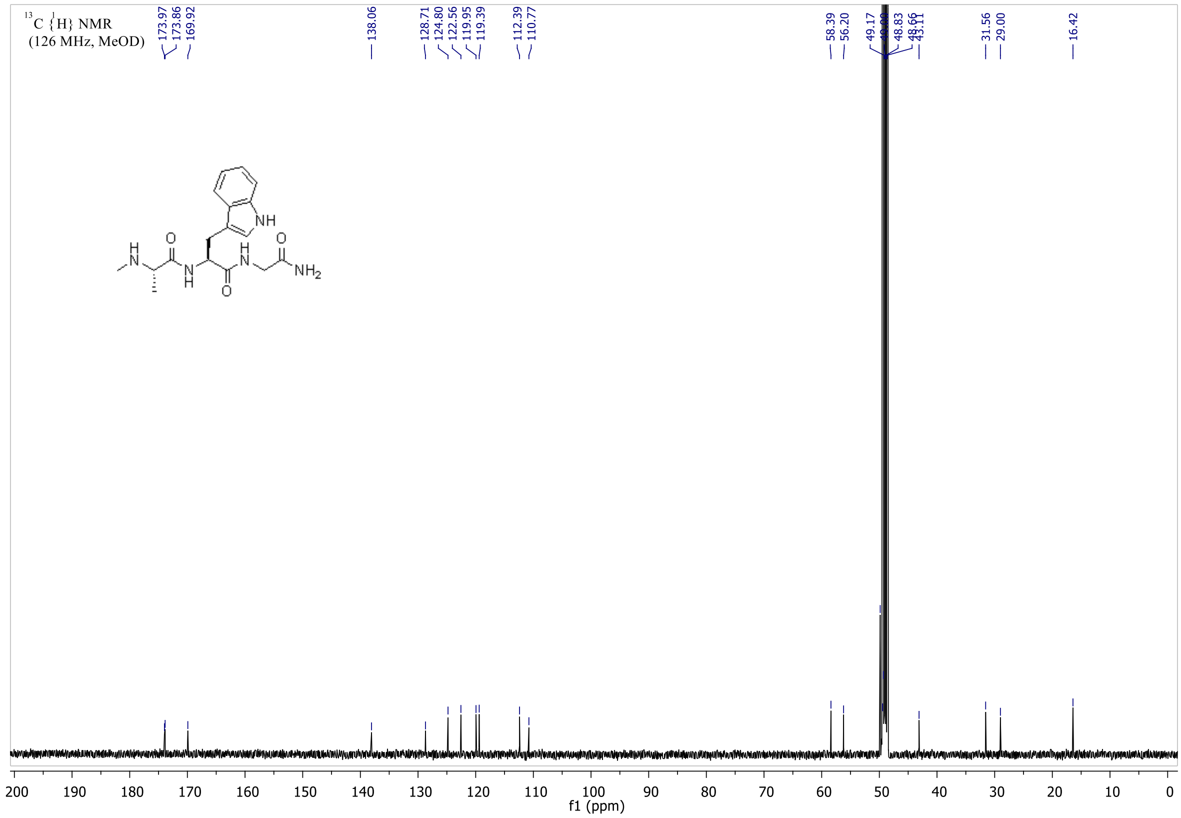

Figure S92. ^{13}C {^{1}H} NMR spectra of peptide **11**.
